# Tandem Hock and Friedel–Crafts reactions allowing an expedient synthesis of a cyclolignan-type scaffold

**DOI:** 10.3762/bjoc.20.15

**Published:** 2024-01-25

**Authors:** Viktoria A Ikonnikova, Cristina Cheibas, Oscar Gayraud, Alexandra E Bosnidou, Nicolas Casaretto, Gilles Frison, Bastien Nay

**Affiliations:** 1 Laboratoire de Synthèse Organique, Ecole Polytechnique, CNRS, ENSTA, Institut Polytechnique de Paris, F-91128 Palaiseau, France; 2 Laboratoire de Chimie Moléculaire, Ecole Polytechnique, CNRS, Institut Polytechnique de Paris, F-91128 Palaiseau, France; 3 Sorbonne Université, CNRS, Laboratoire de Chimie Théorique, 75005 Paris, France

**Keywords:** 1-aryltetralines, Friedel–Crafts reaction, Hock rearrangement, oxidative cleavage, tandem reactions

## Abstract

The Hock cleavage, which is compatible with tandem processes, was applied to the synthesis of 1-aryltetralines through a one-pot transformation from readily available benzyl(prenyl)malonate substrates. After the photooxygenation of the prenyl moiety, the resulting hydroperoxide was directly engaged in a Hock cleavage by adding a Lewis acid. The presence of an aromatic nucleophile in the reaction mixture and that of a benzyl moiety on the substrate resulted in tandem Friedel–Crafts reactions to form the 1-aryltetraline products. These compounds share a close analogy to the cyclolignan natural products. Experimental observations and a DFT study support the involvement of an aldehyde intermediate during the Friedel–Crafts reactions, rather than an oxocarbenium.

## Introduction

**Scheme 1:**
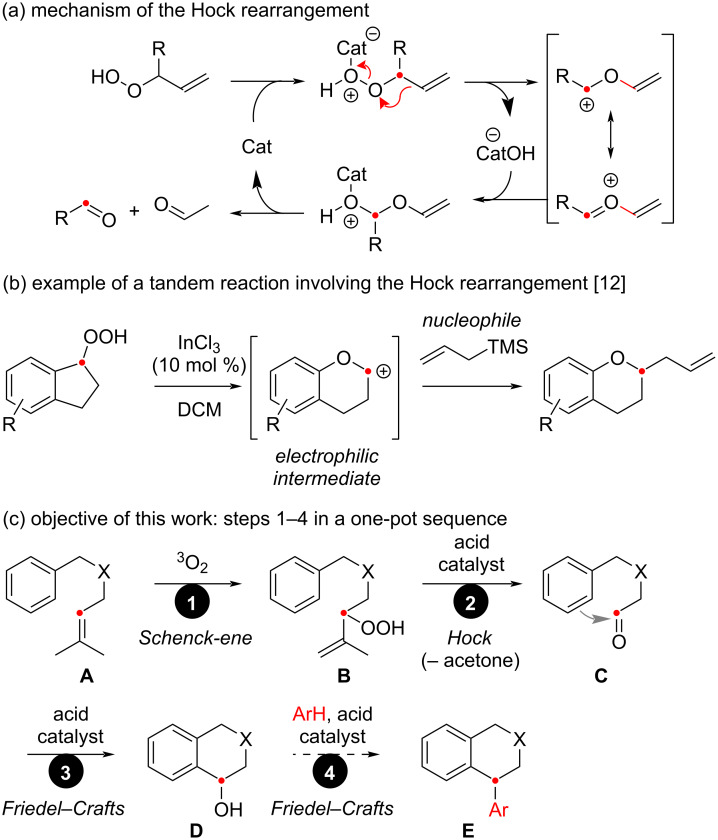
The Hock rearrangement: (a) General mechanism (substituents are omitted); (b) Example of previous tandem process; (c) Objective of this work.

The Hock cleavage [1] consists in the acid-catalyzed rearrangement of organic hydroperoxides, leading to the oxidative cleavage of a C–C bond adjacent to the hydroperoxide group (Scheme 1a). The best-known application of this reaction is the cumene process, which allows the production of millions of tons of phenol each year [2]. The reaction has also been used in an industrial synthesis of artemisinin [3]. Allylic hydroperoxides are excellent substrates for such reactions, affording electrophilic carbonyl derivatives susceptible to react with nucleophiles in the acidic reaction mixture [4–8]. Consequently, the Hock rearrangement is likely to be part of tandem processes involving this carbonyl function [9–11], in the presence of a nucleophilic species. Recently, we applied this idea to the rearrangement of 1-indanyl hydroperoxides into 2-substituted chromane derivatives, involving the nucleophilic allylation of the rearranged oxocarbenium intermediate (Scheme 1b) [12–13]. Furthermore, it is interesting to mention that allylic hydroperoxides are conveniently produced by the photooxygenation of alkene substrates [14–16]. Taking all these informations together, since alkenes can be easier intermediates than aldehydes to handle, we envisaged to use a prenyl (= 3-methyl-2-buten-1-yl) group as an aldehyde surrogate readilly unmasked under Hock cleavage conditions. The oxidative cleavage of this alkene would not only release the aldehyde group, but also volatile acetone originated from the traceless isopropylidene motif. Overall, a three-reaction process will thus be performed in one pot (Scheme 1c), successively involving a Schenck-ene photooxygenation of an alkene **A**, an acid-catalyzed Hock cleavage of hydroperoxide **B** generating an aldehyde derivative **C**, and an acid-catalyzed Friedel–Crafts reaction in the presence of an aromatic nucleophile leading to **D** [17]. In principle, a second Friedel–Crafts reaction is possible upon elimination of the resulting benzylic alcohol on **D**, allowing another arylation forming **E** [18]. This complex sequence of transformations is herein applied to the synthesis of 1-aryltetralines, analogues of cyclolignan natural products having important medicinal applications [19–20].

## Results and Discussion

**Scheme 2:**
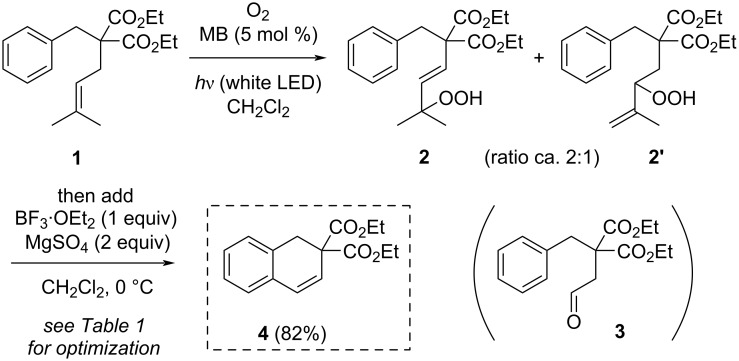
One-pot conversion of substrate **1** into dihydronaphthalene **4**.

To test the feasibility of this reaction sequence, the aromatic substrate **1** readily accessible by the prenylation of commercial diethyl benzylmalonate [21] was first used. The photooxygenation of **1** was performed in CH_2_Cl_2_ in the presence of methylene blue (MB) as a photosensitizer (tetraphenylporphyrin performed equally well but was harder to separate from the products) and irradiated by white LED light under an atmosphere of oxygen. It led to a mixture of unseparable regioisomeric allylic hydroperoxides **2** and **2’** (≈2:1) that were not isolated for instability reasons, but directly engaged in the Hock cleavage step under acidic conditions (Scheme 2 and Table 1). In the presence of trifluoroacetic acid (TFA, 1 equiv; Table 1, entry 1) or BF_3_·OEt_2_ (1 equiv; Table 1, entry 2), the rearrangement of **2** and **2’** led to a complex mixture of products including aldehyde **3**, the typical product of the Hock rearrangement. Since the Hock rearrangement results in the formation of a molecule of water, we attempted to add a water scavenger to the reaction solution. Additives like molecular sieves 4 Å or Na_2_SO_4_ (2 equiv) did not show any improvement (Table 1, entry 3), while MgSO_4_ (2 equiv) had a stunning effect leading to the isolation of dihydronaphthalenic product **4** in 82% yield (Table 1, entry 4, these conditions will later be taken as the reference). Trying to reduce this reagent stoichiometry only resulted in a poor yield of **4** and in the isolation of aldehyde **3** in 42% yield (Table 1, entry 5). By contrast, in the sole presence of MgSO_4_, no reaction was observed (Table 1, entry 6). Furthermore, Yb(OTf)_3_ or ZnCl_2_ mainly resulted in aldehyde **3** (Table 1, entries 7 and 8), while AlCl_3_ performed well with an 83% yield of **4** (Table 1, entry 9). This optimization validated the expected tandem sequence of photooxygenation, Hock rearrangement and Friedel–Crafts reaction, which is supposed to proceed through aldehyde **3** (see further discussion below on the reaction mechanism).

**Table 1:** Reaction optimization toward **4** (see also Scheme 2).

	Catalyst	Additive (water trap)	Product(s)^a^

1	TFA (1 equiv)	–	c.m.
2	BF_3_·OEt_2_ (1 equiv)	–	c.m.
3	BF_3_·OEt_2_ (1 equiv)	Na_2_SO_4_ (2 equiv) or MS 4 Å (100 wt %)	c.m.
4	BF_3_·OEt_2_ (1 equiv)	MgSO_4_ (2 equiv)	**4** (82%)^b^
5	BF_3_·OEt_2_ (0.2 equiv)	MgSO_4_ (1 equiv)	**3** (42%), **4** (8%)^c^
6	–	MgSO_4_ (2 equiv)	n.r.
7	Yb(OTf)_3_ (2 equiv)	–	**3** (36%)^c^
8	ZnCl_2_ (2 equiv)	–	**3** (n.d.)
9	AlCl_3_ (2 equiv)	–	**4** (83%)^c^

^a^Abbreviations: c.m.: complex mixture; n.r.: no reaction; n.d.: not determined. ^b^Isolated yield from starting material **1**. ^c^NMR yield in the presence of 1,2-dichloroethane as an internal reference, calculated from starting material **1**.

**Scheme 3:**
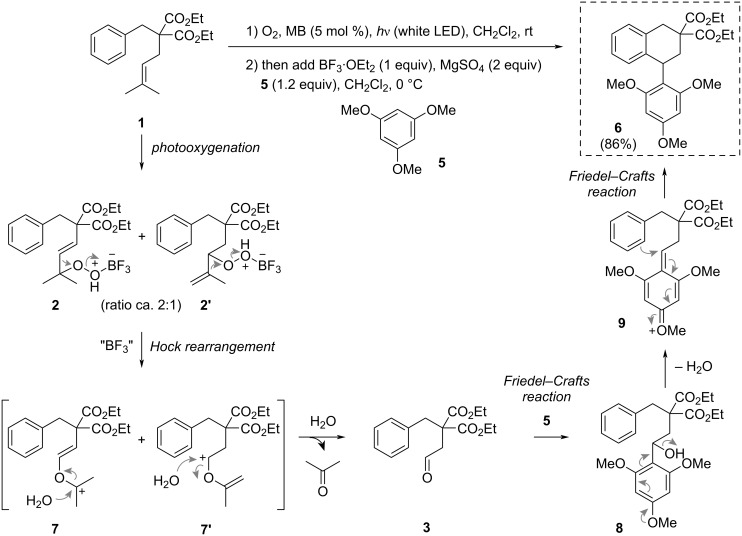
One-pot conversion of substrate **1** into 1-aryltetraline structure **6**, and the proposed mechanism for its formation.

**Figure 1:**
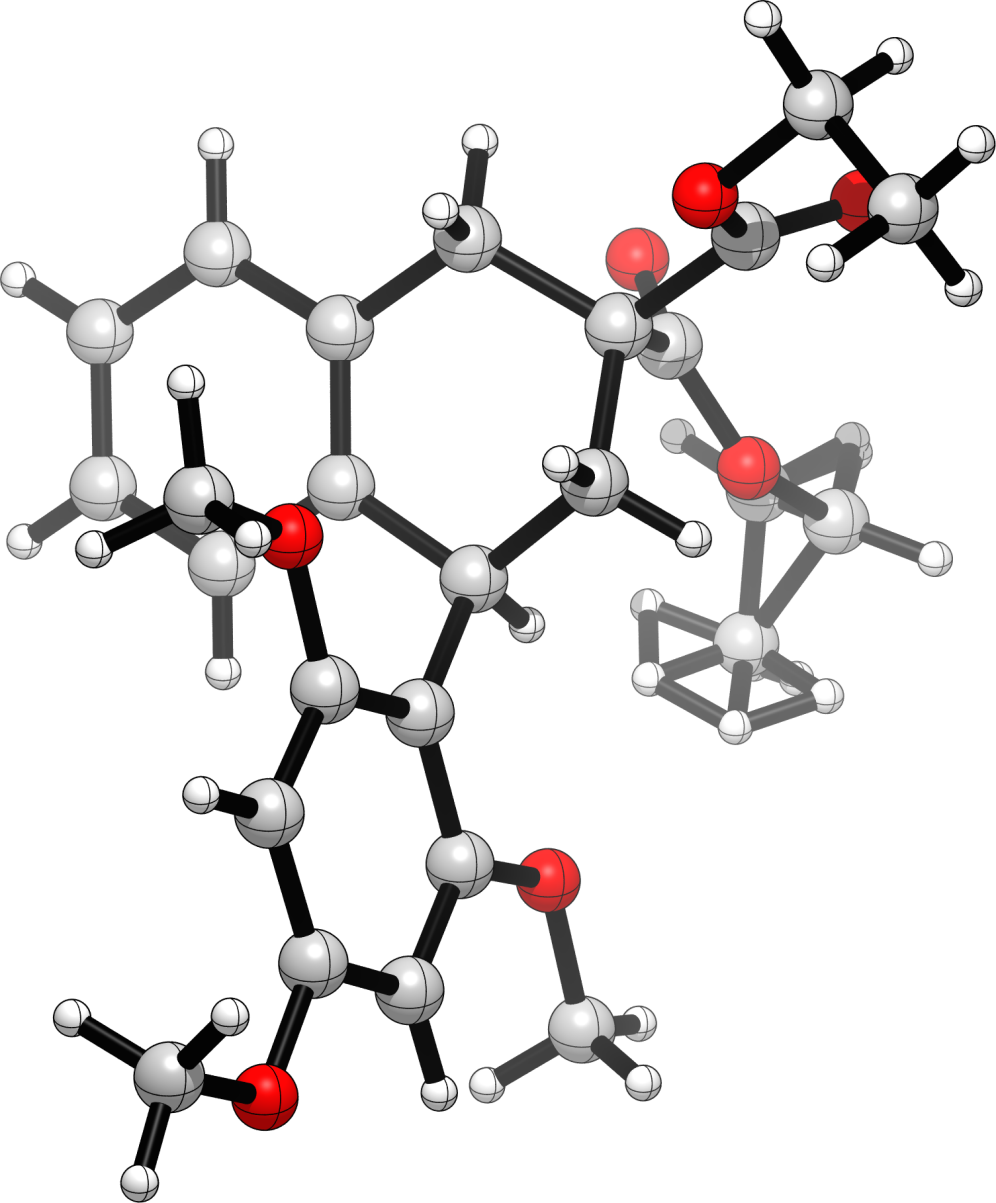
X-ray crystallographic structure of product **6** (CCDC 2301977). The structure shows one disordered ethyl ester group (backward).

To complete this exploratory work, we envisaged to add an external aromatic nucleophile to the reaction mixture, namely 1,3,5-trimethoxybenzene (**5**), which was susceptible to compete with the internal phenyl group during the Friedel–Crafts reaction step. Strickingly, under the optimized conditions (see Table 1, entry 4), the reaction in the presence of **5** (1.2 equiv) led to product **6** in 86% yield (Scheme 3). During this reaction, compound **4** was not formed, but the formation of aldehyde **3** could be observed during TLC monitoring. Furthermore, engaging previous dihydronaphthalene **4** in a Friedel–Crafts reaction with **5** in the presence of BF_3_·OEt_2_ and MgSO_4_ did not afford product **6**. These observations are in agreement with a mechanism involving a first Friedel–Crafts reaction of aldehyde **3** (oxocarbenium intermediate **7'** is also a good candidate for this reaction, see next paragraph) with 1,3,5-trimethoxybenzene (**5**), leading to intermediate **8** (Scheme 3). This last compound was too reactive to be isolated, presumably leading to a quinone methide intermediate **9** upon elimination of the hydroxy group. This highly electrophilic species could trigger a second intramolecular Friedel–Crafts reaction leading to **6**. The cyclic connectivity of **6** was determined by bidimensional NMR experiments, incidentally showing a broadening of the signals of *ortho*-methoxy substituents as previously observed by others [22–23], and demonstrating the high rotational barrier constraining the aryl substituent. This structure was unambiguously confirmed by X-ray crystallographic analysis (Figure 1).

**Scheme 4:**
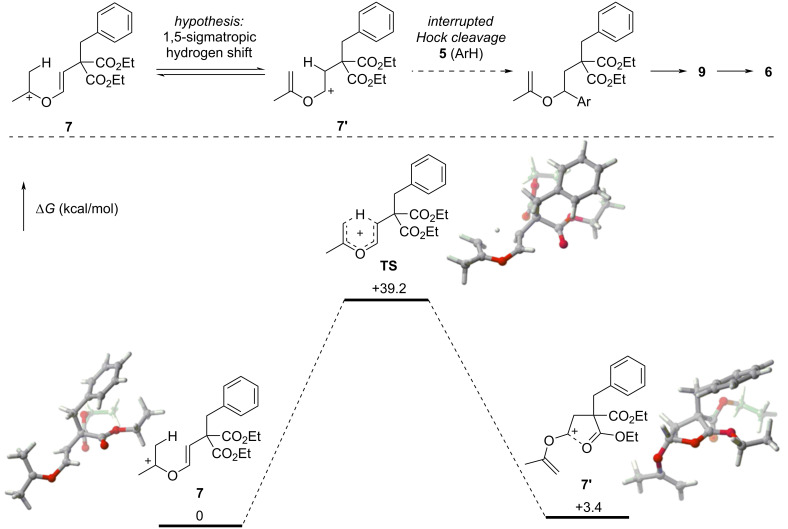
Free-energy profile of the hypothesized [1,5]-sigmatropic hydrogen shift between **7** and **7’**, (IEFPCM(CH_2_Cl_2_)-M06/6-311++G(2d,2p)//M06/6-31G(d,p) level of theory).

During this work we have been intrigued by the possible direct involvement of oxocarbenium species **7** and **7’** in a Friedel–Crafts reaction with **5**, to explain the formation of **6** through an interrupted Hock cleavage mechanism. Confronting the yield of this transformation (86%) and the fact that only **7’**, originating from **2’**, could be an intermediate towards **6** in this alternative mechanism, we envisionned a possible interconversion of **7** and **7’** through a [1,5]-sigmatropic rearrangement resulting in a hydrogen and cation shift towards **7’** (Scheme 4). To test this hypothesis, this rearrangement was computed at the DFT level. A cyclic transition state (**TS**) was found between oxocarbenium **7** and **7’** which exists as a stabilized form including an intramolecular stabilizing interaction between the carbocation and the ester carbonyl group. However, in close agreement with Hess and Baldwin’s values found for the rearrangement of *cis*-1,3-pentadiene [24], this transition state was high in energy, with a difference of Gibbs free energy with oxocarbenium **7** (39.2 kcal/mol) and **7’** (35.8 kcal/mol) incompatible with the reaction conditions. We therefore ruled out this possibility and suggest that aldehyde **3** is the main intermediate in this transformation (Scheme 3).

**Figure 2:**
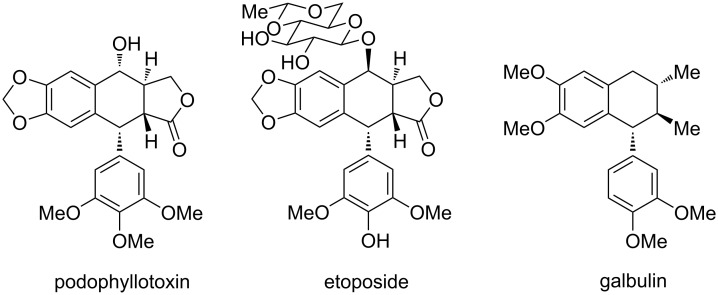
Examples of cyclolignan natural products [25–27].

Interestingly, the 1-aryltetraline product **6** holds a skeleton similar to that of cyclolignan natural products (Figure 2), which have often been targeted by total synthesis [18]. Some of them like podophyllotoxin [25] and the semisynthetic derivative etoposide [26] have demonstrated valuable anticancer properties [19–20]. Thus, to extend the scope of this tandem reaction sequence towards analogous skeletons, we explored the effect of various substituents on the aromatic cycles.

**Scheme 5:**
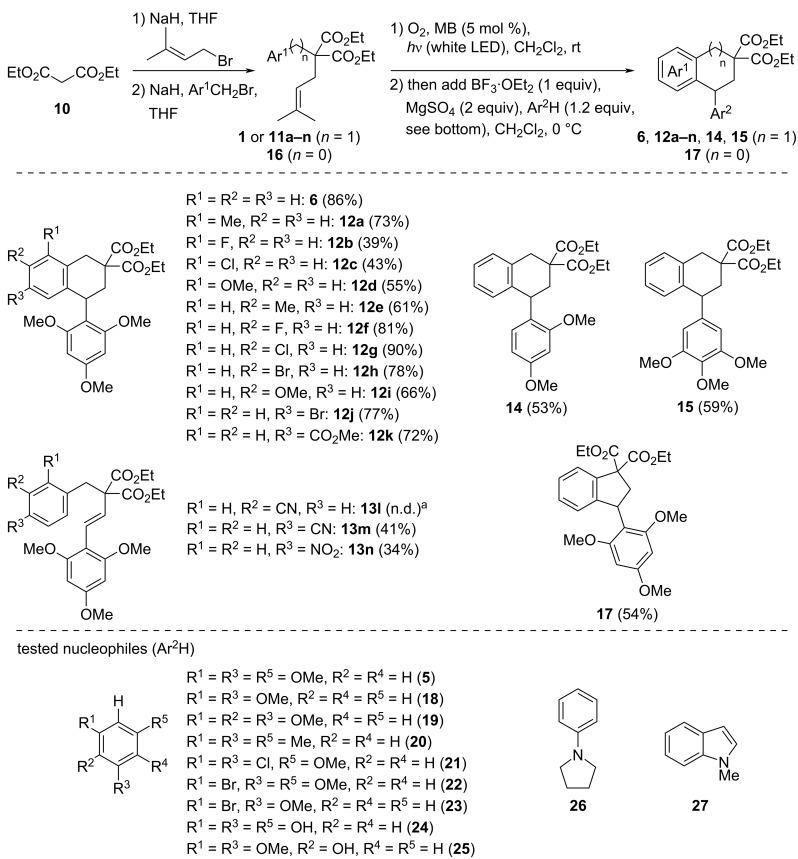
Scope of substrates and aromatic nucleophiles in the one-pot transformation. ^a^Not determined (mixture with unidentified products).

The successive prenylation and then diversity-oriented benzylation (*n* = 1 for substrates **11a–n**) of diethyl malonate (**10**) afforded a variety of new substrates to be submitted to the one-pot tandem transformation (Scheme 5). With only a few exceptions, the presence of *ortho* (R^1^), *meta* (R^2^), or *para* (R^3^) substituents on the benzyl moiety generally allowed the intramolecular Friedel–Crafts reaction, after the first intermolecular one in the presence of 1,3,5-trimethoxybenzene (**5**). In the *ortho* position, while a methyl group (product **12a**) showed little difference, the yields were more modest when a π-donor substituent (R^1^ = F, Cl, OMe) was present (**12b–d**). However, with the exception of the methyl group (**12e**), the yields were improved when a π-donating substituent in *meta* position (R^2^ = F, Cl, Br, OMe) was present (**12f–i**), as expected from the increased nucleophilicity of the carbon involved in the intramolecular Friedel–Crafts reaction. In the *para* position, R^3^ = Br and CO_2_Me were tested and showed good results, to give **12j** and **12k** with more than 70% yields. The success of the reaction with an ester substituent to give **12k** is surprising if we compare it with other electron-withdrawing groups like CN or NO_2_, which failed to give the corresponding cyclization products **12l**–**n** (not shown). Instead, olefin products **13l–n** arising from an elimination were obtained.

Furthermore, 1,3-dimethoxybenzene (**18**) and 1,2,3-trimethoxybenzene (**19**) were found to be good partners for this tandem transformation giving the expected products **14** and **15** in 53% and 59% respective yields when reacting with **1**. In particular, the aryl methoxy substituents of **15** are related to the podophyllotoxin structure [26]. However, other nucleophiles like **20–27** (Scheme 5) were unsuccessful, only leading to complex mixtures or occasionally to small amounts of **4** (<10%).

Finally, other homologous substrates were tested. While the tandem reaction with a phenylethyl substituent (*n* = 2, not shown) only led to a complex mixture of products, the transformation of phenylmalonate substrate **16** (*n* = 0, Ar^1^ = Ph) in the presence of nucleophile **5** allowed the formation of cyclized indanyl product **17** in a decent 54% yield.

## Conclusion

During this work, we demonstrated that the prenyl motif can be used as a surrogate of the aldehyde function when it is engaged in a tandem photooxygenation and Hock rearrangement, involving allylic hydroperoxide intermediates in an acidic medium. In the presence of aromatic nucleophiles, the aldehyde intermediate of the Hock rearrangement can be involved in tandem Friedel–Crafts reactions. Highly nucleophilic arenes like 1,3,5-trimethoxybenzene react easily under mild conditions and result in a stabilized benzylic cation in acidic conditions, allowing a second intramolecular Friedel–Crafts reaction involving the aryl substituent of the substrate. These reactions are favored by π-donor substituents, while highly electron-deficient substituents (CN, NO_2_) precluded the cyclization. Overall, this sequence led to valuable 1-aryltetralines structurally related to medicinally relevant cyclolignan natural products.

## Supporting Information

Crystallographic data of compound **6** were deposited in the Cambridge Crystallographic Data Center under the CCDC number 2301977.

File 1 Experimental procedures, compound characterizations, crystallographic data, DFT calculation, and spectra.

File 2 Crystallographic information file of compound **6**.

## Data Availability

All data that supports the findings of this study is available in the published article and/or the supporting information to this article.
