# Supplementary material for: Tandem Hock and Friedel–Crafts reactions allowing an expedient synthesis of a cyclolignan-type scaffold
Source: Beilstein J Org Chem. 2024 Jan 25;20:162–9. doi: 10.3762/bjoc.20.15 (PMC10825801; doi:10.3762/bjoc.20.15)

BEILSTEIN JOURNAL OF ORGANIC CHEMISTRY

Supporting Information

for

Tandem Hock and Friedel–Crafts reactions allowing an expedient synthesis of a cyclolignan-type scaffold

Viktoria A. Ikonnikova, Cristina Cheibas, Oscar Gayraud, Alexandra E. Bosnidou, Nicolas Casaretto, Gilles Frison and Bastien Nay

Beilstein J. Org. Chem. **2024,** *20,* 162–169. doi:10.3762/bjoc.20.15

Experimental procedures, compound characterizations, crystallographic data, DFT calculation, and spectra

License and Terms: This is a supporting information file under the terms of the Creative Commons Attribution License (https://creativecommons.org/licenses/by/4.0). Please note that the reuse, redistribution and reproduction in particular requires that the author(s) and source are credited and that individual graphics may be subject to special legal provisions.
The license is subject to the *Beilstein Journal of Organic Chemistry* terms and conditions: (https://www.beilstein-journals.org/bjoc/terms)

Content

General information **S3**

Experimental procedures and compound characterization **S4**

General procedure A for other (3-methylbut-2-enyl)malonate (S1) benzylation S4

General procedure B for the one-pot transformation S4

Diethyl (3-methylbut-2-enyl)malonate (**S1**) S4

Diethyl benzyl(3-methylbut-2-enyl)malonate (**1**) S5

Diethyl benzyl(2-oxoethyl)malonate (**3**) S6

Diethyl (1*H*)naphthalene-2,2-dicarboxylate (**4**) S6

3,3-Bis(ethoxycarbonyl)-1-(2,4,6-trimethoxyphenyl)-1,2,3,4-tetrahydronaphthalene (**6**) S7

Diethyl (2-methylbenzyl)(3-methylbut-2-enyl)malonate (**11a**) S7

Diethyl (2-fluorobenzyl)(3-methylbut-2-enyl)malonate (**11b**) S8

Diethyl (2-chlorobenzyl)(3-methylbut-2-enyl)malonate (**11c**) S8

Diethyl (2-methoxybenzyl)(3-methylbut-2-enyl)malonate (**11d**) S9

Diethyl (3-methylbenzyl)(3-methylbut-2-enyl)malonate (**11e**) S9

Diethyl (3-fluorobenzyl)(3-methylbut-2-enyl)malonate (**11f**) S10

Diethyl (3-chlorobenzyl)(3-methylbut-2-enyl)malonate (**11g**) S10

Diethyl (3-bromobenzyl)(3-methylbut-2-enyl)malonate (**11h**) S11

Diethyl (3-methoxybenzyl)(3-methylbut-2-enyl)malonate (**11i**) S11

Diethyl (4-bromobenzyl)(3-methylbut-2-enyl)malonate (**11j**) S11

Diethyl (4-methoxycarbonylbenzyl)(3-methylbut-2-enyl)malonate (**11k**) S12

Diethyl (3-cyanobenzyl)(3-methylbut-2-enyl)malonate (**11l**) S12

Diethyl (4-cyanobenzyl)(3-methylbut-2-enyl)malonate (**11m**) S13

Diethyl (4-nitrobenzyl)(3-methylbut-2-enyl)malonate (**11n**) S13

3,3-Bis(ethoxycarbonyl)-5-methyl-1-(2,4,6-trimethoxyphenyl)-1,2,3,4-tetrahydronaphthalene (**12a**) S14

3,3-Bis(ethoxycarbonyl)-5-fluoro-1-(2,4,6-trimethoxyphenyl)-1,2,3,4-tetrahydronaphthalene (**12b**) S14

3,3-Bis(ethoxycarbonyl)-5-chloro-1-(2,4,6-trimethoxyphenyl)-1,2,3,4-tetrahydronaphthalene (**12c**) S15

3,3-Bis(ethoxycarbonyl)-5-methoxy-1-(2,4,6-trimethoxyphenyl)-1,2,3,4-tetrahydronaphthalene (**12d**) S16

3,3-Bis(ethoxycarbonyl)-6-methyl-1-(2,4,6-trimethoxyphenyl)-1,2,3,4-tetrahydronaphthalene (**12e**) S16

3,3-Bis(ethoxycarbonyl)-6-fluoro-1-(2,4,6-trimethoxyphenyl)-1,2,3,4-tetrahydronaphthalene (**12f**) S17

3,3-Bis(ethoxycarbonyl)-6-chloro-1-(2,4,6-trimethoxyphenyl)-1,2,3,4-tetrahydronaphthalene (**12g**) S18

3,3-Bis(ethoxycarbonyl)-6-bromo-1-(2,4,6-trimethoxyphenyl)-1,2,3,4-tetrahydronaphthalene (**12h**) S18

3,3-Bis(ethoxycarbonyl)-6-methoxy-1-(2,4,6-trimethoxyphenyl)-1,2,3,4-tetrahydronaphthalene (**12i**) S19

3,3-Bis(ethoxycarbonyl)-7-bromo-1-(2,4,6-trimethoxyphenyl)-1,2,3,4-tetrahydronaphthalene (**12j**) S20

3,3-Bis(ethoxycarbonyl)-7-methoxycarbonyl-1-(2,4,6-trimethoxyphenyl)-1,2,3,4-tetrahydronaphthalene (**12k**) S20

2-[3,3-Bis(ethoxycarbonyl)-4-(4-cyanophenyl)-1-buten-1-yl]-1,3,5-trimethoxybenzene (**13m**) S21

2-[3,3-Bis(ethoxycarbonyl)-4-(4-nitrophenyl)-1-buten-1-yl]-1,3,5-trimethoxybenzene (**13n**) S22

3,3-Bis(ethoxycarbonyl)-1-(2,4-dimethoxyphenyl)-1,2,3,4-tetrahydronaphthalene (**14**) S22

3,3-Bis(ethoxycarbonyl)-1-(3,4,5-trimethoxyphenyl)-1,2,3,4-tetrahydronaphthalene (**15**) S23
Diethyl (3-methylbut-2-enyl)(phenyl)malonate (**16**) S23
3,3-Bis(ethoxycarbonyl)-1-(2,4,6-trimethoxyphenyl)-1,2-dihydroindene (**17**) S24
Crystal structure determination of 6 **S25**
Experimental S25
Crystal Data S25
DFT calculation **S31**
Cartesian coordinates of the optimized geometries and associated energies and free energy corrections obtained at the IEFPCM(CH_2Cl_2)-M06/6-311++G(2d,2p)//M06/6-31G(d,p) level of theory S31
Structure **7**.. S31
Structure **7’**.. S32
Transition state (**TS**).. S34
References **S36**
Copies of spectra **S37**
Diethyl (3-methylbut-2-enyl)malonate (**S1**) S37
Diethyl benzyl(3-methylbut-2-enyl)malonate (**1**) S38
Diethyl (1*H*)naphthalene-2,2-dicarboxylate (**4**) S39
3,3-Bis(ethoxycarbonyl)-1-(2,4,6-trimethoxyphenyl)-1,2,3,4-tetrahydronaphthalene (**6**) S40
Diethyl (2-methylbenzyl)(3-methylbut-2-enyl)malonate (**11a**) S41
Diethyl (2-fluorobenzyl)(3-methylbut-2-enyl)malonate (**11b**) S42
Diethyl (2-chlorobenzyl)(3-methylbut-2-enyl)malonate (**11c**) S43
Diethyl (2-methoxybenzyl)(3-methylbut-2-enyl)malonate (**11d**) S44
Diethyl (3-methylbenzyl)(3-methylbut-2-enyl)malonate (**11e**) S45
Diethyl (3-fluorobenzyl)(3-methylbut-2-enyl)malonate (**11f**) S46
Diethyl (3-chlorobenzyl)(3-methylbut-2-enyl)malonate (**11g**) S47
Diethyl (3-bromobenzyl)(3-methylbut-2-enyl)malonate (**11h**) S48
Diethyl (3-methoxybenzyl)(3-methylbut-2-enyl)malonate (**11i**) S49
Diethyl (4-bromobenzyl)(3-methylbut-2-enyl)malonate (**11j**) S50
Diethyl (4-methoxycarbonylbenzyl)(3-methylbut-2-enyl)malonate (**11k**) S51
Diethyl (3-cyanobenzyl)(3-methylbut-2-enyl)malonate (**11l**) S52
Diethyl (4-cyanobenzyl)(3-methylbut-2-enyl)malonate (**11m**) S53
Diethyl (4-nitrobenzyl)(3-methylbut-2-enyl)malonate (**11n**) S54
3,3-Bis(ethoxycarbonyl)-5-methyl-1-(2,4,6-trimethoxyphenyl)-1,2,3,4-tetrahydronaphthalene (**12a**) S55
3,3-Bis(ethoxycarbonyl)-5-fluoro-1-(2,4,6-trimethoxyphenyl)-1,2,3,4-tetrahydronaphthalene (**12b**) S56
3,3-Bis(ethoxycarbonyl)-5-chloro-1-(2,4,6-trimethoxyphenyl)-1,2,3,4-tetrahydronaphthalene (**12c**) S57
3,3-Bis(ethoxycarbonyl)-5-methoxy-1-(2,4,6-trimethoxyphenyl)-1,2,3,4-tetrahydronaphthalene (**12d**) S58
3,3-Bis(ethoxycarbonyl)-6-methyl-1-(2,4,6-trimethoxyphenyl)-1,2,3,4-tetrahydronaphthalene (**12e**) S59
3,3-Bis(ethoxycarbonyl)-6-fluoro-1-(2,4,6-trimethoxyphenyl)-1,2,3,4-tetrahydronaphthalene (**12f**) S60
3,3-Bis(ethoxycarbonyl)-6-chloro-1-(2,4,6-trimethoxyphenyl)-1,2,3,4-tetrahydronaphthalene (**12g**) S61
3,3-Bis(ethoxycarbonyl)-6-bromo-1-(2,4,6-trimethoxyphenyl)-1,2,3,4-tetrahydronaphthalene (**12h**) S62
3,3-Bis(ethoxycarbonyl)-6-methoxy-1-(2,4,6-trimethoxyphenyl)-1,2,3,4-tetrahydronaphthalene (**12i**) S63
3,3-Bis(ethoxycarbonyl)-7-bromo-1-(2,4,6-trimethoxyphenyl)-1,2,3,4-tetrahydronaphthalene (**12j**) S64
3,3-Bis(ethoxycarbonyl)-7-methoxy-1-(2,4,6-trimethoxyphenyl)-1,2,3,4-tetrahydronaphthalene (**12k**) S65

2-[3,3-Bis(ethoxycarbonyl)-4-(4-cyanophenyl)-1-buten-1-yl]-1,3,5-trimethoxybenzene (**13m**) S66
2-[3,3-Bis(ethoxycarbonyl)-4-(4-nitrophenyl)-1-buten-1-yl]-1,3,5-trimethoxybenzene (**13n**) S67
3,3-Bis(ethoxycarbonyl)-1-(2,4-dimethoxyphenyl)-1,2,3,4-tetrahydronaphthalene (**14**) S68
3,3-Bis(ethoxycarbonyl)-1-(3,4,5-trimethoxyphenyl)-1,2,3,4-tetrahydronaphthalene (**15**) S69
Diethyl (3-methylbut-2-enyl)(phenyl)malonate (**16**) S70
3,3-Bis(ethoxycarbonyl)-1-(2,4,6-trimethoxyphenyl)-1,2-dihydroindene (**17**) S71

General information

Reactions were conducted under a positive pressure of dry argon (except photooxygenation reactions that used oxygen). Anhydrous solvents were obtained from commercial sources. Commercially available chemicals were used without further purification.

Analytical TLC was performed with Merck silica gel plates, pre-coated with silica gel 60 F_{254} (0.2 mm). The visualization was effected by quenching of UV fluorescence (λ_{max} = 254 nm or 360 nm) and by staining with potassium permanganate or vanillin TLC stain solutions, followed by heating with a heat gun. Flash column chromatography was performed with silica gel (type 60, 40–63 μm).

Melting points were measured on a Stuart SMP3 melting point apparatus.

The NMR spectra were recorded at 298 K using a Bruker AVANCE 400 spectrometer. ^{1}H NMR spectra were recorded at 400 MHz and residual solvent peaks were used as an internal reference ($CDCl_3$ δ = 7.26 ppm). Data are reported as follows: chemical shift in ppm, apparent multiplicity (s = singlet, d = doublet, dd = doublet of doublet, t = triplet, m = multiplet or overlap of nonequivalent resonances), coupling constants, proton integration. Proton-decoupled ^{13}C NMR spectra (mentionned as ^{13}C{^{1}H}) were recorded at 100 MHz and residual solvent peaks were used as an internal reference ($CDCl_3$ δ = 77.0 ppm). Data are reported as follows: chemical shift in ppm (apparent multiplicity, coupling constants and integration when relevant).

Infrared spectra were recorded on a Perkin Elmer FT 1600 spectrometer with wavelengths in cm^{-1}.

High-resolution mass spectrometry (HRMS) experiments were performed on a Tims-TOF mass spectrometer (Bruker, France) using an electrospray ionization (ESI) source in the positive mode. All compounds were dissolved in CH_3CN then diluted by CH_3OH to obtained final concentration in the μM range. 10 μL were introduced in the instrument without separation. The elemental compositions of all ions were determined with the instrument software Data Analysis, the precision of mass measurement was less than 5 ppm.

Experimental procedures and compound characterization

General procedure A for other (3-methylbut-2-enyl)malonate (S1) benzylation

To a solution of NaH 60% in THF (0.5 M) under argon was added diethyl (3-methylbut-2-enyl)malonate **S1** (1 equiv) at 0 °C, and the reaction mixture was stirred at room temperature until complete dissolution. Then the benzyl bromide of interest (1.2 equiv) was added at 0 °C and the reaction mixture was stirred at room temperature for ≈16 hours while being monitored by TLC. After completion, the reaction mixture was quenched with a saturated solution of NH_4Cl. The mixture was extracted with EtOAc (3×), dried with Na_2SO_4, filtered and concentrated under reduced pressure. The crude mixture was purified by column chromatography to afford the pure product.

General procedure B for the one-pot transformation

To a solution of disubstituted diethyl malonate substrates (1 equiv) in DCM (0.1 M) under oxygen was added methylene blue (0.05 equiv), and the reaction mixture was irradiated with white light (LED) at room temperature for 2–4 hours, while being monitored by TLC. Then $MgSO_4$ (1 equiv), $BF_3{\cdot}OEt_2$ (1 equiv) and the nucleophile (1.2 equiv) were added at 0 °C and the reaction mixture was stirred vigorously under argon for 3 hours while being monitored by TLC. After evaporation, the crude mixture was purified by flash column chromatography on silica gel to afford the pure product.

Diethyl (3-methylbut-2-enyl)malonate (S1)

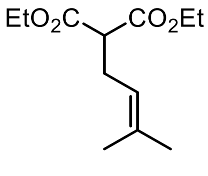

To a solution of NaH 60% (1.2 g, 30.0 mmol, 1.5 equiv) in THF (40 mL) under argon was slowly added diethyl malonate (6.1 mL, 40.0 mmol, 2 equiv) at 0 °C, and the reaction mixture was stirred at room temperature until complete dissolution. Then 3-methylbut-2-enyl bromide (technical 90%, 2.6 mL, 20.0 mmol, 1 equiv) was added at 0 °C and the reaction mixture was stirred vigorously at room temperature for ~16 hours while being monitored by TLC. After completion, the reaction mixture was quenched with a saturated solution of NH_4Cl. The mixture was extracted with EtOAc (3×), dried with Na_2SO_4, filtered and concentrated under reduced

pressure. The crude mixture was purified by column chromatography (petroleum ether or PE/Et_2O = 98:2 to 95:5) to afford pure product **S1** as a slightly colorless transparent oil (4.5 g, 98% yield).

The NMR data were in accordance with those reported in the literature.[1]

Rf = 0.25 (PE/Et_2O 9:1)

^{1}H NMR (400 MHz, $CDCl_3$): δ 5.09 – 5.04 (m, 1H), 4.18 (q, J = 7.1 Hz, 4H), 3.32 (t, J = 7.7 Hz, 1H), 2.60 – 2.56 (m, 2H), 1.68 – 1.67 (m, 3H), 1.63 (br s, 3H), 1.26 (t, J = 7.1 Hz, 6H).

^{13}C{^{1}H} NMR (100 MHz, $CDCl_3$): δ 169.3, 135.0, 119.7, 61.3, 52.2, 27.6, 25.7, 17.8, 14.1.

Diethyl benzyl(3-methylbut-2-enyl)malonate (1)

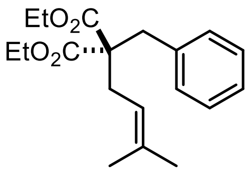

To a solution of NaH 60% (3 g, 77 mmol, 3.8 equiv) in THF (40 mL) under argon was slowly added commercial diethyl benzylmalonate (6.1 mL, 40.0 mmol, 2 equiv) at 0 °C, and the reaction mixture was stirred at room temperature until complete dissolution.[2] Then 3-methylbut-2-enyl bromide (technical 90%, 2.6 mL, 20.0 mmol, 1 equiv) was added at 0 °C and the reaction mixture was stirred vigorously at room temperature for ≈16 hours while being monitored by TLC. After completion, the reaction mixture was quenched with a saturated solution of NH_4Cl. The mixture was extracted with EtOAc (3×), dried with Na_2SO_4, filtered and concentrated under reduced pressure. The crude mixture was purified by column chromatography (petroleum ether or PE/AcOEt = 99:1 to 95:5) to afford pure product **1** as a slightly colorless transparent oil (6.04 g, 95% yield).

Rf = 0.43 (PE/EtOAc 95:5)

^{1}H NMR (400 MHz, $CDCl_3$): δ 7.26 – 7.20 (m, 3H), 7.08 – 7.06 (m, 2H), 5.13 – 5.08 (m, 1H), 4.19 – 4.14 (q, J = 7.2 Hz, 4H), 3.23 (s, 2H), 2.51 – 2.49 (m, 2H), 1.75 – 1.73 (m, 3H), 1.59 – 1.57 (m, 3H), 1.25 – 1.21 (m, 6H).

^{13}C{^{1}H} NMR (100 MHz, $CDCl_3$): δ 171.3, 136.4, 135.5, 130.1, 128.3, 127.0, 118.0, 61.3, 59.0, 38.0, 30.6, 26.2, 18.3, 14.2.

HRMS (ESI+): calculated for $C_{19}H_{27}O_4^+$ [MH^+]: 319.1904; found: 319.1904.

Diethyl benzyl(2-oxoethyl)malonate (3)

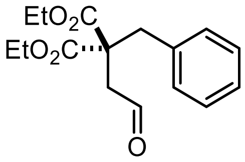

Compound **3** was occasionnally observed during our screening our conditions for the tandem reaction and in crude mixtures. For characterization, it was better prepared by ozonolysis through the following procedure:

A solution of diethyl 2-(3-methylbut-2-en-1-yl)-2-phenylmalonate (**1**) (100 mg, 0.3 mmol, 1 equiv) in 1:1 DCM-MeOH (0.4 M) was stirred under a flow of O_3 for 5 min at rt. After completion of the reaction monitored by TLC, the reaction mixture was quenched with a solution of Me_2S (50 µL, 0.68 mmol, 2.3 equiv) and extracted with ethyl actetate (3×). The combined organic layers were washed with a saturated aqueous solution of NaCl (2×) and NH_4Cl (1×), dried over Na_2SO_4, filtered and concentrated under reduced pressure. The crude mixture was purified by column chromatography over silica gel (PE:EA gradient 95:5 to 9:1), to afford compound **3** as a transparent oil (50 mg, 0.17 mmol, yield 57%).

^{1}H NMR (400 MHz, $CDCl_3$): δ 9.66 (t, J = 1.3 Hz, 1H), 7.29 – 7.23 (m, 3H), 7.05 – 7.02 (m, 2H), 4.23 (q, J = 7.2 Hz, 4H), 3.38 (s, 2H), 2.86 (d, J = 1.3 Hz, 2H), 1.26 (t, J = 7.2 Hz, 6H).

^{13}C NMR (400 MHz, $CDCl_3$): δ 199.4, 170.1, 135.7, 130.1, 128.7, 127.5, 62.1, 56.1, 46.0, 39.4, 14.1.

IR (ATR): $\tilde{\nu}$ 2937, 1722, 1183, 1092, 703 cm^{-1}.

Diethyl (1*H*)naphthalene-2,2-dicarboxylate (4)

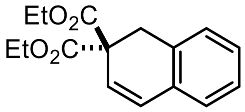

To a stirred solution of alkene **1** (64 mg, 0.23 mmol, 1 equiv) in DCM (2 mL) under oxygen was added MB (3.2 mg, 0.01 mmol, 0.05 equiv), and the reaction mixture was irradiated with white LED light at rt for 5 h while being monitored by TLC. Then, the oxygen atmosphere was purged with argon, and the reaction mixture was cooled down at 0 °C. $BF_3 \cdot OEt_2$ (25 µL, 0.2 mmol, 1 equiv) and $MgSO_4$ (49 mg 0.4 mmol, 2 equiv) were added. The reaction mixture was vigorously stirred under argon at 0 °C for 1 h, then at reflux overnight. After concentration under vacuum, the residue was purified by flash chromatography over silica gel (PE:Et_2O = 95:5) to afford compound **4** as transparent colorless oil (45 mg, 82%).

^{1}H NMR (400 MHz, CDCl$_3$): δ 7.18 – 7.15 (m, 3H), 7.08 – 7.05 (m, 1H), 6.64-6.61 (d, J = 9.6 Hz, 1H), 6.16-6.14 (d, J = 9.6 Hz, 1H), 4.22 – 4.14 (m, 4H), 3.41 (s, 2H), 1.24 – 1.21 (t, J = 7.1 Hz, 6H).

^{13}C NMR (400 MHz, CDCl$_3$): δ 170.2, 132.3, 131.7, 129.8, 128.2, 127.9, 127.1, 126.7, 124.7, 61.9, 55.0, 34.0, 14.1.

IR (ATR): $\tilde{\nu}$ 2982, 1730, 1225, 1185, 1104, 1047, 783, 700.

HRMS (ESI+): Calculated for $C_{16}H_{19}O_4^+$ [MH$^+$]: 275.1278; found: 275.1278.

3,3-Bis(ethoxycarbonyl)-1-(2,4,6-trimethoxyphenyl)-1,2,3,4-tetrahydronaphthalene (6)

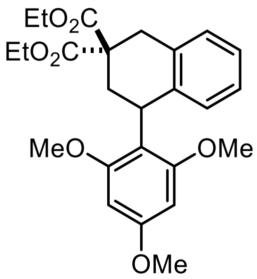

Synthesized by following general procedure B, from **1** (32 mg, 0.1 mmol) and 1,3,5-trimethoxybenzene (20 mg, 0.12 mmol). Purified over silica gel (PE:EA = 98:2 to 9:1) to afford product **6** as a white crystallin solid (38 mg, 0.086 mmol, 86% yield). Crystallisation performed from AcOEt for crystallography.

M.p. = 120-122 °C.

^{1}H NMR (400 MHz, CDCl$_3$): δ 7.07 (d, J = 7.3 Hz, 1H), 7.02 – 6.96 (m, 1H), 6.91 (t, J = 7.3 Hz, 1H), 6.70 (d, J = 7.3, 1H), 6.13 (br s, 2H), 4.81 (dd, J = 7.1, 11.3 Hz, 1H), 4.24 – 4.05 (m, 4H), 3.90 – 3.20 (overlapped signals, 11H: 3.80, s, 3H; 3.75, br s, 3H; 3.47, br s, 3H; 3.43, dd, J = 2.1, 15.7 Hz, 1H; 3.27, d, J = 15.7 Hz, 1H), 2.56 – 2.43 (m, 2H), 1.25 (t, J = 7.0 Hz, 3H), 1.17 (t, J = 7.0 Hz, 3H).

^{13}C{^{1}H} NMR (100 MHz, CDCl$_3$): δ 172.0, 170.7, 159.7 (2C), 159.1, 139.6, 133.2, 128.0, 126.8, 125.8, 124.7, 114.5, 91.3 (broad, 2C), 61.3, 61.0, 55.7 (broad, 2C), 55.2, 54.6, 35.2, 33.2, 31.5, 14.0, 13.9.

HRMS (ESI+): calculated for $C_{25}H_{31}O_7^+$ [MH$^+$]: 443.2064; found: 443.2065.

Diethyl (2-methylbenzyl)(3-methylbut-2-enyl)malonate (11a)

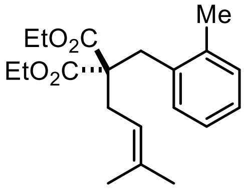

Synthesized through general procedure A from **S1** (457 mg, 2 mmol) and 2-methylbenzyl bromide (330 μL, 2.4 mmol) to afford compound **11a** as a colorless oil (517 mg, 78%).

^{1}H NMR (400 MHz, $CDCl_3$): δ 7.11 – 7.06 (m, 4H), 5.11 – 5.06 (m, 1H), 4.20 – 4.06 (m, 4H), 3.30 (s, 2H), 2.58 (br d, J = 7.2 Hz, 2H), 2.27 (s, 3H), 1.71 – 1.69 (m, 3H), 1.53 – 1.51 (m, 3H), 1.19 (t, J = 7.0 Hz, 6H).

^{13}C{^{1}H} NMR (100 MHz, $CDCl_3$): δ 171.6, 137.3, 135.3, 135.0, 130.4, 130.0, 126.6, 125.6, 118.2, 61.2, 58.6, 34.2, 31.4, 26.0, 19.8, 18.0, 13.9.

HRMS (ESI+): Calculated for $C_{20}H_{29}O_4^+$ [MH$^+$]: 333.2060; found: 333.2057.

Diethyl (2-fluorobenzyl)(3-methylbut-2-enyl)malonate (11b)

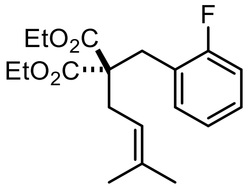

Synthesized through general procedure A from **S1** (228 mg, 1 mmol) and 2-fluorobenzyl iodide (237 mg, 1.2 mmol) to afford compound **11b** as a colorless oil (257 mg, 76% yield).

^{1}H NMR (400 MHz, $CDCl_3$): δ 7.22 – 7.14 (m, 1H), 7.14 – 7.07 (m, 1H), 7.04 – 6.92 (m, 2H), 5.12 (br t, J = 6.8 Hz, 1H), 4.21 – 4.10 (m, 4H), 3.29 (s, 2H), 2.49 (br d, J = 7.1 Hz, 2H), 1.71 (s, 3H), 1.56 (s, 3H), 1.22 (t, J = 7.1 Hz, 6H).

^{13}C{^{1}H} NMR (100 MHz, $CDCl_3$): δ 171.1, 161.6 (d, J = 246.4 Hz), 135.4, 132.3 (d, J = 4.4 Hz), 128.7 (d = 8.5 Hz), 123.7 (d, J = 3.6 Hz), 123.3 (d, J = 15.6 Hz), 117.8, 115.2 (d, J = 22.1 Hz), 61.2, 58.3, 31.2 (d, J = 2.0 Hz), 30.8, 26.0, 18.0, 14.0.

HRMS (ESI+): Calculated for $C_{19}H_{26}FO_4^+$ [MH$^+$]: 337.1810; found: 337.1807.

Diethyl (2-chlorobenzyl)(3-methylbut-2-enyl)malonate (11c)

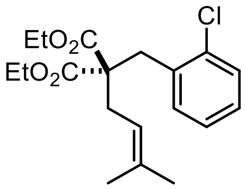

Synthesized through general procedure A from **S1** (228 mg, 1 mmol) and 2-chlorobenzyl iodide (303 mg, 1.2 mmol) to afford compound **11c** as a colorless oil (158 mg, 73% yield).

^{1}H NMR (400 MHz, CDCl$_3$): δ 7.33 – 7.29 (m, 1H), 7.24 – 7.20 (m, 1H), 7.16 – 7.11 (m, 2H), 5.16 – 5.10 (m, 1H), 4.21 – 4.08 (m, 4H), 3.44 (s, 2H), 2.60 – 2.55 (m, 2H), 1.72 – 1.69 (m, 3H), 1.57 – 1.55 (m, 3H), 1.19 (t, J = 7.1 Hz, 6H).

^{13}C{^{1}H} NMR (100 MHz, CDCl$_3$): δ 171.1, 135.3, 135.2, 134.7, 131.8, 129.5, 128.1, 126.4, 118.1, 61.3, 58.5, 34.8, 31.5, 26.0, 18.0, 13.9.

HRMS (ESI+): Calculated for $C_{19}H_{26}ClO_4^+$ [MH$^+$]: 353.1514; found: 353.1516.

Diethyl (2-methoxybenzyl)(3-methylbut-2-enyl)malonate (11d)

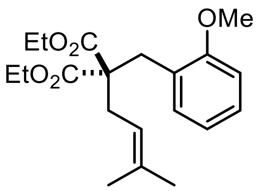

Synthesized through general procedure A from **S1** (228 mg, 1 mmol) and 2-methoxybenzyl bromide (241 mg, 1.2 mmol) to afford compound **11d** as a colorless oil (250 mg, 72% yield).

^{1}H NMR (400 MHz, CDCl$_3$): δ 7.18 (ddd, J = 1.8, 7.5, 8.2 Hz, 1H), 7.06 (dd, J = 1.8, 7.5 Hz, 1H), 6.85 – 6.78 (m, 2H), 5.21 – 5.15 (m, 1H), 4.21 – 4.08 (m, 4H), 3.73 (s, 3H), 3.32 (s, 2H), 2.47 – 2.43 (m, 2H), 1.72 – 1.70 (m, 3H), 1.56 – 1.54 (m, 3H), 1.22 (t, J = 7.2 Hz, 6H).

^{13}C{^{1}H} NMR (100 MHz, CDCl$_3$): δ 171.5, 158.1, 134.6, 131.8, 128.1, 124.9, 120.2, 118.6, 110.1, 60.9, 58.4, 55.0, 31.9, 30.8, 26.1, 18.0, 14.0.

HRMS (ESI+): Calculated for $C_{20}H_{29}O_5^+$ [MH$^+$]: 349.2010; found: 349.2009.

Diethyl (3-methylbenzyl)(3-methylbut-2-enyl)malonate (11e)

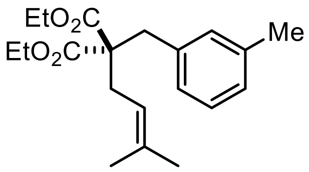

Synthesized through general procedure A from **S1** (457 mg, 2 mmol) and 3-methylbenzyl bromide (330 µL, 2.4 mmol) to afford compound **11e** as a colorless oil (664 mg, quantitative yield).

^{1}H NMR (400 MHz, CDCl$_3$): δ 7.14 – 7.10 (m, 1H), 7.03 – 6.99 (m, 1H), 6.88 – 6.84 (m, 2H), 5.12 – 5.07 (m, 1H), 4.17 (q, J = 7.0 Hz, 4H), 3.19 (s, 2H), 2.49 (br d, J = 7.0 Hz, 2H), 2.28 (s, 3H), 1.74 (br d, J = 1.3 Hz, 3H), 1.59 (br s, 3H), 1.24 (t, J = 7.0 Hz, 6H).

$^{13}C\{^1H\}$ NMR (100 MHz, $CDCl_3$): δ 171.2, 137.6, 136.1, 135.2, 130.8, 128.0, 127.5, 126.9, 118.0, 61.1, 58.8, 37.7, 30.4, 26.1, 21.4, 18.1, 14.0.

HRMS (ESI+): Calculated for $C_{20}H_{29}O_4^+$ [MH^+]: 333.2060; found: 333.2056.

Diethyl (3-fluorobenzyl)(3-methylbut-2-enyl)malonate (11f)

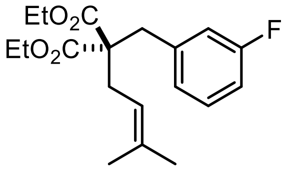

Synthesized through general procedure A from **S1** (228 mg, 1 mmol) and 3-fluorobenzyl bromide (160 μL, 1.2 mmol) to afford compound **11f** as a colorless oil (288 mg, 86% yield).

^1H NMR (400 MHz, $CDCl_3$): δ 7.22 – 7.17 (m, 1H), 6.92 – 6.87 (m, 1H), 6.85 – 6.83 (m, 1H), 6.80 – 6.77 (m, 1H), 5.09 – 5.04 (m, 1H), 4.17 (dq, J = 0.8, 7.1 Hz, 4H), 3.21 (s, 2H), 2.51 – 2.48 (m, 2H), 1.73 (m, 3H), 1.58 (m, 3H), 1.24 (t, J = 7.1 Hz, 6H).

$^{13}C\{^1H\}$ NMR (100 MHz, $CDCl_3$): δ 170.9, 162.5 (d, J = 245 Hz), 138.8 (d, J = 7.2 Hz), 135.6, 129.5 (d = 8.4 Hz), 125.6 (d, J = 2.9 Hz), 117.5, 116.9 (d, J = 21.4 Hz), 113.7 (d, J = 21.0 Hz), 61.3, 58.7, 37.5 (d, J = 1.4 Hz), 30.5, 26.0, 18.1, 14.0.

HRMS (ESI+): Calculated for $C_{19}H_{26}FO_4^+$ [MH^+]: 337.1810; found: 337.1805.

Diethyl (3-chlorobenzyl)(3-methylbut-2-enyl)malonate (11g)

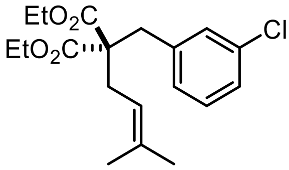

Synthesized through general procedure A from **S1** (228 mg, 1 mmol) and 3-chlorobenzyl bromide (160 μL, 1.2 mmol) to afford compound **11g** as a colorless oil (316 mg, 89% yield).

^1H NMR (400 MHz, $CDCl_3$): δ 7.21 – 7.14 (m, 2H), 7.07 – 7.06 (m, 1H), 6.96 – 6.94 (m, 1H), 5.08 – 5.03 (m, 1H), 4.17 (q, J = 7.1 Hz, 4H), 3.19 (s, 2H), 2.52 – 2.48 (m, 2H), 1.75 – 1.74 (m, 3H), 1.61 – 1.59 (m, 3H), 1.24 (t, J = 7.1 Hz, 6H).

$^{13}C\{^1H\}$ NMR (100 MHz, $CDCl_3$): δ 170.9, 138.3, 135.7, 133.9, 130.2, 129.4, 128.1, 127.0, 117.6, 61.3, 58.7, 37.5, 30.5, 26.0, 18.1, 14.0.

HRMS (ESI+): Calculated for $C_{19}H_{26}ClO_4^+$ [MH^+]: 353.1514; found: 353.1515.

Diethyl (3-bromobenzyl)(3-methylbut-2-enyl)malonate (11h)

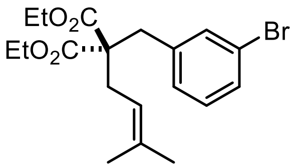

Synthesized through general procedure A from **S1** (228 mg, 1 mmol) and 3-bromobenzyl bromide (300 mg, 1.2 mmol) to afford compound **11h** as a colorless oil (332 mg, 84% yield).

^{1}H NMR (400 MHz, $CDCl_3$): δ 7.34 (ddd, J = 1.0, 2.1, 8.0 Hz, 1H), 7.22 (t, J = 1.8 Hz, 1H), 7.11 (t, J = 8.0 Hz, 1H), 7.01 – 6.98 (m, 1H), 5.07 – 5.01 (m, 1H), 4.17 (q, J = 7.1 Hz, 4H), 3.17 (s, 2H), 2.52 – 2.48 (m, 2H), 1.75 – 1.74 (m, 3H), 1.61 – 1.59 (m, 3H), 1.23 (t, J = 7.1 Hz, 6H).

^{13}C{^{1}H} NMR (100 MHz, $CDCl_3$): δ 170.9, 138.7, 135.7, 133.1, 129.9, 129.7, 128.6, 122.2, 117.6, 61.3, 58.7, 37.5, 30.5, 26.0, 18.1, 14.0.

HRMS (ESI+): Calculated for $C_{19}H_{26}BrO_4^+$ [MH^+]: 307.1009; found: 397.1010.

Diethyl (3-methoxybenzyl)(3-methylbut-2-enyl)malonate (11i)

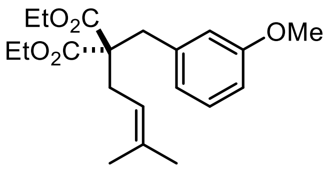

Synthesized through general procedure A from **S1** (114 mg, 0.5 mmol) and 3-methoxybenzyl chloride (87 µL, 0.6 mmol) to afford compound **11i** as a colorless oil (97 mg, 56% yield).

^{1}H NMR (400 MHz, $CDCl_3$): δ 7.17 – 7.13 (m, 1H), 6.76 – 6.73 (m, 1H), 6.67 – 6.62 (m, 2H), 5.12 – 5.07 (m, 1H), 4.17 (q, J = 7.2 Hz, 4H), 3.75 (s, 3H), 3.21 (s, 2H), 2.52 – 2.49 (m, 2H), 1.73 (m, 3H), 1.58 (m, 3H), 1.24 (t, J = 7.2 Hz, 6H).

^{13}C{^{1}H} NMR (100 MHz, $CDCl_3$): δ 171.1, 159.4, 137.8, 135.3, 129.1, 122.4, 117.8, 116.0, 112.0, 61.2, 58.8, 55.1, 37.8, 30.4, 26.1, 18.1, 14.0.

HRMS (ESI+): Calculated for $C_{20}H_{29}O_5^+$ [MH^+]: 349.2010; found: 349.2010.

Diethyl (4-bromobenzyl)(3-methylbut-2-enyl)malonate (11j)

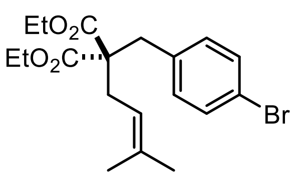

Synthesized through general procedure A from **S1** (228 mg, 1 mmol) and 4-bromobenzyl bromide (300 mg, 1.2 mmol) to afford compound **11j** as a colorless oil (301 mg, 76% yield).

^{1}H NMR (400 MHz, $CDCl_3$): δ 7.37 – 7.33 (m, 2H), 6.96 – 6.93 (m, 2H), 5.08 – 5.03 (m, 1H), 4.21 – 4.10 (m, 4H), 3.17 (s, 2H), 2.48 (d, J = 7.0 Hz, 2H), 1.74 – 1.71 (m, 3H), 1.58 (br s, 3H), 1.23 (dt, J = 0.4, 7.1 Hz, 6H).

^{13}C{^{1}H} NMR (100 MHz, $CDCl_3$): δ 170.9, 135.6, 135.3, 131.7, 131.3, 120.9, 117.6, 61.3, 58.7, 37.3, 30.6, 26.0, 18.1, 14.0.

HRMS (ESI+): Calculated for $C_{19}H_{26}BrO_4^+$ [MH^+]: 307.1009; found: 397.1016.

Diethyl (4-methoxycarbonylbenzyl)(3-methylbut-2-enyl)malonate (11k)

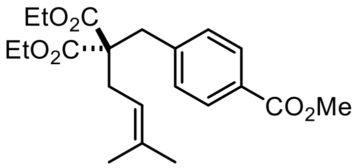

Synthesized through general procedure A from **S1** (210 mg, 0.92 mmol) and 4-(methoxycarbonyl)benzyl bromide (303 mg, 1.2 mmol) to afford compound **11k** as a colorless oil (282 mg, 90% yield).

^{1}H NMR (400 MHz, $CDCl_3$): δ 7.93 – 7.90 (m, 2H), 7.16 – 7.13 (m, 2H), 5.10 – 5.03 (m, 1H), 4.19 – 4.13 (m, 4H), 3.89 (s, 3H), 3.27 (s, 2H), 2.49 (d, J = 7.1 Hz, 2H), 1.75 – 1.73 (s, 3H), 1.59 – 1.56 (s, 3H), 1.22 (t, J = 7.2 Hz, 6H).

^{13}C{^{1}H} NMR (100 MHz, $CDCl_3$): δ 170.9, 167.0, 141.9, 135.7, 130.0, 129.4, 128.7, 117.5, 61.3, 58.7, 52.0, 37.9, 30.6, 26.0, 18.1, 14.0.

HRMS (ESI+): Calculated for $C_{21}H_{29}O_6^+$ [MH^+]: 377.1959; found: 377.1953.

Diethyl (3-cyanobenzyl)(3-methylbut-2-enyl)malonate (11l)

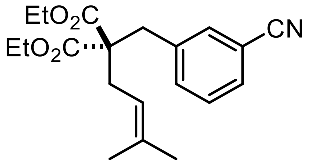

Synthesized through general procedure A from **S1** (228 mg, 1 mmol) and 3-cyanobenzyl bromide (235 mg, 1.2 mmol) to afford compound **11l** as a colorless oil (240 mg, 70% yield).

^{1}H NMR (400 MHz, $CDCl_3$): δ 7.52 (dt, J = 1.7, 7.1 Hz, 1H), 7.40 – 7.30 (m, 3H), 5.08 – 5.01 (m, 1H), 4.20 – 4.14 (two overlapped q, J = 7.1 Hz, 4H), 3.24 (s, 2H), 2.49 (d, J = 7.0 Hz, 2H), 1.76 – 1.74 (m, 3H), 1.60 – 1.58 (m, 3H), 1.23 (t, J = 7.2 Hz, 6H).

$^{13}C\{^1H\}$ **NMR (100 MHz, $CDCl_3$):** δ 170.7, 138.1, 136.1, 134.5, 133.7, 130.6, 128.0, 117.2, 112.3, 61.4, 58.7, 37.5, 30.8, 26.0, 18.1, 14.0.

HRMS (ESI+): Calculated for $C_{20}H_{26}NO_4^+$ [MH^+]: 344.1856; found: 344.1854.

Diethyl (4-cyanobenzyl)(3-methylbut-2-enyl)malonate (11m)

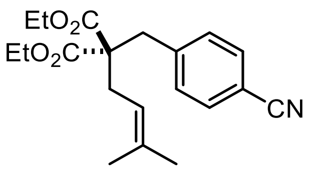

Synthesized through general procedure A from **S1** (228 mg, 1 mmol) and 4-cyanobenzyl bromide (235 mg, 1.2 mmol) to afford compound **11m** as a white solid (269 mg, 78% yield).

M.p. = 60-62 °C.

^1H **NMR (400 MHz, $CDCl_3$):** δ 7.53 (d, J = 8.2 Hz, 2H), 7.20 (d, J = 8.2 Hz, 2H), 5.07 – 5.01 (m, 1H), 4.20 – 4.08 (m, 4H), 3.26 (s, 2H), 2.48 (d, J = 7.1 Hz, 2H), 1.73 (s, 3H), 1.56 (s, 3H), 1.21 (t, J = 7.2 Hz, 6H).

$^{13}C\{^1H\}$ **NMR (100 MHz, $CDCl_3$):** δ 170.6, 142.2, 136.0, 131.8, 130.8, 118.7, 117.2, 110.8, 61.4, 58.6, 38.0, 30.9, 26.0, 18.0, 13.9.

HRMS (ESI+): Calculated for $C_{20}H_{26}NO_4^+$ [MH^+]: 344.1856; found: 344.1854.

Diethyl (4-nitrobenzyl)(3-methylbut-2-enyl)malonate (11n)

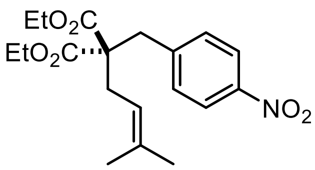

Synthesized through general procedure A from **S1** (228 mg, 1 mmol) and 4-nitrobenzyl bromide (259 mg, 1.2 mmol) to afford compound **11n** as a white solid (330 mg, 91% yield).

M.p. = 55-57 °C.

^1H **NMR (400 MHz, $CDCl_3$):** δ 8.12 – 8.09 (m, 2H), 7.27 – 7.24 (m, 2H), 5.09 – 5.02 (m, 1H), 4.21 – 4.13 (m, 4H), 3.30 (s, 2H), 2.50 (d, J = 7.1 Hz, 2H), 1.76 – 1.73 (s, 3H), 1.60 – 1.57 (s, 3H), 1.22 (t, J = 7.2 Hz, 6H).

$^{13}C\{^1H\}$ **NMR (100 MHz, $CDCl_3$):** δ 170.6, 147.0, 144.4 136.1, 130.9, 123.3, 117.2, 61.5, 58.7, 37.8, 30.9, 26.0, 18.2, 14.0.

HRMS (ESI+): Calculated for $C_{19}H_{26}NO_6^+$ [MH^+]: 364.1755; found: 364.1749.

3,3-Bis(ethoxycarbonyl)-5-methyl-1-(2,4,6-trimethoxyphenyl)-1,2,3,4-tetrahydronaphthalene (12a)

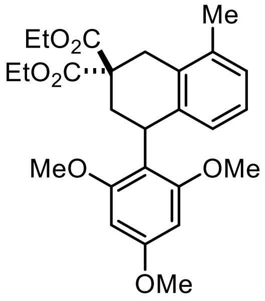

Synthesized by following general procedure B, from **11a** (50 mg, 0.15 mmol) and 1,3,5-trimethoxybenzene (**5**) (30 mg, 0.18 mmol). Purified over silica gel (PE:EA = 93:7 to 85:15) to afford product **12a** as a white solid (50 mg, 73% yield).

M.p. = 107.0-108.5 °C.

^{1}H NMR (400 MHz, $CDCl_3$): δ 6.90 – 6.86 (m, 1H), 6.83 (t, J = 7.5 Hz, 1H), 6.56 (br d, J = 7.5 Hz, 1H), 6.13 (br s, 2H), 4.78 (dd, J = 7.0, 11.0 Hz, 1H), 4.26 – 4.07 (m, 4H), 3.97 – 3.20 (overlapped signals, 10H: 3.80, s, 3H; 3.72, br s, 3H; 3.50, br s, 3H; 3.52, dd, J = 1.8, 16.2 Hz, 1H), 2.99 (d, J = 16.2 Hz, 1H), 2.56 – 2.45 (m, 2H), 2.31 (s, 3H), 1.27 (t, J = 7.1 Hz, 3H), 1.19 (t, J = 7.1 Hz, 3H).

^{13}C{^{1}H} NMR (100 MHz, $CDCl_3$): δ 172.3, 170.9, 159.7, 159.2 (broad), 139.4, 134.9, 131.7, 126.4, 125.2, 124.6, 114.7, 91.4 (broad), 61.3, 61.0, 55.8 (broad), 55.2, 54.6, 32.7, 31.9, 31.7, 19.9, 14.0, 13.9.

HRMS (ESI+): calculated for $C_{26}H_{33}O_7^+$ [MH^+]: 457.2221; found: 457.2221.

3,3-Bis(ethoxycarbonyl)-5-fluoro-1-(2,4,6-trimethoxyphenyl)-1,2,3,4-tetrahydronaphthalene (12b)

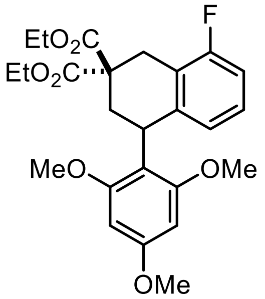

Synthesized by following general procedure B, from **11b** (51 mg, 0.15 mmol) and 1,3,5-trimethoxybenzene (**5**) (30 mg, 0.18 mmol). Purified over silica gel (PE:EA = 9:1) to afford product **12b** as a white solid (27 mg, 39% yield).

M.p. = 101-104 °C.

^{1}H NMR (400 MHz, $CDCl_3$): δ 6.92 – 6.85 (m, 1H), 6.78 – 6.71 (m, 1H), 6.50 (d, J = 7.8 Hz, 1H), 6.13 (br s, 2H), 4.74 (apparent t, J = 9.2 Hz, 1H), 4.25 – 4.08 (m, 4H), 4.00 – 3.18 (overlapped signals, 10H: 3.80, s, 3H; 3.78, br s, 3H; 3.70, d, J = 16.5 Hz, 1H; 3.45, br s, 3H), 2.97 (d, J = 16.5 Hz, 1H), 2.51 – 2.43 (m, 2H), 1.26 (t, J = 7.1 Hz, 3H), 1.19 (t, J = 7.1 Hz, 3H).

$^{13}C\{^1H\}$ NMR (100 MHz, $CDCl_3$): δ 171.8, 170.6, 160.2 (d, J = 243.2 Hz), 160.0, 159.1, 142.4 (d, J = 3.8 Hz), 126.2 (d, J = 8.9 Hz), 122.3 (d, J = 3.0 Hz), 121.1 (d, J = 16.8 Hz), 113.8, 111.1 (d, J = 21.5 Hz), 91.5 (broad), 61.5, 61.2, 55.7 (broad), 55.3, 53.8, 32.8, 31.5 (d, J = 2.4 Hz), 27.6 (d, J = 5.6 Hz), 14.1, 14.0.

HRMS (ESI+): calculated for $C_{25}H_{30}FO_7^+$ [MH^+]: 461.1970; found: 461.1961.

3,3-Bis(ethoxycarbonyl)-5-chloro-1-(2,4,6-trimethoxyphenyl)-1,2,3,4-tetrahydronaphthalene (12c)

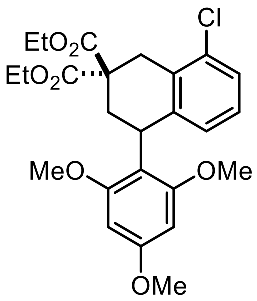

Synthesized by following general procedure B, from **11c** (53 mg, 0.15 mmol) and 1,3,5-trimethoxybenzene (**5**) (30 mg, 0.18 mmol). Purified over silica gel (PE:EA = 9:1 to 7:3) to afford product **12c** as a white solid (31 mg, 43% yield).

M.p. = 133.0-136.5 °C

^{1}H NMR (400 MHz, $CDCl_3$): δ 7.1 (dt, J = 7.8, 1.1 Hz, 1H), 6.86 (apparent t, J = 7.8 Hz, 1H), 6.63 (dt, J = 7.8, 1.1 Hz, 1H), 6.12 (br s, 2H), 4.75 (t, J = 9.1 Hz, 1H), 4.26 – 4.09 (m, 4H), 3.95 – 3.26 (overlapped signals, 10H: 3.81, d, J = 16.7 Hz, 1H; 3.80, s, 3H; 3.62, br s, 6H), 3.01 (d, J = 16.7 Hz, 1H), 2.50 – 2.43 (m, 2H), 1.27 (t, J = 7.1 Hz, 3H), 1.19 (t, J = 7.1 Hz, 3H).

$^{13}C\{^1H\}$ NMR (100 MHz, $CDCl_3$): δ 171.8, 170.6, 160.0, 159.1 (broad), 142.3, 133.2, 131.4, 126.4, 125.8, 125.4, 113.8, 91.2 (broad), 61.5, 61.2, 55.7 (broad), 55.3, 54.4, 32.7, 32.4, 31.9, 14.1, 13.9.

HRMS (ESI+): calculated for $C_{25}H_{30}ClO_7^+$ [MH^+]: 477.1675; found: 477.1674.

3,3-Bis(ethoxycarbonyl)-5-methoxy-1-(2,4,6-trimethoxyphenyl)-1,2,3,4-tetrahydronaphthalene (12d)

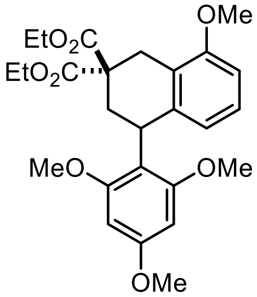

Synthesized by following general procedure B, from **11d** (52 mg, 0.15 mmol) and 1,3,5-trimethoxybenzene (**5**) (30 mg, 0.18 mmol). Purified over silica gel (PE:EA = 93:7 to 85:15) to afford product **12d** as a white solid (39 mg, 55% yield).

M.p. = 133.0-137.0 °C.

^{1}H NMR (400 MHz, $CDCl_3$): δ 6.89 (apparent t, J = 8.3 Hz, 1H), 6.57 (d, J = 8.3 Hz, 1H), 6.33 (d, J = 8.0 Hz, 1H), 6.13 (br s, 2H), 4.71 (dd, J = 6.4, 11.9 Hz, 1H), 4.26 – 4.07 (m, 4H), 3.93 – 3.59 (overlapped signals, 10H: 3.83, s, 1H; 3.80, s, 3H; 3.73, d, J = 16.7 Hz, 3H), 3.42 (br s, 3H), 2.85 (d, J = 16.7 Hz, 1H), 2.57 – 2.40 (m, 2H), 1.26 (t, J = 7.1 Hz, 3H), 1.20 (t, J = 7.1 Hz, 3H).

^{13}C{^{1}H} NMR (100 MHz, $CDCl_3$): δ 172.3, 170.9, 159.5, 159.2 (broad), 156.4, 140.9, 125.7, 122.3, 119.2, 114.4, 106.3, 92.1 (broad), 90.7 (broad), 61.3, 60.9, 55.7 (broad), 55.3, 55.2, 54.1, 32.6, 31.6, 28.5, 14.0, 13.9.

HRMS (ESI+): calculated for $C_{26}H_{33}O_8^+$ [MH^+]: 473.2170; found: 473.2163.

3,3-Bis(ethoxycarbonyl)-6-methyl-1-(2,4,6-trimethoxyphenyl)-1,2,3,4-tetrahydronaphthalene (12e)

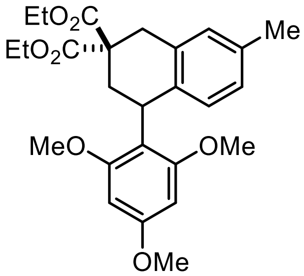

Synthesized by following general procedure B, from **11e** (50 mg, 0.15 mmol) and 1,3,5-trimethoxybenzene (**5**) (30 mg, 0.18 mmol). Purified over silica gel (PE:EA = 93:7 to 85:15) to afford product **12e** as a white solid (42 mg, 61% yield).

M.p. = 106-108 °C.

^{1}H NMR (400 MHz, $CDCl_3$): δ 6.88 (s, 1H), 6.75 (d, J = 7.9 Hz, 1H), 6.57 (d, J = 7.9 Hz, 1H), 6.13 (br s, 2H), 4.74 (apparent t, J = 9.4 Hz, 1H), 4.25 – 4.05 (m, 4H), 3.97 – 3.17 (overlapped signals, 11H: 3.80, s, 3H; 3.75, br s, 3H; 3.50, br s, 3H; 3.39, d, J = 15.9 Hz, 1H; 3.22, d, J = 15.9 Hz, 1H), 2.52 – 2.45 (m, 2H), 2.23 (s, 3H), 1.25 (t, J = 7.2 Hz, 3H), 1.18 (t, J = 7.2 Hz, 3H).

^{13}C{^{1}H} NMR (100 MHz, $CDCl_3$): δ 172.1, 170.8, 159.7, 159.2 (broad), 136.4, 133.9, 132.9, 128.6, 126.8, 126.7, 114.6, 91.4 (broad), 61.3, 61.0, 55.8 (broad), 55.2, 54.7, 35.2, 33.3, 31.2, 20.9, 14.0, 13.9.

HRMS (ESI+): calculated for $C_{26}H_{33}O_7^+$ [MH$^+$]: 457.2221; found: 457.2220.

3,3-Bis(ethoxycarbonyl)-6-fluoro-1-(2,4,6-trimethoxyphenyl)-1,2,3,4-tetrahydronaphthalene (12f)

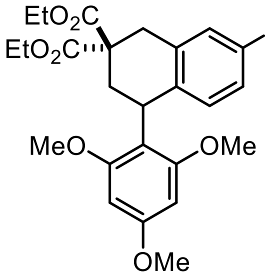

Synthesized by following general procedure B, from **11f** (51 mg, 0.15 mmol) and 1,3,5-trimethoxybenzene (**5**) (30 mg, 0.18 mmol). Purified over silica gel (PE:EA = 9:1 to 7:3) to afford product **12f** as a white solid (56 mg, 81% yield).

M.p. = 126-128 °C

^{1}H NMR (400 MHz, $CDCl_3$): δ 6.78 (dd, J = 2.2, 9.4 Hz, 1H), 6.68 – 6.58 (m, 2H), 6.13 (br s, 2H), 4.74 (dd, J = 6.7, 11.2 Hz, 1H), 4.25 – 4.07 (m, 4H), 4.00 – 3.15 (overlapped signals, 11H: 3.80, s, 3H; 3.62, br s, 6H; 3.41, dd, J = 2.5, 16.1 Hz, 1H; 3.24, d, J = 16.1 Hz, 1H), 2.52 (ddd, J = 2.4, 6.7, 13.0 Hz, 1H), 2.43 (dd, J = 11.4, 12.4 Hz, 1H), 1.25 (t, J = 7.1 Hz, 3H), 1.18 (t, J = 7.1 Hz, 3H).

^{13}C{^{1}H} NMR (100 MHz, $CDCl_3$): δ 171.8, 170.6, 160.3 (d, J = 242 Hz), 159.9, 159.1, 135.3 – 135.2 (2 overlapped d), 128.3 (d, J = 8.2 Hz), 114.2 (d, J = 10.2 Hz), 114.1 (d, J = 10.2 Hz), 112.9 (d, J = 21.2 Hz), 91.1 (broad), 61.5, 61.2, 55.8 (broad), 55.3, 54.6, 35.3, 33.2, 31.1, 14.1, 14.0.

HRMS (ESI+): calculated for $C_{25}H_{30}FO_7^+$ [MH$^+$]: 461.1970; found: 461.1963.

3,3-Bis(ethoxycarbonyl)-6-chloro-1-(2,4,6-trimethoxyphenyl)-1,2,3,4-tetrahydronaphthalene (12g)

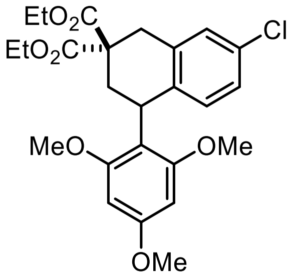

Synthesized by following general procedure B, from **11g** (53 mg, 0.15 mmol) and 1,3,5-trimethoxybenzene (**5**) (30 mg, 0.18 mmol). Purified over silica gel (PE:EA = 9:1 to 7:3) to afford product **12g** as a white solid (57 mg, 90% yield).

M.p. = 124.0-126.0 °C.

^{1}H NMR (400 MHz, $CDCl_3$): δ 7.07 (d, J = 2.2 Hz, 1H), 6.89 (dd, J = 2.2, 8.2 Hz, 1H), 6.64 (dd, J = 1.0, 8.2 Hz, 1H), 6.12 (br s, 2H), 4.74 (dd, J = 6.6, 11.5 Hz, 1H), 4.25 – 4.07 (m, 4H), 3.95 – 3.17 (overlapped signals, 11H: 3.80, s, 3H; 3.72, br s, 3H; 3.52, br s, 3H; 3.40, dd, J = 2.5, 16.1 Hz, 1H; 3.22, d, J = 16.1 Hz, 1H), 2.52 (ddd, J = 2.4, 6.7, 13.0 Hz, 1H), 2.42 (dd, J = 11.6, 13.0 Hz, 1H), 1.25 (t, J = 7.1 Hz, 3H), 1.19 (t, J = 7.1 Hz, 3H).

^{13}C{^{1}H} NMR (100 MHz, $CDCl_3$): δ 171.6, 170.5, 159.9, 158.9 (broad), 138.3, 135.2, 130.0, 128.3, 127.6, 126.0, 113.6, 91.1 (broad), 61.4, 61.1, 55.6 (broad), 55.2, 54.4, 34.9, 33.0, 31.1, 14.0, 13.9.

HRMS (ESI+): calculated for $C_{25}H_{30}ClO_7^+$ [MH^+]: 477.1675; found: 477.1671.

3,3-Bis(ethoxycarbonyl)-6-bromo-1-(2,4,6-trimethoxyphenyl)-1,2,3,4-tetrahydronaphthalene (12h)

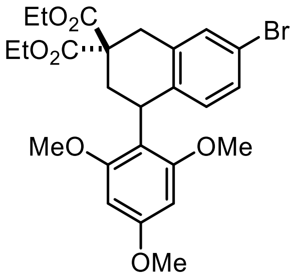

Synthesized by following general procedure B, from **11h** (84 mg, 0.21 mmol) and 1,3,5-trimethoxybenzene (**5**) (42 mg, 0.25 mmol). Purified over silica gel (PE:EA = 9:1 to 7:3) to afford product **12h** as a white solid (86 mg, 78% yield).

M.p. = 134-137 °C.

^{1}H NMR (400 MHz, $CDCl_3$): δ 7.22 (d, J = 2.0 Hz, 1H), 7.04 (dd, J = 2.0, 8.2 Hz, 1H), 6.58 (dd, J = 1.0, 8.2 Hz, 1H), 6.12 (br s, 2H), 4.71 (dd, J = 6.6, 11.5 Hz, 1H), 4.25 – 4.07 (m, 4H), 3.95 – 3.17 (overlapped signals, 11H: 3.80, s, 3H; 3.76, br s, 3H; 3.50, br s, 3H; 3.39, dd, J = 2.3, 16.0 Hz, 1H; 3.22, dq, J = 16.0, 1.1 Hz, 1H), 2.51 (ddd, J = 2.5, 6.6, 13.1 Hz, 1H), 2.41 (dd, J = 11.6, 13.1 Hz, 1H), 1.25 (t, J = 7.1 Hz, 3H), 1.19 (t, J = 7.1 Hz, 3H).

^{13}C{^{1}H} NMR (100 MHz, $CDCl_3$): δ 171.6, 170.5, 160.0, 159.0 (broad), 138.9, 135.6, 130.6, 128.9, 128.7, 118.2, 113.6, 91.2 (broad), 61.5, 61.2, 55.7 (broad), 55.2, 54.4, 34.9, 33.0, 31.2, 14.0, 13.9.

HRMS (ESI+): calculated for $C_{25}H_{30}BrO_7^+$ [MH^+]: 521.1169; found: 521.1165.

3,3-Bis(ethoxycarbonyl)-6-methoxy-1-(2,4,6-trimethoxyphenyl)-1,2,3,4-tetrahydronaphthalene (12i)

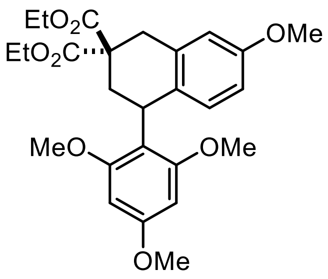

Synthesized by following general procedure B, from **11i** (35 mg, 0.1 mmol) and 1,3,5-trimethoxybenzene (**5**) (20 mg, 0.12 mmol). Purified over silica gel (PE:EA = 9:1 to 8:2) to afford product **12i** as a yellowish solid (31 mg, 66% yield).

M.p. = 95.0-96.5 °C.

^{1}H NMR (400 MHz, $CDCl_3$): δ 6.62 (d, J = 2.6 Hz, 1H), 6.61 (dd, J = 0.9, 8.6 Hz, 1H), 6.51 (dd, J = 2.6, 8.6 Hz, 1H), 6.13 (br s, 2H), 4.73 (dd, J = 7.2, 11.0 Hz, 1H), 4.25 – 4.07 (m, 4H), 3.90 – 3.33 (overlapped signals, 14H: 3.80, s, 3H; 3.74, s, 3H, 3.71, br s, 3H; 3.47, br s, 3H; 3.40, dd, J = 1.7, 15.8 Hz, 1H; 3.22, d, J = 15.8 Hz, 1H), 2.51 (ddd, J = 2.1, 7.0, 13.0 Hz, 1H), 2.46 (dd, J = 11.4, 13.0 Hz, 1H), 1.25 (t, J = 7.1 Hz, 3H), 1.19 (t, J = 7.1 Hz, 3H).

^{13}C{^{1}H} NMR (100 MHz, $CDCl_3$): δ 172.0, 170.7, 159.7, 159.2 (broad), 156.7, 134.3, 131.8, 127.8, 114.6, 112.6, 112.1, 91.4 (broad), 61.4, 61.0, 55.9 (broad), 55.2, 55.0, 54.7, 35.5, 33.3, 30.8, 14.0, 13.9.

HRMS (ESI+): calculated for $C_{26}H_{33}O_8^+$ [MH^+]: 473.2170; found: 473.2173.

3,3-Bis(ethoxycarbonyl)-7-bromo-1-(2,4,6-trimethoxyphenyl)-1,2,3,4-tetrahydronaphthalene (12j)

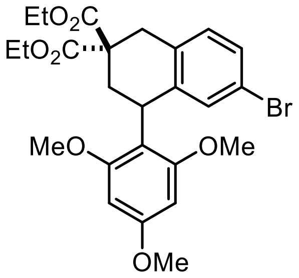

Synthesized by following general procedure B, from **11j** (60 mg, 0.15 mmol) and 1,3,5-trimethoxybenzene (**5**) (30 mg, 0.18 mmol). Purified over silica gel (PE:EA = 85:15 to 80:20) to afford product **12j** as a white solid (60 mg, 77% yield).

M.p. = 131-134 °C.

^{1}H NMR (400 MHz, $CDCl_3$): δ 7.12 (ddd, J = 0.9, 2.1, 8.1 Hz, 1H), 6.95 (d, J = 8.1 Hz, 1H), 6.84 (dd, J = 1.1, 2.0 Hz, 1H), 6.13 (br s, 2H), 4.75 (dd, J = 6.9, 11.6 Hz, 1H), 4.24 – 4.06 (m, 4H), 3.95 – 3.30 (overlapped signals, 10H: 3.81, s, 3H; 3.73, br s, 3H; 3.54, br s, 3H; 3.39, dd, J = 2.4, 16.0 Hz, 1H; 3.20 – 3.14, m, 1H), 2.51 (ddd, J = 2.6, 6.6, 13.1 Hz, 1H), 2.41 (dd, J = 11.7, 13.1 Hz, 1H), 1.25 (t, J = 7.1 Hz, 3H), 1.19 (t, J = 7.1 Hz, 3H).

^{13}C{^{1}H} NMR (100 MHz, $CDCl_3$): δ 171.7, 170.5, 160.0, 158.9 (broad), 142.2, 132.4, 129.7, 129.6, 127.9, 119.7, 113.4, 91.3 (broad), 61.5, 61.2, 55.7 (broad), 55.2, 54.4, 34.7, 33.0, 31.5, 14.0, 13.9.

HRMS (ESI+): calculated for $C_{25}H_{30}BrO_7^+$ [MH^+]: 521.1169; found: 521.1162.

3,3-Bis(ethoxycarbonyl)-7-methoxycarbonyl-1-(2,4,6-trimethoxyphenyl)-1,2,3,4-tetrahydronaphthalene (12k)

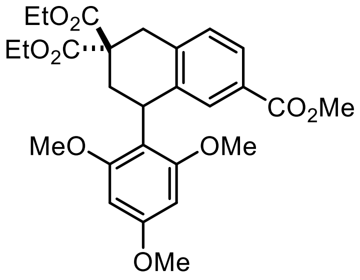

Synthesized by following general procedure B, from **11k** (57 mg, 0.15 mmol) and 1,3,5-trimethoxybenzene (**5**) (30 mg, 0.18 mmol). Purified over silica gel (PE:EA = 85:15 to 75:25) to afford product **12k** as a yellowish solid (54 mg, 72% yield).

M.p. = 150-152 °C.

^{1}H NMR (400 MHz, CDCl$_3$): δ 7.67 (ddd, J = 0.8, 1.8, 8.0 Hz, 1H), 7.41 – 7.39 (m, 1H), 7.15 (d, J = 8.0 Hz, 1H), 6.12 (br s, 2H), 4.79 (dd, J = 6.8, 11.6 Hz, 1H), 4.22 – 4.04 (m, 4H), 3.93 – 3.18 (overlapped signals, 14H: 3.80, s, 3H; 3.77, s, 3H; 3.62, br s, 6H; 3.48, dd, J = 2.6, 16.3 Hz, 1H; 3.30 – 3.24, m, 1H), 2.54 (ddd, J = 2.5, 6.6, 13.0 Hz, 1H), 2.40 (dd, J = 11.8, 13.0 Hz, 1H), 1.25 (t, J = 7.0 Hz, 3H), 1.16 (t, J = 7.0 Hz, 3H).

^{13}C{^{1}H} NMR (100 MHz, CDCl$_3$): δ 171.6, 170.4, 167.4, 159.9, 158.9, 140.2, 139.0, 128.2, 128.1, 127.8, 125.9, 113.6, 91.3 (broad), 61.4, 61.1, 55.6 (broad), 55.1, 54.4, 51.6, 35.4, 33.3, 31.5, 14.0, 13.9.

HRMS (ESI+): calculated for $C_{27}H_{33}O_9^+$ [MH$^+$]: 501.2119; found: 501.2123.

2-[3,3-Bis(ethoxycarbonyl)-4-(4-cyanophenyl)-1-buten-1-yl]-1,3,5-trimethoxybenzene (13m)

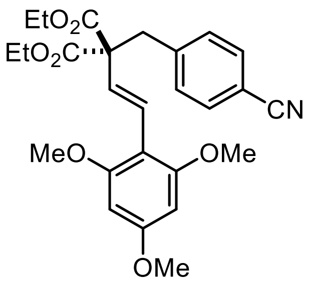

Synthesized by following general procedure B, from **11m** (52 mg, 0.15 mmol) and 1,3,5-trimethoxybenzene (**5**) (30 mg, 0.18 mmol). Purified over silica gel (PE:EA = 85:15 to 80:20) to afford product **13m** as an orange solid (29 mg, 41% yield).

M.p. = 112-115 °C.

^{1}H NMR (400 MHz, CDCl$_3$): δ 7.52 – 7.48 (m, 2H), 7.34 – 7.30 (m, 2H), 6.81 (d, J = 17.1 Hz, 1H), 6.72 (d, J = 17.1 Hz, 1H), 6.11 (s, 2H), 4.28 – 4.13 (m, 4H), 3.81 (s, 3H), 3.77 (s, 6H), 3.50 (s, 2H), 1.24 (t, J = 7.1 Hz, 6H).

^{13}C{^{1}H} NMR (100 MHz, CDCl$_3$): δ 170.2, 160.5, 159.4, 142.3, 131.5, 131.3, 126.7, 122.6, 119.0, 110.5, 106.9, 90.6, 61.6, 61.5, 55.6, 55.3, 42.5, 14.0.

HRMS (ESI+): calculated for $C_{26}H_{30}NO_7^+$ [MH$^+$]: 468.2017; found: 468.2016.

2-[3,3-Bis(ethoxycarbonyl)-4-(4-nitrophenyl)-1-buten-1-yl]-1,3,5-trimethoxybenzene (13n)

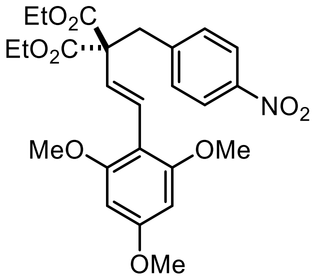

Synthesized by following general procedure B, from **11n** (55 mg, 0.15 mmol) and 1,3,5-trimethoxybenzene (30 mg, 0.18 mmol). Purified over silica gel (PE:EA = 85:15 to 80:20) to afford product **13n** as a yellow solid (25 mg, 34% yield).

M.p. = 119.0-120.5 °C.

^{1}H NMR (400 MHz, $CDCl_3$): δ 8.09 – 8.05 (m, 2H), 7.40 – 7.37 (m, 2H), 6.82 (d, J = 16.8 Hz, 1H), 6.74 (d, J = 16.8, 1H), 6.12 (s, 2H), 4.29 – 4.15 (m, 4H), 3.82 (s, 3H), 3.78 (s, 6H), 3.55 (s, 2H), 1.25 (t, J = 7.1 Hz, 6H).

^{13}C{^{1}H} NMR (100 MHz, $CDCl_3$): δ 170.2, 160.6, 159.4, 146.9, 144.5, 131.4, 126.6, 122.9, 122.7, 106.9, 90.6, 61.7, 61.5, 55.6, 55.3, 42.3, 14.0.

HRMS (ESI+): calculated for $C_{25}H_{30}NO_9^+$ [MH$^+$]: 488.1915; found: 488.1909.

3,3-Bis(ethoxycarbonyl)-1-(2,4-dimethoxyphenyl)-1,2,3,4-tetrahydronaphthalene (14)

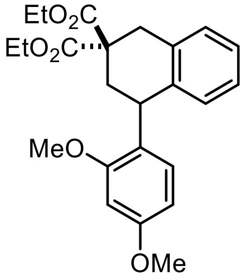

Synthesized by following general procedure B, from **1** (32 mg, 0.1 mmol) and 1,3-dimethoxybenzene (**18**) (16 µL, 0.12 mmol). Purified over silica gel (PE:EA = 95:5 to 90:10) to afford product **14** as an yellowish oil (22 mg, 53% yield).

^{1}H NMR (400 MHz, $CDCl_3$): δ 7.16 – 7.12 (m, 1H), 7.11 – 7.06 (m, 1H), 7.04 – 6.99 (m, 1H), 6.85 (d, J = 8.4 Hz, 1H), 6.79 – 6.77 (m, 1H), 6.48 (d, J = 2.4 Hz, 1H), 6.41 (dd, J = 8.4, 2.4 Hz, 1H), 4.53 (dd, J = 11.0, 6.2

Hz, 1H), 4.26 – 4.05 (m, 4H), 3.79 (s, 3H), 3.75 (s, 3H), 3.43 (dd, J = 16.0, 2.0 Hz, 1H), 3.30 (d, J = 16.0 Hz, 1H), 2.70 (ddd, J = 13.4, 6.1, 2.2 Hz, 1H), 2.25 (dd, J = 13.4, 11.0 Hz, 1H), 1.23 – 1.18 (m, 6H).

$^{13}C\{^1H\}$ NMR (100 MHz, $CDCl_3$): δ 171.7, 170.8, 159.4, 158.4, 138.5, 134.1, 130.1, 128.8, 128.5, 126.4, 126.1, 125.8, 104.3, 98.6, 61.5, 61.3, 55.5, 55.3, 54.1, 36.0, 35.8, 35.2, 14.0.

HRMS (ESI+): calculated for $C_{24}H_{29}O_6^+$ [MH^+]: 413.1959; found: 413.1959.

3,3-Bis(ethoxycarbonyl)-1-(3,4,5-trimethoxyphenyl)-1,2,3,4-tetrahydronaphthalene (15)

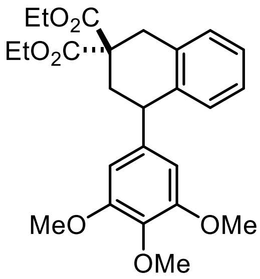

Synthesized by following general procedure B, from **1** (96 mg, 0.3 mmol) and 1,2,3-trimethoxybenzene (61 mg, 0.36 mmol). Purified over silica gel (PE:EA = 9:1) to afford product **15** as a colorless oil (78 mg, 59% yield).

^1H NMR (400 MHz, $CDCl_3$): δ 7.14 (d, J = 7.5 Hz, 1H), 7.10 – 7.05 (m, 1H), 7.02 – 7.97 (m, 1H), 6.78 – 6.75 (m, 1H), 6.73 (d, J = 8.6 Hz, 1H), 6.60 (d, J = 8.6 Hz, 1H), 4.43 (dd, J = 6.4, 11.4 Hz, 1H), 4.20 – 4.12 (m, 4H), 3.87 (s, 3H), 3.83 (s, 3H), 3.61 (br s, 3H), 3.47 (dd, J = 16.2, 2.2 Hz, 1H), 3.30 (d, J = 16.2 Hz, 1H), 2.70 (ddd, J = 13.2, 6.2, 2.4 Hz, 1H), 2.27 (dd, J = 13.2, 11.6 Hz, 1H), 1.24 (t, J = 7.1 Hz, 3H), 1.19 (t, J = 7.1 Hz, 3H).

$^{13}C\{^1H\}$ NMR (100 MHz, $CDCl_3$): δ 171.6, 170.5, 152.3, 152.1, 142.3, 138.5, 133.9, 131.8, 128.6, 128.5, 126.1, 125.8, 124.0, 107.2, 61.4, 61.2, 60.6, 60.5, 55.8, 54.1, 37.3 (broad), 36.5, 35.1, 13.9 (2 peaks).

HRMS (ESI+): calculated for $C_{25}H_{31}O_7^+$ [MH^+]: 443.2064; found: 443.2061.

Diethyl (3-methylbut-2-enyl)(phenyl)malonate (16)

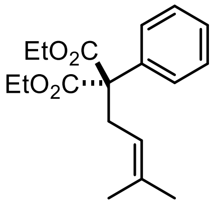

To a solution of NaH 60% (240 mg, 6 mmol, 1.2 equiv) in THF (10 mL) under argon was slowly added diethyl phenylmalonate (1.18 g, 5 mmol) at 0 °C, and the reaction mixture was stirred at room temperature until complete dissolution. Then 3-methylbut-2-enyl bromide (technical 90%, 780 µL, 6 mmol, 1.2 equiv) was added

dropwise at 0 °C and the reaction mixture was stirred vigorously at room temperature for 2.5 hours while being monitored by TLC. After completion, the reaction mixture was quenched with a saturated solution of NH_4Cl. The mixture was extracted with EtOAc (3×), dried with Na_2SO_4, filtered and concentrated under reduced pressure. The crude mixture was purified by column chromatography (PE/Et_2O = 98:2 to 90:10) to afford pure product **16** (1.29 g, 85% yield) as a colorless liquid.

^{1}H NMR (400 MHz, $CDCl_3$): δ 7.44 – 7.40 (m, 2H), 7.35 – 7.27 (m, 3H), 5.08 – 5.03 (m, 1H), 4.26 – 4.14 (m, 4H), 3.04 – 3.01 (m, 2H), 1.64 – 1.62 (m, 3H), 1.52 – 15.0 (m, 3H), 1.23 (t, J = 7.1 Hz, 6H).

^{13}C{^{1}H} NMR (100 MHz, $CDCl_3$): δ 170.6, 136.9, 135.3, 128.3, 128.0, 127.4, 118.3, 62.6, 61.4, 34.4, 25.9, 17.8, 14.0.

HRMS (ESI+): Calculated for $C_{18}H_{25}O_4^+$ [MH^+]: 305.1747; found: 305.1748.

3,3-Bis(ethoxycarbonyl)-1-(2,4,6-trimethoxyphenyl)-1,2-dihydroindene (17)

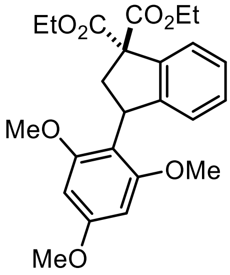

Synthesized by following general procedure B, from **16** (31 mg, 0.1 mmol) and 1,3,5-trimethoxybenzene (19 mg, 0.12 mmol). Purified over silica gel (PE:EA = 95:5 to 80:20) to afford product **17** as an yellowish wax (23 mg, 54% yield).

M.p. = 70-78°C.

^{1}H NMR (400 MHz, $CDCl_3$): δ 7.57 – 7.55 (m, 1H), 7.20 – 7.12 (m, 2H), 6.80 – 6.78 (m, 1H), 6.13 (s, 2H), 5.12 – 5.08 (t, J = 8.9 Hz, 1H), 4.31 – 4.13 (m, 4H), 3.90 – 3.35 (overlapped signals, 9H: 3.82, br s, 3H; 3.80, s, 3H; 3.35, br s, 3H), 3.11 (dd, J = 13.1, 9.0 Hz, 1H), 2.84 (dd, J = 13.1, 9.0 Hz, 1H), 1.32 – 1.23 (m, 6H).

^{13}C{^{1}H} NMR (100 MHz, $CDCl_3$): δ 171.1 (2 peaks), 160.0, 148.9, 138.3, 128.1, 126.2, 125.6, 123.1, 112.1, 91.1 (broad), 65.3, 61.6, 61.2, 55.6 (broad), 55.3, 39.4, 38.1, 14.1, 14.0.

HRMS (ESI+): calculated for $C_{24}H_{29}O_7^+$ [MH^+]: 429.1908; found: 429.1904.

Crystal structure determination of 6

Experimental

Single crystals of $C_{25}H_{30}O_7$ (**6**) were obtained from an EtOAc solution. A suitable crystal was selected and analyzed on a STOE STADIVARI diffractometer. The crystal was kept at 150 K during data collection. Using Olex2 [3], the structure was solved with the SHELXT [4] structure solution program using Intrinsic Phasing and refined with the SHELXL [5] refinement package using Least Squares minimisation.

Crystal Data

$C_{25}H_{30}O_7$ (M =442.49 g/mol): triclinic, space group P-1 (no. 2), a = 7.9422(11) Å, b = 9.7764(15) Å, c = 15.865(2) Å, α = 103.980(12)°, β = 96.137(12)°, γ = 104.119(11)°, V = 1141.1(3) Å^3, Z = 2, T = 150 K, μ(Mo Kα) = 0.094 mm^{-1}, $Dcalc$ = 1.288 g/cm^3, 10592 reflections measured (5.374° ≤ 2Θ ≤ 58.246°), 5295 unique (R_{int} = 0.0389, R_{sigma} = 0.0546) which were used in all calculations. The final R_1 was 0.0634 (I > 2σ(I)) and wR_2 was 0.2190 (all data).

Table 1 Crystal data and structure refinement for **6**.

Identification code	JAPB67-1
Empirical formula	$C_{25}H_{30}O_7$
Formula weight	442.49
Temperature/K	150
Crystal system	triclinic
Space group	P-1
a/Å	7.9422(11)
b/Å	9.7764(15)
c/Å	15.865(2)
α/°	103.980(12)
β/°	96.137(12)
γ/°	104.119(11)
Volume/Å^3	1141.1(3)
Z	2
ρ_{calc}g/cm^3	1.288
μ/mm^{-1}	0.094
F(000)	472.0
Crystal size/mm^3	? × ? × ?
Radiation	Mo Kα (λ = 0.71073)
2Θ range for data collection/°	5.374 to 58.246
Index ranges	-10 ≤ h ≤ 10, -12 ≤ k ≤ 12, -15 ≤ l ≤ 21

Reflections collected	10592
Independent reflections	5295 [R_{int} = 0.0389, R_{sigma} = 0.0546]
Data/restraints/parameters	5295/8/304
Goodness-of-fit on F^2	1.173
Final R indexes [I>=2σ (I)]	R_1 = 0.0634, wR_2 = 0.1949
Final R indexes [all data]	R_1 = 0.0899, wR_2 = 0.2190
Largest diff. peak/hole / e Å^{-3}	0.55/-0.40

Table 2 Fractional Atomic Coordinates (×10^4) and Equivalent Isotropic Displacement Parameters (Å^2×10^3) for **6**. U_{eq} is defined as 1/3 of the trace of the orthogonalised U_{IJ} tensor.

**Atom**	***x***	***y***	***z***	**U(eq)**
O001	2089.4(18)	9219.8(15)	1758.5(10)	33.0(4)
O002	4177.7(17)	5279.9(15)	2074.5(9)	31.3(4)
O003	6765.8(19)	8307.3(17)	292.7(10)	36.2(4)
O004	1871(3)	7588(2)	5537.0(11)	56.0(5)
O005	3748(2)	6383(3)	5012.4(11)	60.7(6)
O006	6(3)	8314(3)	3934.9(15)	80.0(8)
C007	3280(2)	8442(2)	1555.6(13)	25.9(4)
C008	5625(2)	7907(2)	833.4(13)	28.0(4)
C009	3157(2)	7255(2)	1907.8(12)	25.4(4)
C00A	592(2)	5317(2)	2106.3(13)	26.0(4)
O00B	-1628(3)	6207(3)	4044.3(17)	83.4(8)
C00C	2690(3)	7212(2)	3439.4(13)	28.5(4)
C00D	5554(2)	6722(2)	1165.7(13)	28.5(4)
C00E	4327(2)	6415(2)	1704.4(13)	26.4(4)
C00F	4508(3)	8786(2)	1023.5(13)	28.2(4)
C00G	1804(2)	6873(2)	2482.7(12)	25.3(4)
C00H	5390(3)	4426(2)	1943.6(15)	33.6(5)
C00I	185(3)	4375(2)	2627.5(14)	29.9(5)
C00J	1452(3)	6484(2)	3985.4(13)	30.2(5)
C00K	2373(3)	6911(2)	4938.8(14)	36.9(5)
C00L	-185(3)	4857(2)	1216.2(14)	32.8(5)
C00M	-973(3)	2976(2)	2241.6(17)	37.8(5)
C00N	-1324(3)	3474(3)	845.9(16)	40.1(6)
C00O	968(3)	4824(2)	3585.5(14)	36.1(5)
C00P	8011(3)	7492(3)	114.4(16)	39.9(5)
C00Q	-1708(3)	2523(2)	1357.4(17)	44.6(6)
C00R	-236(3)	6971(3)	3994.2(15)	43.2(6)
C00S	2067(3)	10369(3)	1366.5(17)	44.1(6)
C00T	4755(4)	6700(4)	5890.6(16)	60.0(8)
C00U	-1720(4)	9381(3)	3132.2(19)	62.7(8)
C00V	6378(5)	7907(4)	6023(2)	92.2(12)
C00W	-1073(10)	9324(8)	4047(5)	51.2(15)
C00X	-1856(7)	8541(7)	3742(4)	48.7(13)

Table 3 Anisotropic Displacement Parameters (Å^2×10^3) for **6**. The Anisotropic displacement factor exponent takes the form: $-2\pi^2[h^2a^{*2}U_{11}+2hka^*b^*U_{12}+…]$.

Atom	U_{11}	U_{22}	U_{33}	U_{23}	U_{13}	U_{12}
O001	35.7(8)	33.4(8)	40.0(8)	16.2(6)	16.4(7)	18.0(6)
O002	27.5(8)	36.1(8)	40.2(8)	18.1(6)	16.1(6)	15.1(6)
O003	29.6(8)	45.7(8)	45.2(9)	24.4(7)	19.9(7)	14.8(6)
O004	73.8(13)	63.6(12)	35.1(9)	5.4(8)	9.8(9)	35.4(10)
O005	51.7(11)	104.3(16)	33.6(9)	14.4(9)	4.4(8)	41.3(11)
O006	98.9(17)	113.4(19)	98.7(17)	78.3(15)	71.7(14)	87.3(15)
C007	23.2(10)	27.1(9)	27.1(10)	5.8(8)	3.7(8)	8.4(7)
C008	21.8(10)	31.1(10)	29.2(10)	8.5(8)	6.0(8)	3.1(8)
C009	20.9(10)	30.5(10)	23.6(9)	5.7(8)	5.1(8)	6.4(7)
C00A	17.0(9)	31.2(10)	30.9(10)	6.1(8)	8.9(8)	9.1(7)
O00B	36.1(11)	92.2(16)	107.7(18)	-8.0(14)	29.9(12)	19.8(10)
C00C	26.0(10)	31.1(10)	27.3(10)	6.7(8)	7.1(8)	6.2(8)
C00D	22.1(10)	30.8(10)	33.4(11)	7.7(8)	8.5(8)	8.7(8)
C00E	22.7(10)	27.4(9)	29.2(10)	8.6(8)	4.4(8)	6.5(7)
C00F	25.8(10)	26.7(9)	32.4(10)	10.3(8)	6.7(8)	5.0(8)
C00G	21.9(10)	28.7(9)	29.3(10)	10.6(8)	7.9(8)	10.6(8)
C00H	28.7(11)	34.6(11)	44.0(12)	15.0(9)	12.0(9)	14.4(8)
C00I	21.6(10)	30.0(10)	42.8(12)	12.3(9)	12.2(9)	10.8(8)
C00J	28.5(11)	39.4(11)	27.8(10)	13.7(9)	10.1(8)	12.3(9)
C00K	38.4(13)	45.0(12)	31.6(11)	15.2(10)	13.8(10)	12.0(10)
C00L	22.0(10)	41.7(11)	34.5(11)	7.0(9)	8.9(9)	10.4(8)
C00M	26.3(11)	30.6(11)	60.4(15)	14.3(10)	14.5(10)	10.8(8)
C00N	22.6(11)	47.3(13)	40.0(12)	-3.1(10)	8.4(9)	4.6(9)
C00O	35.1(12)	39.6(11)	38.2(12)	20.5(9)	7.7(9)	8.7(9)
C00P	29.1(11)	54.9(14)	50.7(14)	29.1(11)	19.7(10)	19.4(10)
C00Q	25.1(11)	33.4(11)	61.9(16)	-6.2(11)	12.4(11)	0.9(9)
C00R	41.3(14)	65.3(16)	31.8(12)	12.9(11)	15.8(10)	27.6(12)
C00S	52.9(15)	40.1(12)	56.2(15)	25.0(11)	23.2(12)	26.4(11)
C00T	55.3(17)	96(2)	31.5(13)	16.0(14)	-1.5(12)	31.5(16)
C00U	58.0(18)	70.4(19)	55.6(17)	3.5(14)	-6.9(14)	31.4(15)
C00V	110(3)	87(2)	58(2)	22.2(18)	-23(2)	3(2)
C00W	49(4)	50(3)	63(4)	14(3)	12(3)	31(3)
C00X	39(3)	54(3)	65(3)	21(3)	15(2)	28(2)

Table 4 Bond Lengths for **6**.

Atom	Atom	Length/Å	Atom	Atom	Length/Å
O001	C007	1.369(2)	C00A	C00G	1.523(3)
O001	C00S	1.412(3)	C00A	C00I	1.385(3)
O002	C00E	1.362(2)	C00A	C00L	1.396(3)
O002	C00H	1.421(2)	O00B	C00R	1.196(3)
O003	C008	1.366(2)	C00C	C00G	1.524(3)
O003	C00P	1.425(2)	C00C	C00J	1.537(3)
O004	C00K	1.185(3)	C00D	C00E	1.389(3)
O005	C00K	1.323(3)	C00I	C00M	1.398(3)

Table 4 Bond Lengths for **6**.

Atom	Atom	Length/Å	Atom	Atom	Length/Å
O005	C00T	1.453(3)	C00I	C00O	1.494(3)
O006	C00R	1.308(3)	C00J	C00K	1.519(3)
O006	C00W	1.449(6)	C00J	C00O	1.527(3)
O006	C00X	1.557(5)	C00J	C00R	1.528(3)
C007	C009	1.392(3)	C00L	C00N	1.377(3)
C007	C00F	1.389(3)	C00M	C00Q	1.380(4)
C008	C00D	1.376(3)	C00N	C00Q	1.377(4)
C008	C00F	1.385(3)	C00T	C00V	1.476(4)
C009	C00E	1.397(3)	C00U	C00W	1.506(7)
C009	C00G	1.518(2)	C00U	C00X	1.409(6)

Table 5 Bond Angles for **6**.

Atom	Atom	Atom	Angle/˚	Atom	Atom	Atom	Angle/˚
C007	O001	C00S	117.68(15)	C009	C00G	C00C	110.90(15)
C00E	O002	C00H	118.63(15)	C00A	C00G	C00C	113.92(16)
C008	O003	C00P	117.16(16)	C00A	C00I	C00M	118.9(2)
C00K	O005	C00T	117.51(19)	C00A	C00I	C00O	121.27(18)
C00R	O006	C00W	133.1(4)	C00M	C00I	C00O	119.9(2)
C00R	O006	C00X	106.9(3)	C00K	C00J	C00C	109.63(17)
O001	C007	C009	115.21(16)	C00K	C00J	C00O	110.73(17)
O001	C007	C00F	122.88(17)	C00K	C00J	C00R	106.76(16)
C00F	C007	C009	121.90(18)	C00O	C00J	C00C	108.02(16)
O003	C008	C00D	123.65(18)	C00O	C00J	C00R	109.14(18)
O003	C008	C00F	114.76(18)	C00R	C00J	C00C	112.58(18)
C00D	C008	C00F	121.58(18)	O004	C00K	O005	125.0(2)
C007	C009	C00E	117.14(17)	O004	C00K	C00J	123.6(2)
C007	C009	C00G	121.85(16)	O005	C00K	C00J	111.38(17)
C00E	C009	C00G	121.01(17)	C00N	C00L	C00A	121.1(2)
C00I	C00A	C00G	122.00(17)	C00Q	C00M	C00I	121.3(2)
C00I	C00A	C00L	119.32(18)	C00Q	C00N	C00L	119.8(2)
C00L	C00A	C00G	118.63(18)	C00I	C00O	C00J	112.17(17)
C00G	C00C	C00J	111.94(16)	C00N	C00Q	C00M	119.6(2)
C008	C00D	C00E	118.46(17)	O006	C00R	C00J	112.5(2)
O002	C00E	C009	115.13(16)	O00B	C00R	O006	123.6(2)
O002	C00E	C00D	122.65(17)	O00B	C00R	C00J	123.8(3)
C00D	C00E	C009	122.23(18)	O005	C00T	C00V	110.1(2)
C008	C00F	C007	118.68(18)	O006	C00W	C00U	105.8(4)
C009	C00G	C00A	112.30(15)	C00U	C00X	O006	105.1(3)

Table 6 Torsion Angles for **6**.

A	B	C	D	Angle/˚	A	B	C	D	Angle/˚
O001	C007	C009	C00E	178.89(16)	C00I	C00A	C00G	C009	134.49(18)
O001	C007	C009	C00G	-0.6(3)	C00I	C00A	C00G	C00C	7.4(2)
O001	C007	C00F	C008	-178.08(17)	C00I	C00A	C00L	C00N	-1.1(3)

Table 6 Torsion Angles for **6**.

A	B	C	D	Angle/˚	A	B	C	D	Angle/˚
O003	C008	C00D	C00E	-179.49(17)	C00I	C00M	C00Q	C00N	-1.2(3)
O003	C008	C00F	C007	178.79(17)	C00J	C00C	C00G	C009	-165.32(16)
C007	C009	C00E	O002	178.99(16)	C00J	C00C	C00G	C00A	-37.5(2)
C007	C009	C00E	C00D	-0.8(3)	C00K	O005	C00T	C00V	-101.0(3)
C007	C009	C00G	C00A	121.9(2)	C00K	C00J	C00O	C00I	-175.55(17)
C007	C009	C00G	C00C	-109.3(2)	C00K	C00J	C00R	O006	89.4(2)
C008	C00D	C00E	O002	-178.94(17)	C00K	C00J	C00R	O00B	-91.0(3)
C008	C00D	C00E	C009	0.8(3)	C00L	C00A	C00G	C009	-48.2(2)
C009	C007	C00F	C008	0.7(3)	C00L	C00A	C00G	C00C	-175.36(16)
C00A	C00I	C00M	C00Q	0.1(3)	C00L	C00A	C00I	C00M	1.1(3)
C00A	C00I	C00O	C00J	27.1(3)	C00L	C00A	C00I	C00O	-179.33(18)
C00A	C00L	C00N	C00Q	0.0(3)	C00L	C00N	C00Q	C00M	1.2(3)
C00C	C00J	C00K	O004	117.4(2)	C00M	C00I	C00O	C00J	-153.33(18)
C00C	C00J	C00K	O005	-65.2(2)	C00O	C00I	C00M	C00Q	-179.52(19)
C00C	C00J	C00O	C00I	-55.5(2)	C00O	C00J	C00K	O004	-123.6(2)
C00C	C00J	C00R	O006	-30.9(3)	C00O	C00J	C00K	O005	53.9(2)
C00C	C00J	C00R	O00B	148.6(2)	C00O	C00J	C00R	O006	-150.9(2)
C00D	C008	C00F	C007	-0.7(3)	C00O	C00J	C00R	O00B	28.7(3)
C00E	C009	C00G	C00A	-57.5(2)	C00P	O003	C008	C00D	-3.9(3)
C00E	C009	C00G	C00C	71.2(2)	C00P	O003	C008	C00F	176.62(18)
C00F	C007	C009	C00E	0.0(3)	C00R	O006	C00W	C00U	-106.6(5)
C00F	C007	C009	C00G	-179.47(17)	C00R	O006	C00X	C00U	-141.1(4)
C00F	C008	C00D	C00E	-0.1(3)	C00R	C00J	C00K	O004	-4.9(3)
C00G	C009	C00E	O002	-1.5(3)	C00R	C00J	C00K	O005	172.6(2)
C00G	C009	C00E	C00D	178.68(17)	C00R	C00J	C00O	C00I	67.2(2)
C00G	C00A	C00I	C00M	178.33(17)	C00S	O001	C007	C009	-175.45(18)
C00G	C00A	C00I	C00O	-2.1(3)	C00S	O001	C007	C00F	3.4(3)
C00G	C00A	C00L	C00N	-178.48(17)	C00T	O005	C00K	O004	-2.3(4)
C00G	C00C	C00J	C00K	-177.56(16)	C00T	O005	C00K	C00J	-179.7(2)
C00G	C00C	C00J	C00O	61.7(2)	C00W	O006	C00R	O00B	11.3(6)
C00G	C00C	C00J	C00R	-58.9(2)	C00W	O006	C00R	C00J	-169.1(4)
C00H	O002	C00E	C009	-176.25(16)	C00X	O006	C00R	O00B	-11.9(4)
C00H	O002	C00E	C00D	3.6(3)	C00X	O006	C00R	C00J	167.7(3)

Table 7 Hydrogen Atom Coordinates (Å×10^4) and Isotropic Displacement Parameters (Å^2×10^3) for **6**.

Atom	*x*	*y*	*z*	U(eq)
H00A	3065.54	8287.95	3706.64	34
H00B	3756.88	6859.28	3455.57	34
H00D	6326.17	6126.91	1029.81	34
H00F	4580.79	9606.85	794.47	34
H00G	1038.18	7546.61	2476.59	30
H00C	5259.84	3945.69	1312.56	50
H00E	5150.89	3677.95	2263.75	50
H00H	6596.16	5063.7	2164.36	50

Table 7 Hydrogen Atom Coordinates (Å×10⁴) and Isotropic Displacement Parameters (Å²×10³) for **6**.

**Atom**	***x***	***y***	***z***	**U(eq)**
H00L	75.84	5508.47	860	39
H00M	-1259.7	2323.15	2595.14	45
H00N	-1842.11	3177.14	239.07	48
H00I	2039.93	4487.82	3660.22	43
H00J	113.43	4338.4	3906.85	43
H00K	8738.36	7870.99	-285.29	60
H00O	7384.72	6454.66	-161.94	60
H00P	8768.06	7588.78	667.96	60
H00Q	-2475.16	1561.21	1103.17	54
H00R	1865.57	9981.29	722.92	66
H00S	3201.21	11126.61	1565.82	66
H00T	1118.09	10797.43	1538.83	66
H00U	5068.19	5810.8	5965.8	72
H00V	4030.98	6979.8	6338.02	72
H00W	-2348.51	8387.71	2764.28	94
H00X	-712.24	9787.47	2873.66	94
H00Y	-2520.03	10005.71	3163.16	94
H	-1418.28	8834.42	2592.93	94
HA	-797.4	10309.8	3389.96	94
HB	-2849.84	9583.45	2989.36	94
H00Z	7063.77	7647.34	5561.26	138
HC	7083.44	8069.9	6602.42	138
HD	6063.75	8805.19	5992.63	138
H00	-368.99	10309.38	4417.59	61
HE	-2079.21	8967.11	4331.35	61
H1	-2760.25	7585.58	3487.69	58
HF	-2175.86	9069.94	4289.39	58

Table 8 Atomic Occupancy for **6**.

**Atom**	***Occupancy***	**Atom**	***Occupancy***	**Atom**	***Occupancy***
H00W	0.448(7)	H00X	0.448(7)	H00Y	0.448(7)
H	0.552(7)	HA	0.552(7)	HB	0.552(7)
C00W	0.448(7)	H00	0.448(7)	HE	0.448(7)
C00X	0.552(7)	H1	0.552(7)	HF	0.552(7)

DFT calculation

Calculations were carried out with the Gaussian16 package [6], and all structures were fully optimized without any symmetry constraints at the DFT level by means of the M06 functional, which is known to perform well for main group compounds and noncovalent bonds and to include partly dispersion forces [7]. The 6-31G(d,p) basis set was applied for all atoms. Each stationary point was characterized with frequency analysis and shows the correct number of negative eigenvalues (zero for a local minimum and one for a transition state). The transition state **TS** was verified by stepping along the reaction coordinate (intrinsic reaction coordinate calculation) and confirming that it transformed into the corresponding reactant/product. Final energy calculations at the M06 level associated with the 6-311++G(2d,2p) basis set, including solvation effect, was achieved on the M06/6-31G(d,p) gas phase geometries. The solvent effect is accounted for by means of single point calculations with the integral equation formalism version of the polarizable continuum model (IEFPCM) for dichloromethane. To get accurate geometries and energies, the SCF convergence criterion was systematically tightened to 10^{-8} au, and the force minimizations were carried out until the rms force became smaller that (at least) 1 x 10^{-5} au (“tight” optimization keyword in Gaussian). The “UltraFine” grid (99 radial shells and 590 angular points per shell) was used throughout the calculations. Cartesian coordinates of the optimized geometries and associated energies and free energy corrections obtained.

Cartesian coordinates of the optimized geometries and associated energies and free energy corrections obtained at the IEFPCM(CH_2Cl_2)-M06/6-311++G(2d,2p)//M06/6-31G(d,p) level of theory

Structure 7

C 2.052719000 0.345516000 0.039207000
C 2.825784000 -0.433868000 0.779800000
H 2.523960000 1.106862000 -0.584323000
O 4.241142000 -0.213289000 0.788764000
C 4.557928000 -0.962329000 -1.487054000
H 4.360692000 -0.118631000 -2.164465000
H 5.355184000 -1.551716000 -1.948706000
C 5.016048000 -0.430511000 -0.193371000
H 3.631717000 -1.536626000 -1.399610000
C 6.434727000 -0.129965000 0.041780000
H 6.986173000 -1.080973000 0.070840000
H 6.847560000 0.428981000 -0.806180000
H 6.586749000 0.407441000 0.978547000

H 2.534525000 -1.161015000 1.528619000
C 0.548678000 0.281583000 -0.041186000
C 0.130556000 -0.735344000 -1.163839000
H 0.341935000 -0.266653000 -2.133047000
H 0.808435000 -1.593454000 -1.057186000
C -0.074189000 -0.233162000 1.260697000
O 0.411826000 -1.119147000 1.926567000
O -1.239273000 0.345420000 1.476901000
C 0.086440000 1.725875000 -0.254588000
O 0.584640000 2.634069000 0.367007000
O -0.868658000 1.832291000 -1.161641000
C -1.560406000 3.101760000 -1.244030000
H -1.952998000 3.131675000 -2.264276000
H -0.834088000 3.909355000 -1.111428000
C -2.653886000 3.132169000 -0.207990000
H -2.227242000 3.116429000 0.800815000
H -3.253753000 4.041094000 -0.312557000
H -3.311625000 2.262809000 -0.324980000
C -2.079033000 -0.217049000 2.518897000
H -1.949926000 -1.304916000 2.505995000
H -1.715021000 0.160195000 3.480880000
C -3.491677000 0.195929000 2.215464000
H -3.794432000 -0.187070000 1.233518000
H -4.169708000 -0.214211000 2.969583000
H -3.598550000 1.285589000 2.217068000
C -1.289488000 -1.246601000 -1.111748000
C -2.320151000 -0.648050000 -1.838924000
C -1.585936000 -2.362771000 -0.324431000
C -3.618927000 -1.137269000 -1.759695000
H -2.101605000 0.206801000 -2.473869000
C -2.885832000 -2.849348000 -0.237540000
H -0.788918000 -2.855000000 0.232232000
C -3.908034000 -2.233287000 -0.952071000
H -4.408375000 -0.664702000 -2.338839000
H -3.096495000 -3.720378000 0.378021000
H -4.923530000 -2.616322000 -0.893696000

There are no imaginary frequencies.

SCF energy = -1114.244126 hartree (M06/6-31G(d,p))

SCF energy = -1114.587778 hartree (IEFPCM(CH_2Cl_2)-M06/6-311++G(2d,2p)//M06/6-31G(d,p))

Zero-point correction = +0.408147 hartree

Energy correction = +0.434066 hartree

Enthalpy correction = +0.435010 hartree

Gibbs Free Energy correction = +0.350117 hartree

Structure 7’

C 1.92947500 0.18375100 -1.28307500
C 2.26759400 -1.20614900 -0.76763000

H 2.56789300 0.92001800 -0.78235000
O 3.49858300 -1.46458600 -0.32967100
C 3.86490200 -0.02049500 1.53011800
H 2.97265400 -0.38122400 2.03049700
H 4.47154700 0.71716000 2.04392100
C 4.25021900 -0.47453500 0.34499700
H 2.08540200 0.23991000 -2.36457900
C 5.47063700 -0.12099600 -0.41896400
H 6.08599600 0.58967800 0.13683300
H 6.06575000 -1.01840800 -0.62240100
H 5.21213100 0.32022000 -1.39055300
H 1.95306500 -2.03201700 -1.41420100
C 0.46608700 0.42143500 -0.89131900
C -0.53636200 -0.13262500 -1.95491100
H -0.75323900 0.69166400 -2.64267000
H -0.00576600 -0.89936100 -2.53518000
C 0.38152100 -0.43333900 0.33708600
O 1.25205300 -1.35328700 0.43165000
O -0.52879300 -0.24541200 1.19949200
C 0.23316400 1.85826900 -0.40498000
O 0.99805700 2.36549100 0.37953500
O -0.88112700 2.38326700 -0.87354600
C -1.33248300 3.61268100 -0.23828900
H -2.02198100 4.04919300 -0.96437300
H -0.47160200 4.27528900 -0.11108000
C -2.00222200 3.27753500 1.07027300
H -1.27807100 2.87411300 1.78586200
H -2.44573700 4.17656100 1.50782000
H -2.79743900 2.53796500 0.92009300
C -0.72667600 -1.20791500 2.30497200
H -0.77133800 -2.20085400 1.84404200
H 0.15851600 -1.14014300 2.94578100
C -2.00425400 -0.80909100 2.97738400
H -2.83637600 -0.85041000 2.26601600
H -2.21489500 -1.50218000 3.79661000
H -1.93539600 0.20068000 3.39214900
C -1.79862300 -0.73312200 -1.39356500
C -2.93372000 0.03838400 -1.14356300
C -1.81652700 -2.08976500 -1.05653100
C -4.05712600 -0.53212100 -0.55438200
H -2.93498300 1.09100500 -1.41756900
C -2.93695800 -2.66186500 -0.46200200
H -0.94800200 -2.71311300 -1.27885500
C -4.05938200 -1.87991900 -0.20486400
H -4.94037500 0.07643800 -0.37709500
H -2.94166100 -3.72179300 -0.22028000
H -4.94192900 -2.32493000 0.24707300

There are no imaginary frequencies.

SCF energy = -1114.258938 hartree (M06/6-31G(d,p))

SCF energy = -1114.590680 hartree (IEFPCM(CH_2Cl_2)-M06/6-311++G(2d,2p)//M06/6-31G(d,p))

Zero-point correction = +0.412434 hartree

Energy correction = +0.436497 hartree

Enthalpy correction = +0.437441 hartree

Gibbs Free Energy correction = +0.358485 hartree

Transition state (TS)

C 2.088309000 0.208200000 0.336641000
C 2.742642000 -0.981244000 0.601335000
H 2.366265000 1.097981000 0.911721000
O 4.064443000 -1.117573000 0.531282000
C 4.463148000 0.976179000 -0.513679000
H 4.156726000 1.680197000 0.256087000
H 5.019119000 1.446208000 -1.323452000
C 4.864977000 -0.307417000 -0.185493000
H 3.049111000 0.646098000 -0.602616000
C 6.055898000 -1.018794000 -0.692955000
H 6.588184000 -0.409442000 -1.425270000
H 6.741524000 -1.207690000 0.143823000
H 5.794181000 -1.991104000 -1.121783000
H 2.249708000 -1.940197000 0.746541000
C 0.602752000 0.218587000 0.049222000
C 0.216282000 -0.685996000 -1.162555000
H 0.454579000 -0.133859000 -2.080012000
H 0.882312000 -1.559931000 -1.138501000
C -0.132202000 -0.331853000 1.290612000
O 0.306222000 -1.244716000 1.950888000
O -1.292647000 0.266287000 1.453455000
C 0.231414000 1.700559000 -0.095397000
O 0.742569000 2.543014000 0.608934000
O -0.666889000 1.912321000 -1.033713000
C -1.287272000 3.223375000 -1.079534000
H -1.603165000 3.336521000 -2.119971000
H -0.531297000 3.978680000 -0.845359000
C -2.447697000 3.245469000 -0.119863000
H -2.096222000 3.131694000 0.911419000
H -2.986377000 4.194538000 -0.195957000
H -3.143413000 2.429334000 -0.346752000
C -2.214285000 -0.321619000 2.413347000
H -2.118247000 -1.410833000 2.345474000
H -1.896489000 -0.009458000 3.414089000
C -3.589974000 0.158222000 2.047597000
H -3.839900000 -0.153652000 1.026357000
H -4.327878000 -0.272614000 2.730497000
H -3.662084000 1.248578000 2.111696000
C -1.207267000 -1.187494000 -1.193152000
C -2.213177000 -0.513903000 -1.888226000
C -1.535074000 -2.360984000 -0.508875000
C -3.519026000 -0.991146000 -1.880680000
H -1.969454000 0.389762000 -2.441549000
C -2.842127000 -2.835671000 -0.492183000
H -0.757323000 -2.910288000 0.022076000
C -3.839405000 -2.147865000 -1.175901000
H -4.289326000 -0.461164000 -2.435355000
H -3.077498000 -3.752467000 0.042427000
H -4.860112000 -2.521206000 -1.173951000

There is one imaginary frequency = -1216.22 cm^{-1}.

SCF energy = -1114.189062 hartree (M06/6-31G(d,p))

SCF energy = -1114.526228 hartree (IEFPCM(CH_2Cl_2)-M06/6-311++G(2d,2p)//M06/6-31G(d,p))

Zero-point correction = +0.405340 hartree

Energy correction = +0.429687 hartree

Enthalpy correction = +0.430632 hartree

Gibbs Free Energy correction = +0.350972 hartree

References

(1) Flachsbarth, B.; Fritzsche, M.; Weldon, P. J.; Schulz, S. *Chem. Biodivers.* **2009**, *6*, 1–37. doi:10.1002/cbdv.200800265

(2) Cacciuttolo, B.; Poulain-Martini, S.; Fontaine-Vive, F.; Abdo, M. A. H.; El-Kashef, H.; Duñach, E. *Eur. J. Org. Chem.* **2014**, *2014*, 7458–7468. doi:10.1002/ejoc.201402972

(3) Dolomanov, O. V.; Bourhis, L. J.; Gildea, R. J.; Howard, J. a. K.; Puschmann, H. *J. Appl. Crystallogr.* **2009**, *42*, 339–341. doi:10.1107/S0021889808042726

(4) Sheldrick, G. M. *Acta Crystallogr. Sect. Found. Adv.* **2015**, *71*, 3–8. doi:10.1107/S2053273314026370

(5) Sheldrick, G. M. *Acta Crystallogr. Sect. C Struct. Chem.* **2015**, *71*, 3–8. doi:10.1107/S2053229614024218

(6) Frisch, M. J.; Trucks, G. W.; Schlegel, H. B.; Scuseria, G. E.; Robb, M. A.; Cheeseman, J. R.; Scalmani, G.; Barone, V.; Petersson, G. A.; Nakatsuji, H.; Li, X.; Caricato, M.; Marenich, A. V.; Bloino, J.; Janesko, B. G.; Gomperts, R.; Mennucci, B.; Hratchian, H. P.; Ortiz, J. V.; Izmaylov, A. F.; Sonnenberg, J. L.; Williams-Young, D.; Ding, F.; Lipparini, F.; Egidi, F.; Goings, J.; Peng, B.; Petrone, A.; Henderson, T.; Ranasinghe, D.; Zakrzewski, V. G.; Gao, J.; Rega, N.; Zheng, G.; Liang, W.; Hada, M.; Ehara, M.; Toyota, K.; Fukuda, R.; Hasegawa, J.; Ishida, M.; Nakajima, T.; Honda, Y.; Kitao, O.; Nakai, H.; Vreven, T.; Throssell, K.; Montgomery, J. A., Jr.; Peralta, J. E.; Ogliaro, F.; Bearpark, M. J.; Heyd, J. J.; Brothers, E. N.; Kudin, K. N.; Staroverov, V. N.; Keith, T. A.; Kobayashi, R.; Normand, J.; Raghavachari, K.; Rendell, A. P.; Burant, J. C.; Iyengar, S. S.; Tomasi, J.; Cossi, M.; Millam, J. M.; Klene, M.; Adamo, C.; Cammi, R.; Ochterski, J. W.; Martin, R. L.; Morokuma, K.; Farkas, O.; Foresman, J. B.; Fox, D. J. *Gaussian, Inc., Wallingford CT, Gaussian 16, Revision C.01*; 2016

(7) Zhao, Y.; Truhlar, D. G. *Theor. Chem. Acc.* **2008**, *120*, 215–241. doi:10.1007/s00214-007-0310-x

Copies of spectra

Diethyl (3-methylbut-2-enyl)malonate (S1)

^{1}H NMR (400 MHz, $CDCl_3$)

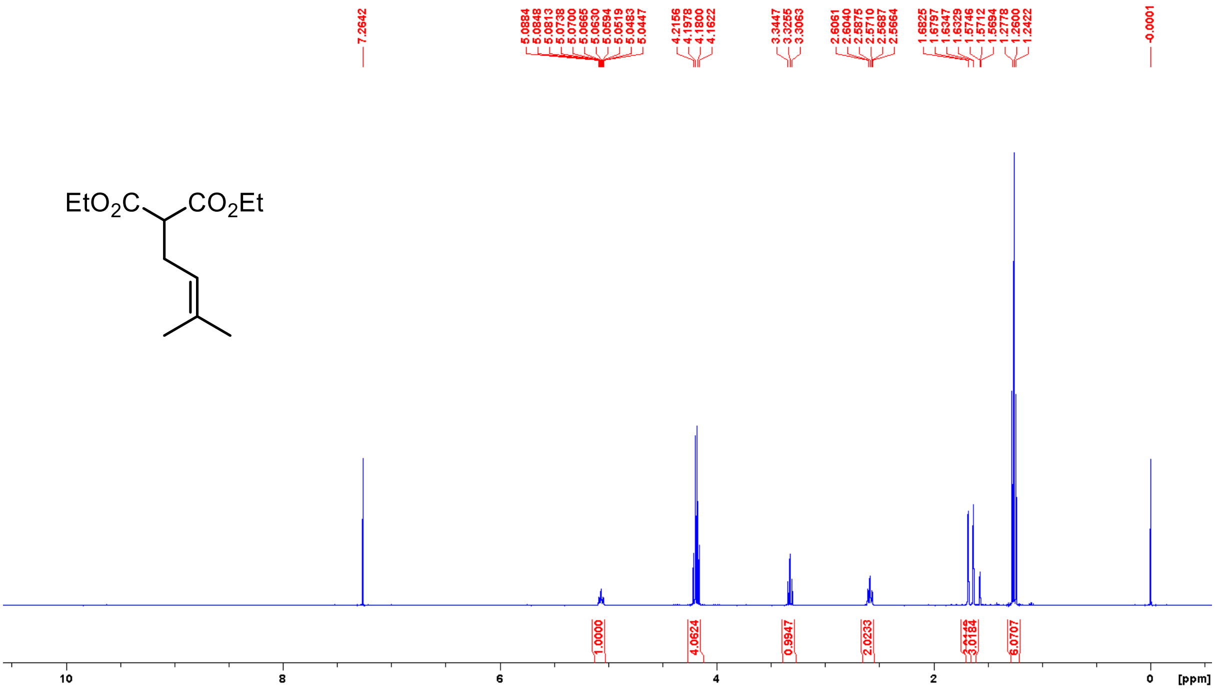

^{13}C{^{1}H} NMR (100 MHz, $CDCl_3$)

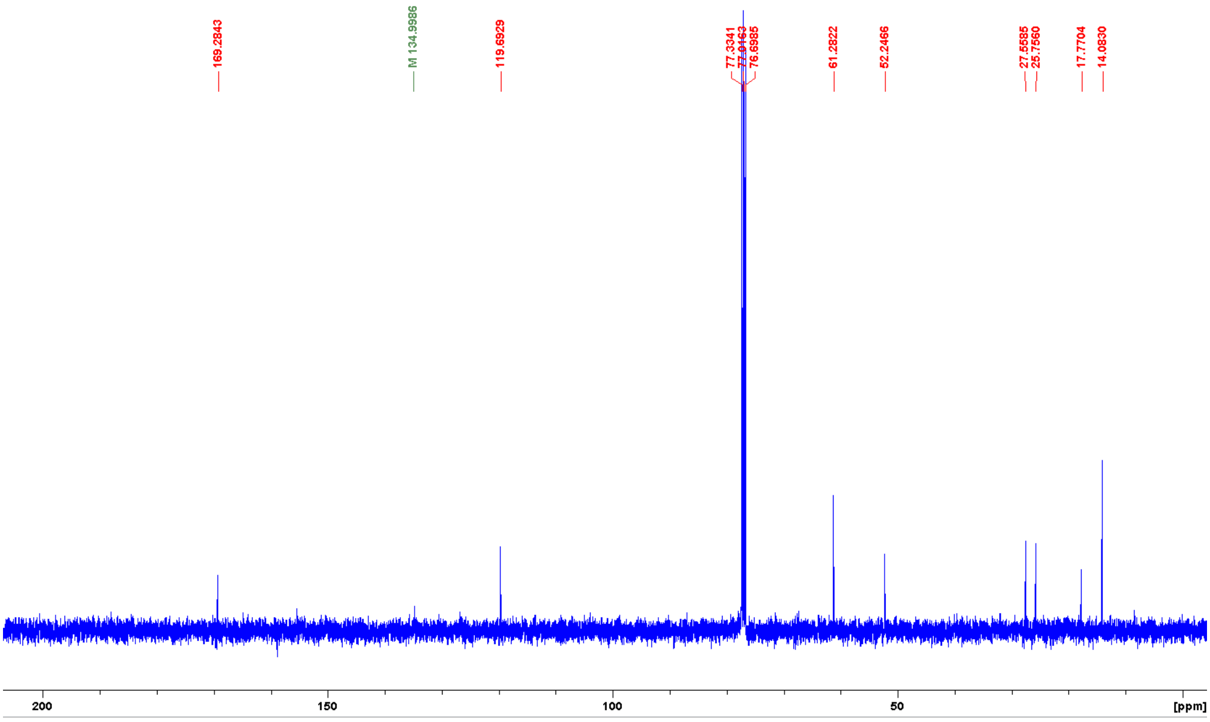

Diethyl benzyl(3-methylbut-2-enyl)malonate (1)

^{1}H NMR (400 MHz, $CDCl_3$)

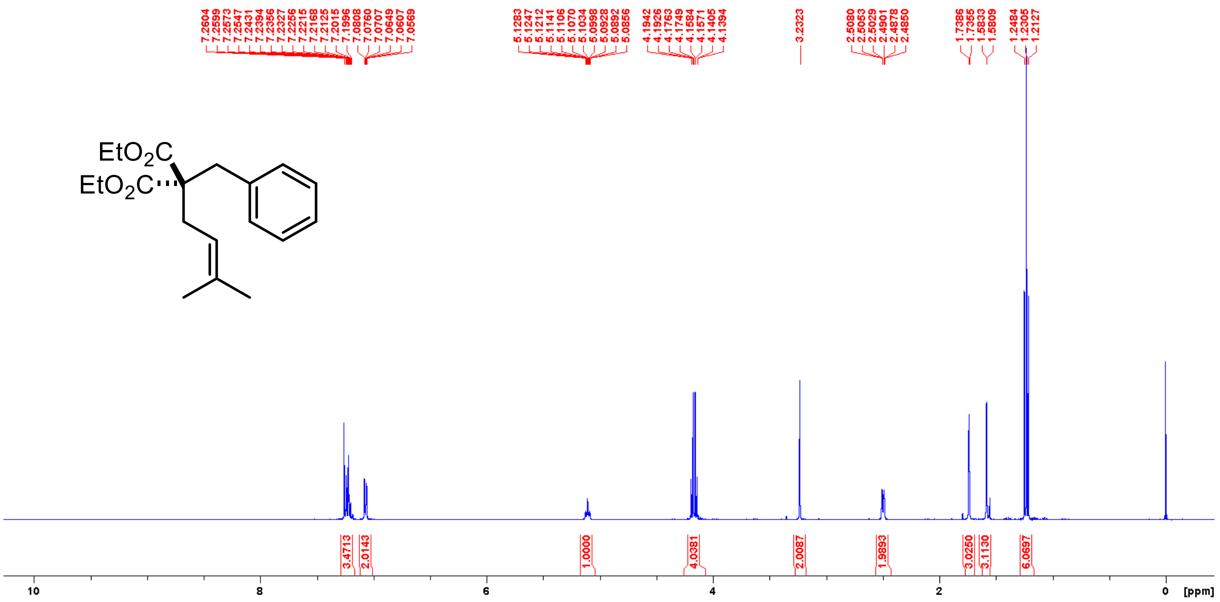

$^{13}C\{^{1}H\}$ NMR (100 MHz, $CDCl_3$)

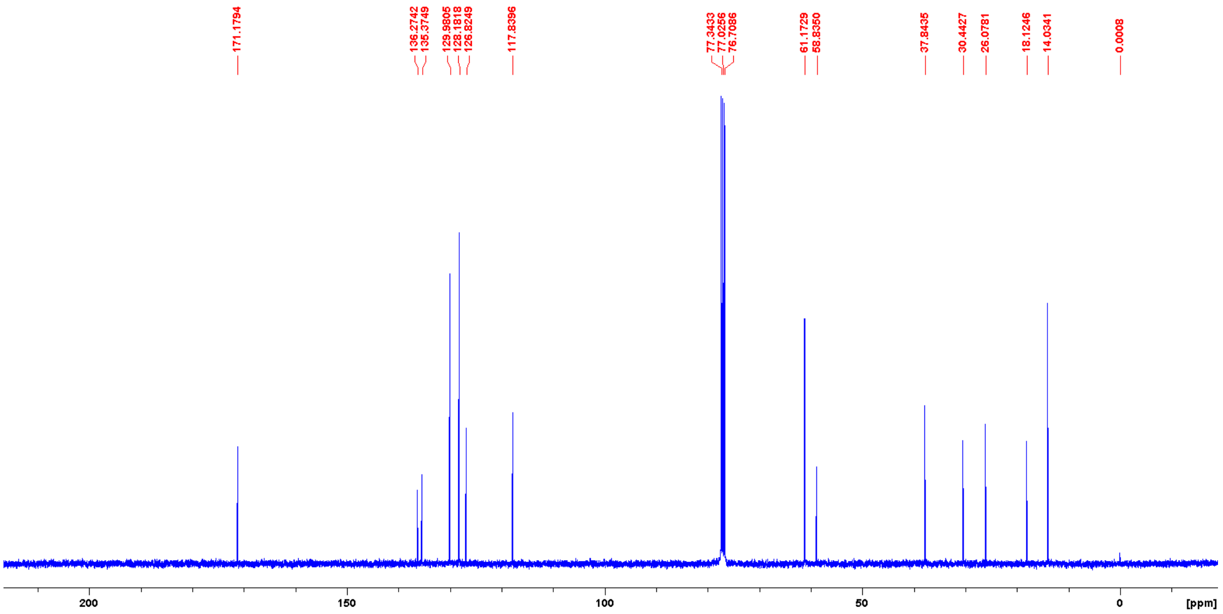

Diethyl (1*H*)naphthalene-2,2-dicarboxylate (4)

^{1}H NMR (400 MHz, $CDCl_3$)

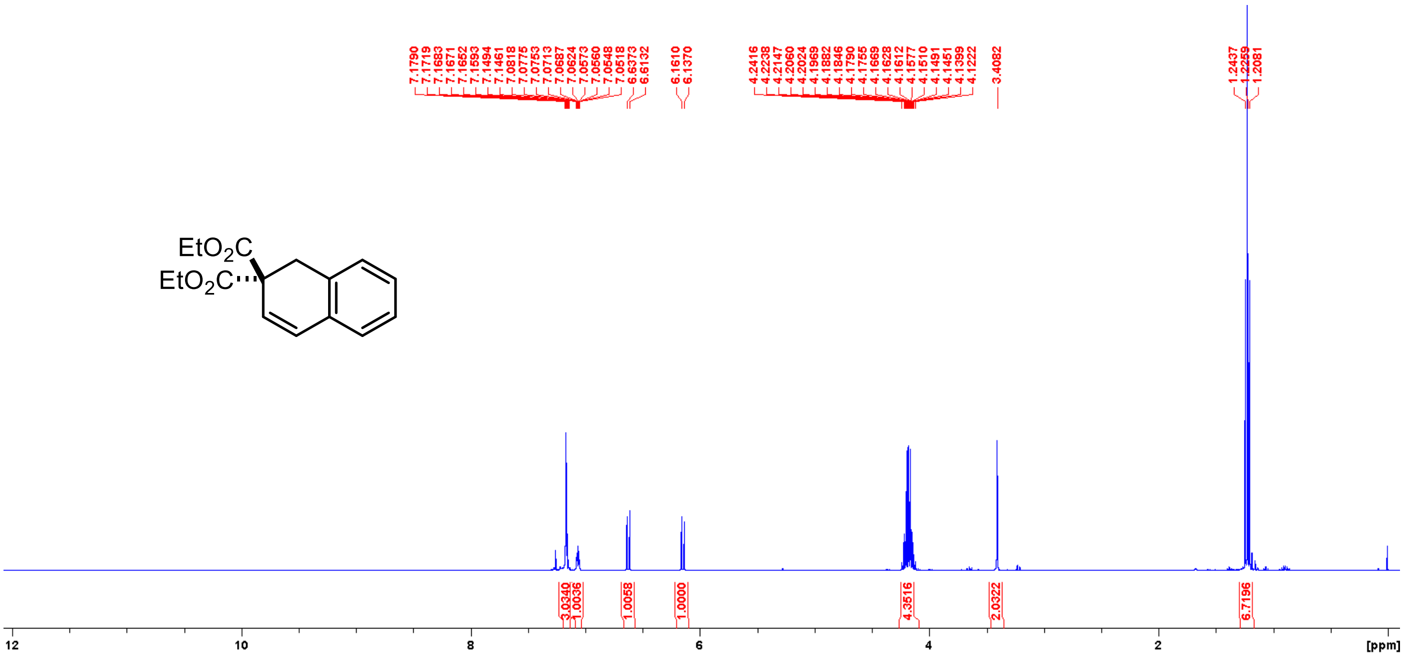

$^{13}C\{^{1}H\}$ NMR (100 MHz, $CDCl_3$)

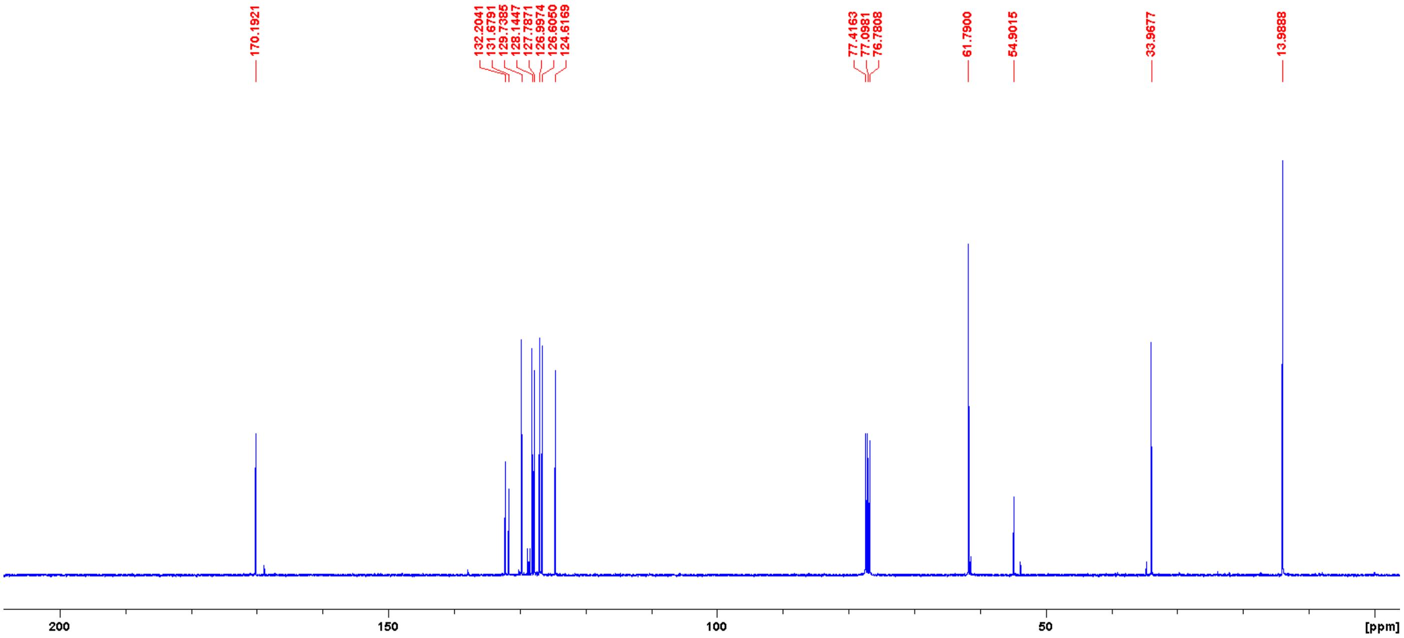

3,3-Bis(ethoxycarbonyl)-1-(2,4,6-trimethoxyphenyl)-1,2,3,4-tetrahydronaphthalene (6)

^{1}H NMR (400 MHz, $CDCl_3$)

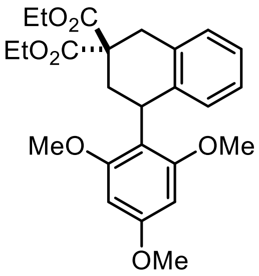

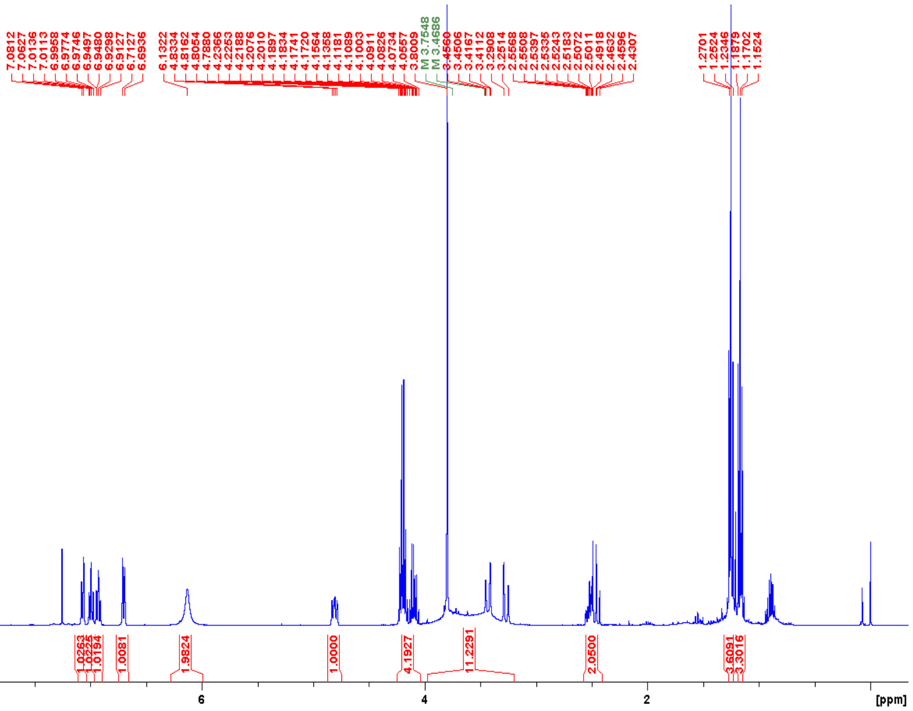

$^{13}C\{^1H\}$ NMR (100 MHz, $CDCl_3$)

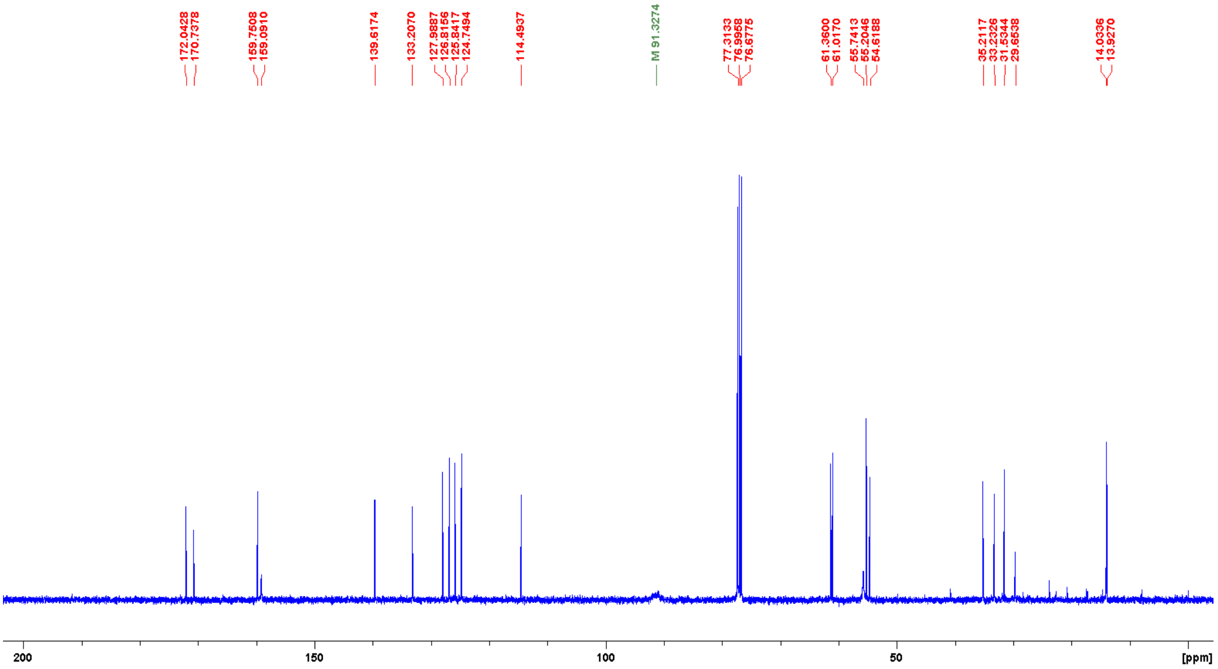

Diethyl (2-methylbenzyl)(3-methylbut-2-enyl)malonate (11a)

^{1}H NMR (400 MHz, $CDCl_3$)

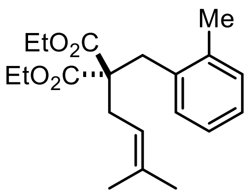

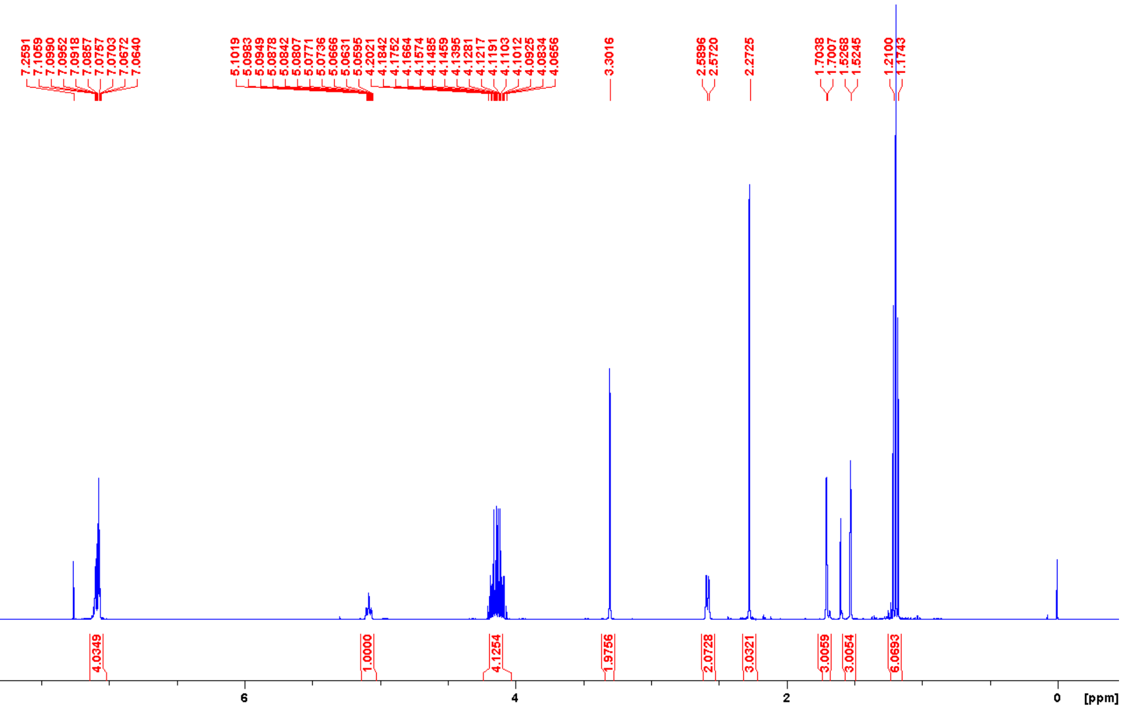

$^{13}C\{^{1}H\}$ NMR (100 MHz, $CDCl_3$)

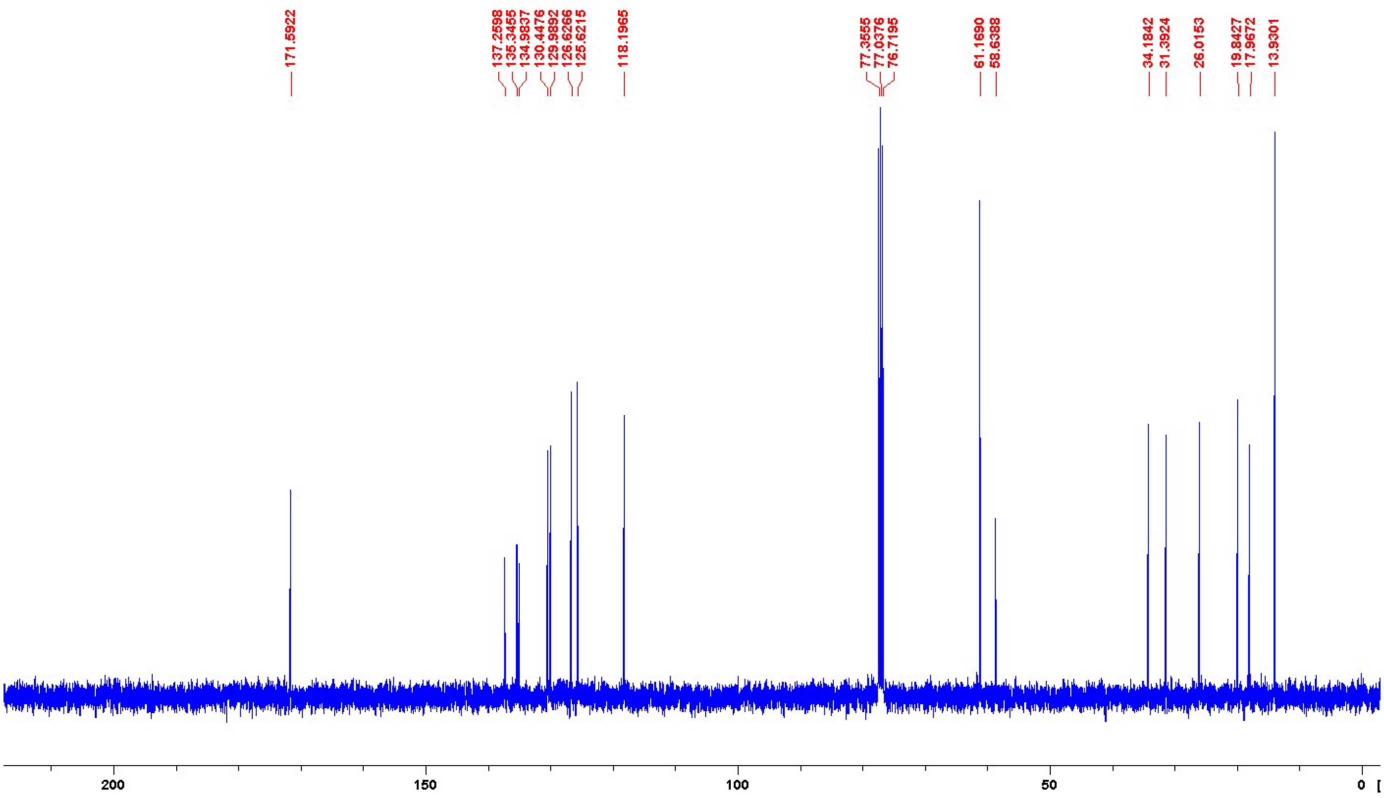

Diethyl (2-fluorobenzyl)(3-methylbut-2-enyl)malonate (11b)

^{1}H NMR (400 MHz, $CDCl_3$)

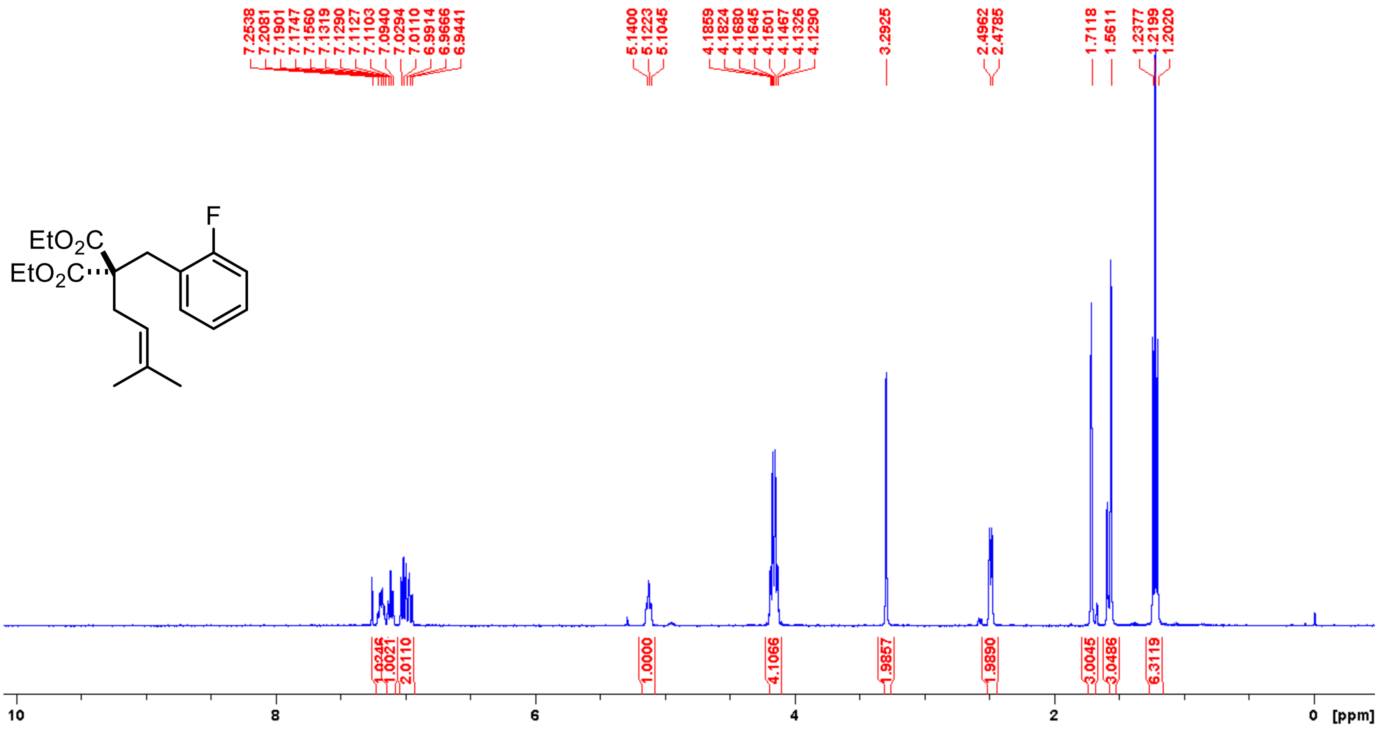

$^{13}C\{^{1}H\}$ NMR (100 MHz, $CDCl_3$)

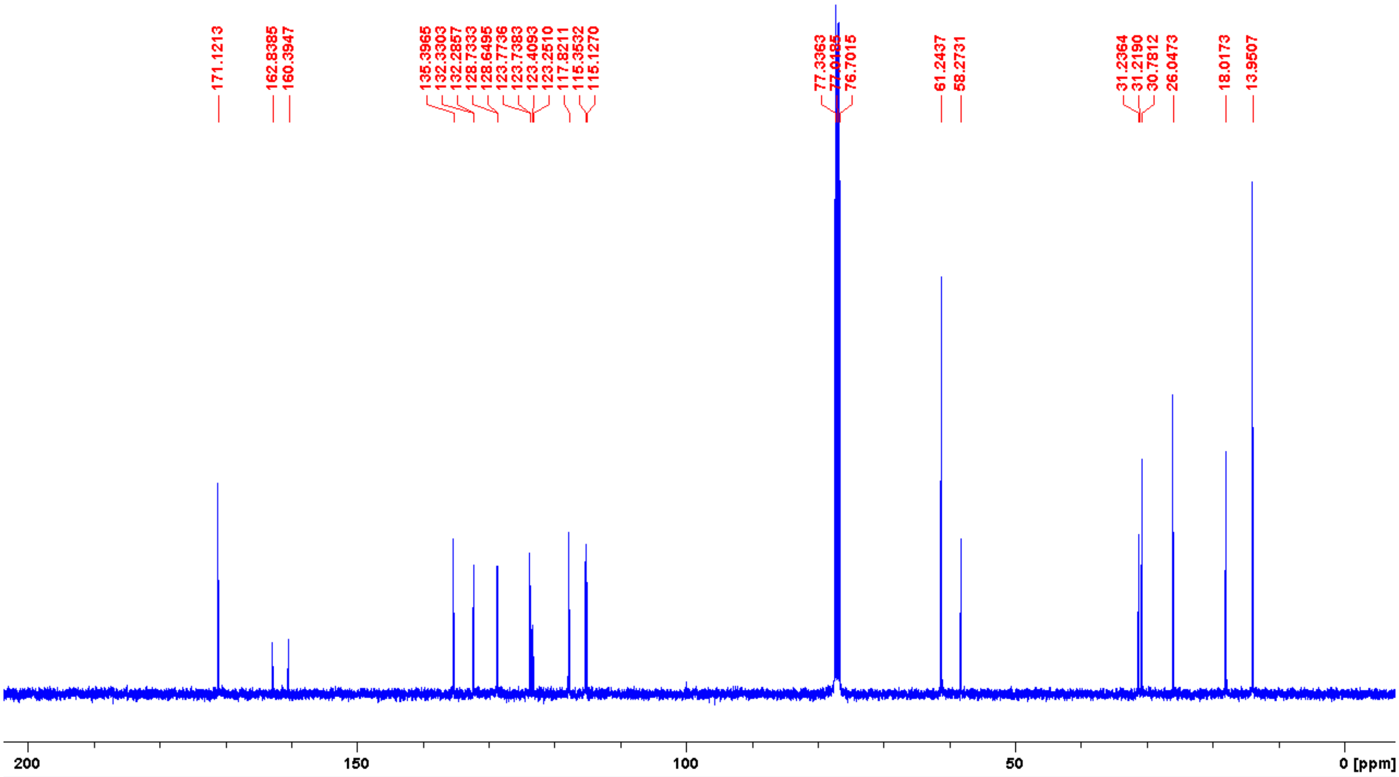

Diethyl (2-chlorobenzyl)(3-methylbut-2-enyl)malonate (11c)

^{1}H NMR (400 MHz, $CDCl_3$)

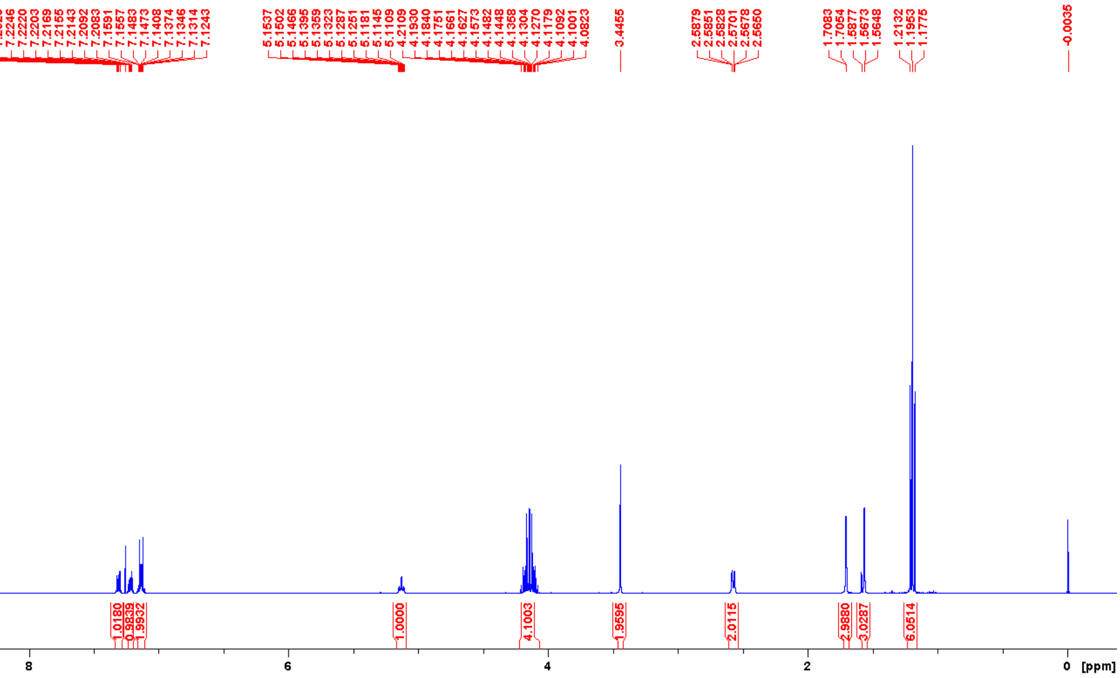

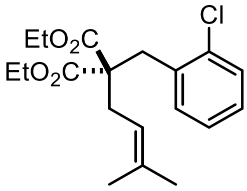

^{13}C{^{1}H} NMR (100 MHz, $CDCl_3$)

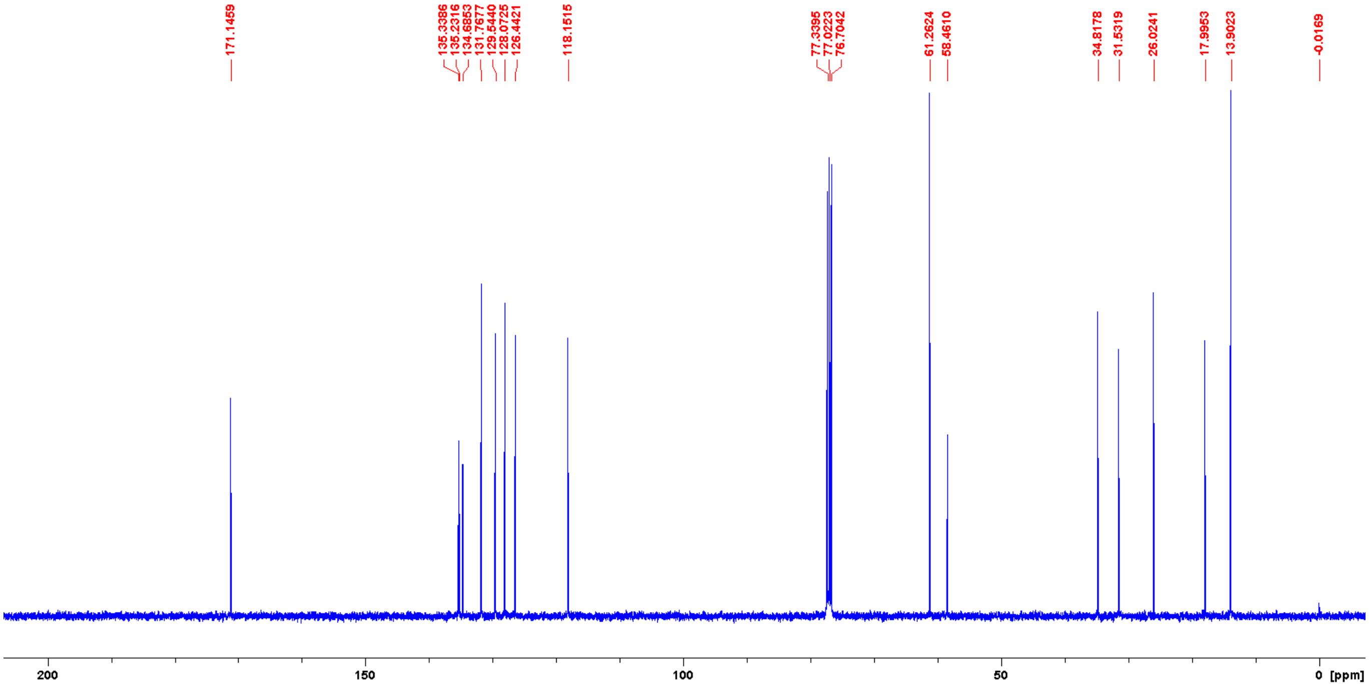

Diethyl (2-methoxybenzyl)(3-methylbut-2-enyl)malonate (11d)

^{1}H NMR (400 MHz, $CDCl_3$)

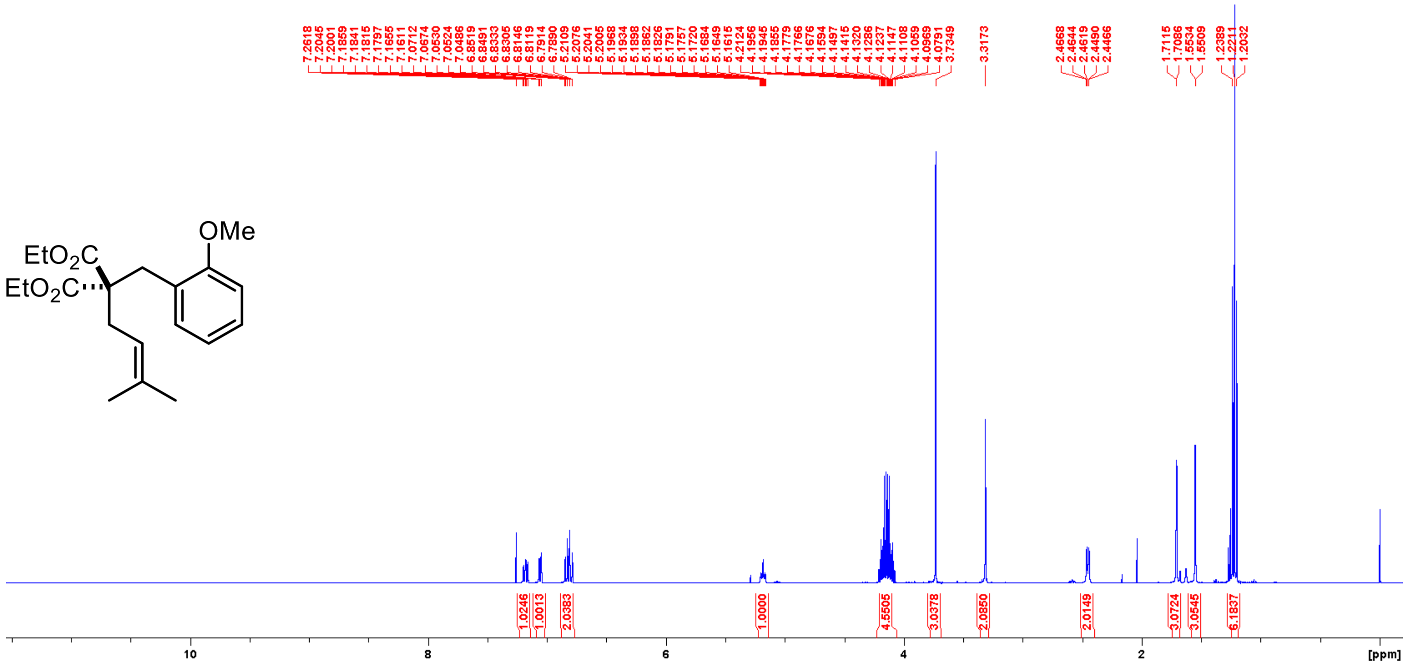

^{13}C{^{1}H} NMR (100 MHz, $CDCl_3$)

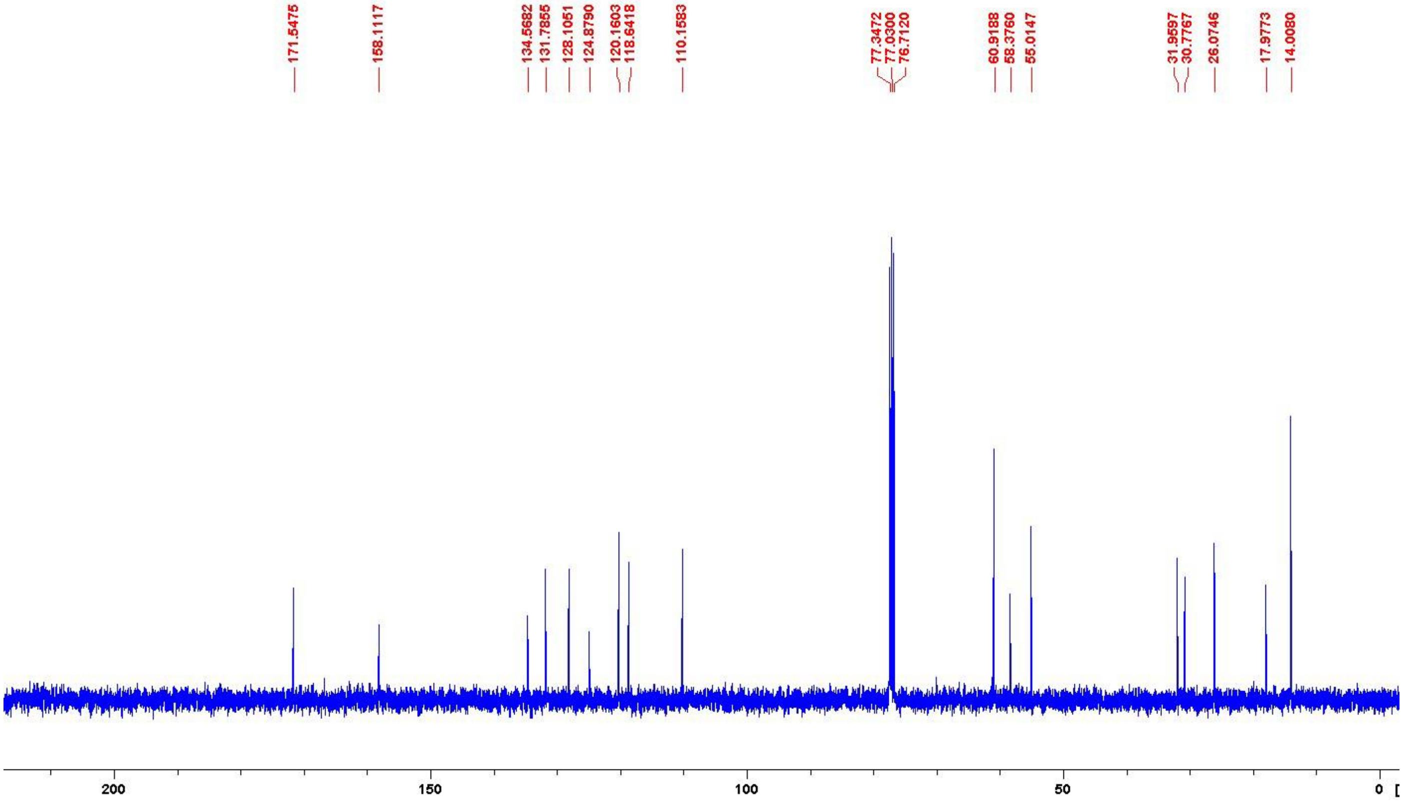

Diethyl (3-methylbenzyl)(3-methylbut-2-enyl)malonate (11e)

^{1}H NMR (400 MHz, $CDCl_3$)

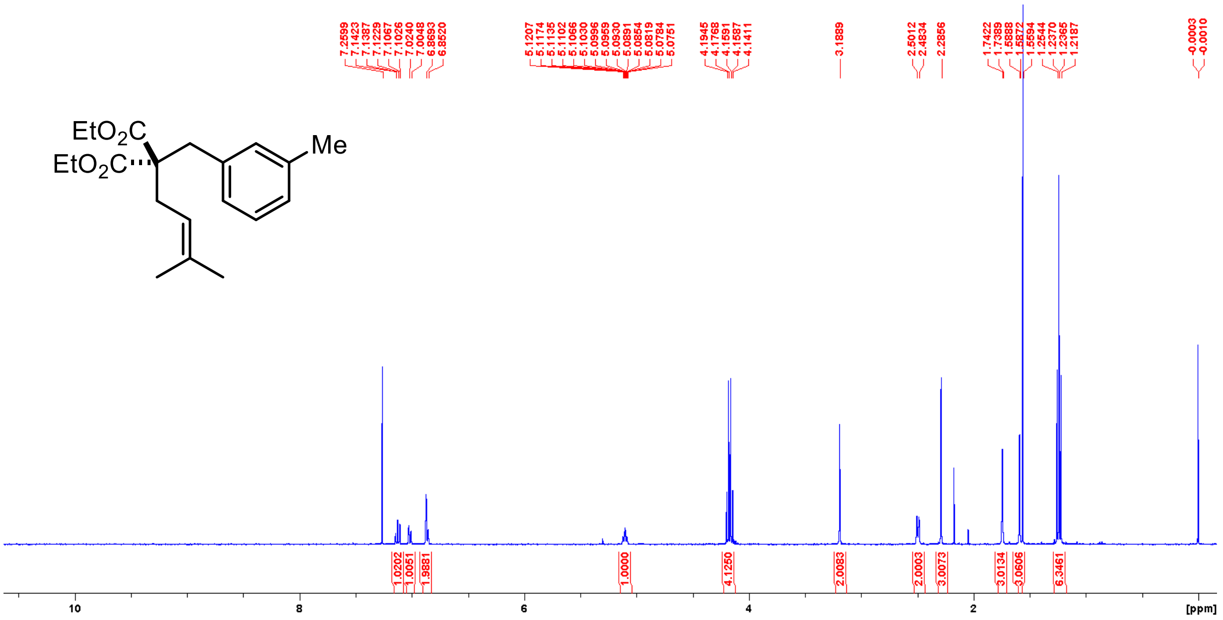

$^{13}C\{^{1}H\}$ NMR (100 MHz, $CDCl_3$)

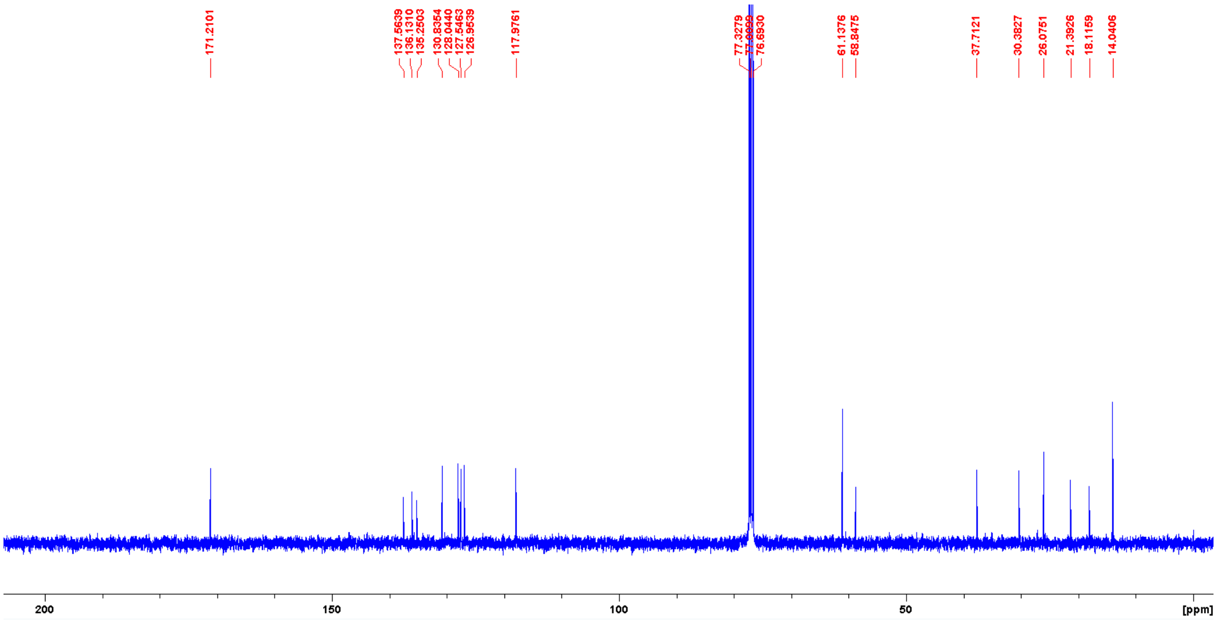

Diethyl (3-fluorobenzyl)(3-methylbut-2-enyl)malonate (11f)

^1H NMR (400 MHz, $CDCl_3$)

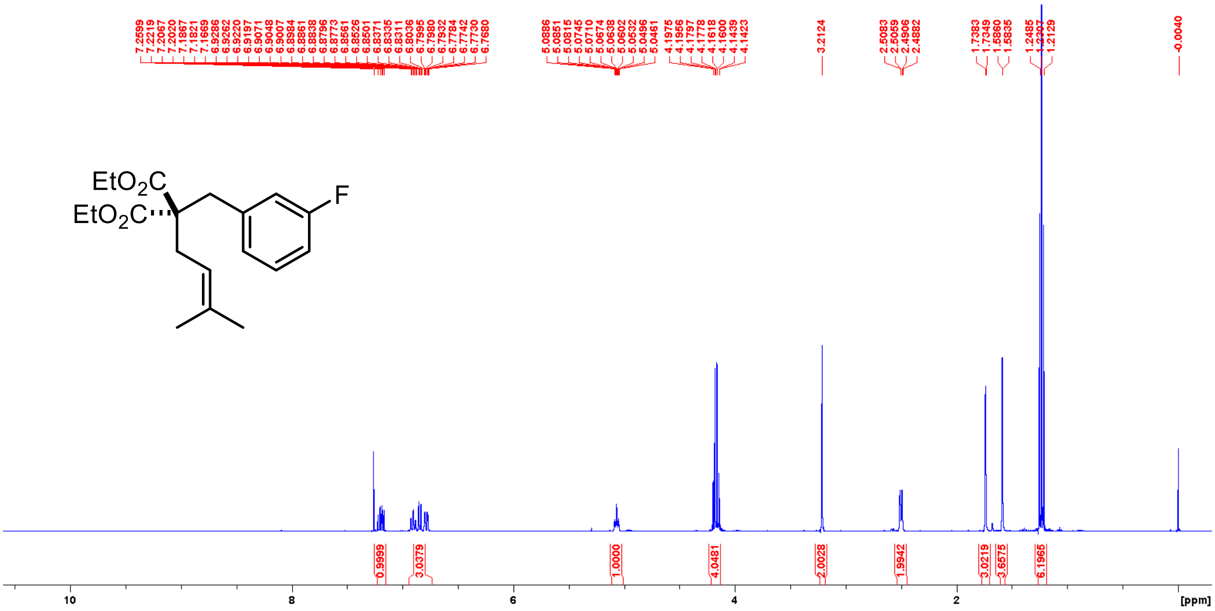

$^{13}C\{^1H\}$ NMR (100 MHz, $CDCl_3$)

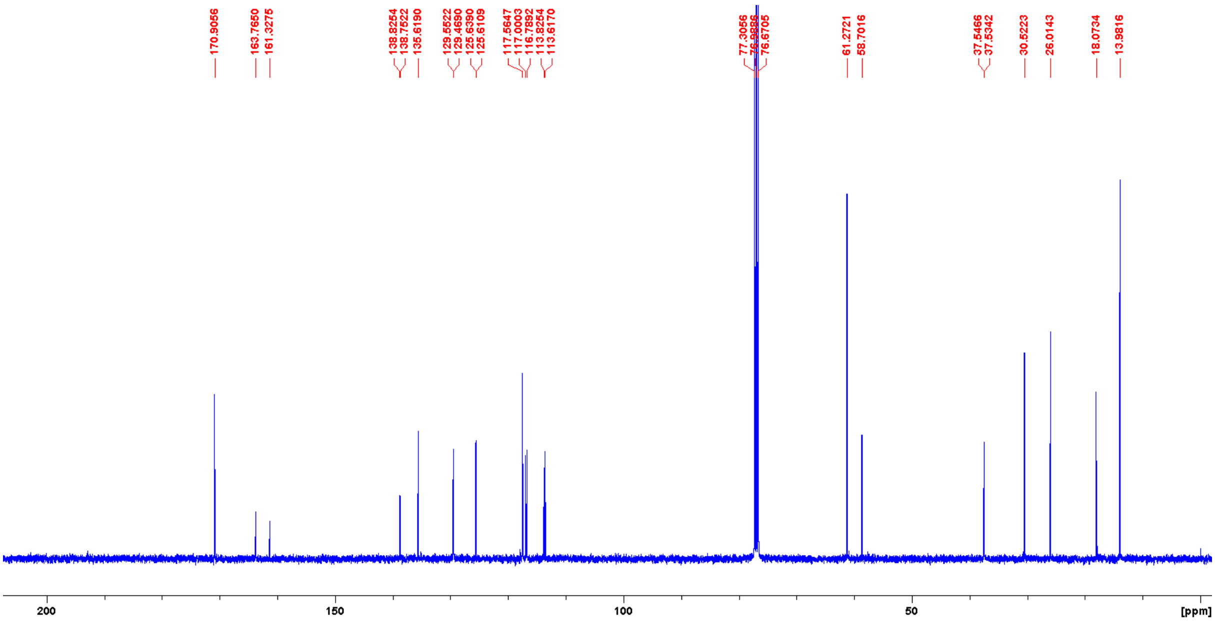

Diethyl (3-chlorobenzyl)(3-methylbut-2-enyl)malonate (11g)

^{1}H NMR (400 MHz, $CDCl_3$)

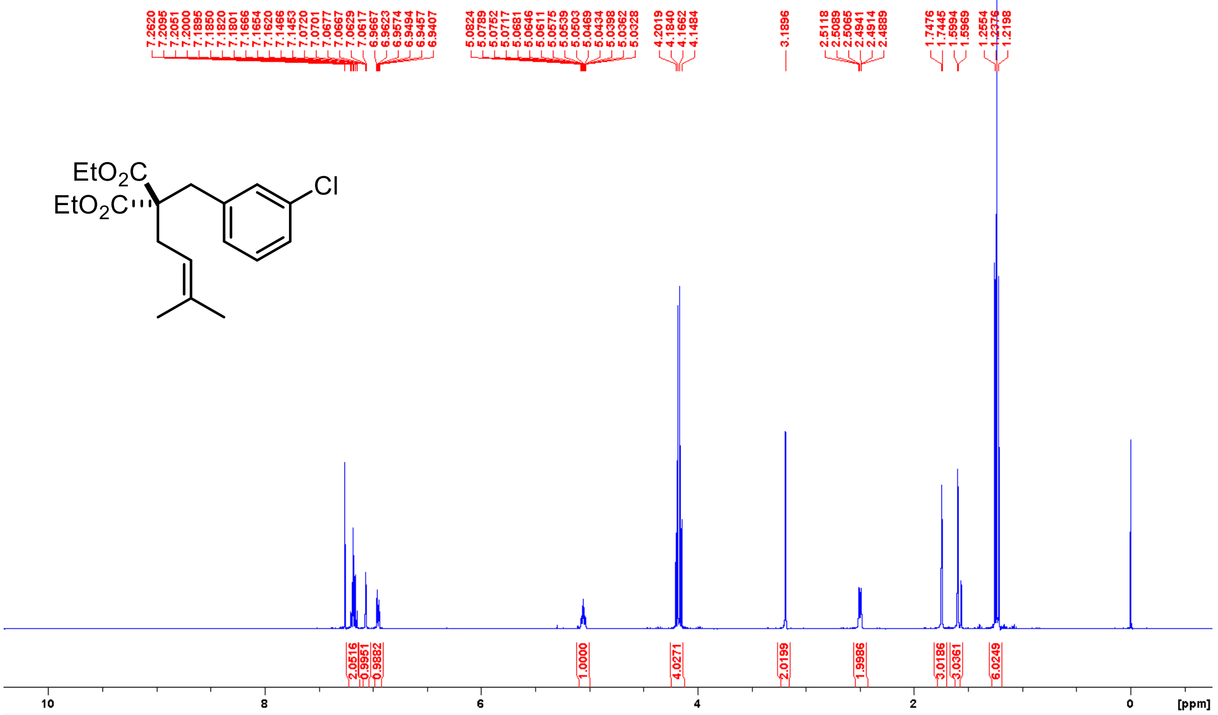

^{13}C{^{1}H} NMR (100 MHz, $CDCl_3$)

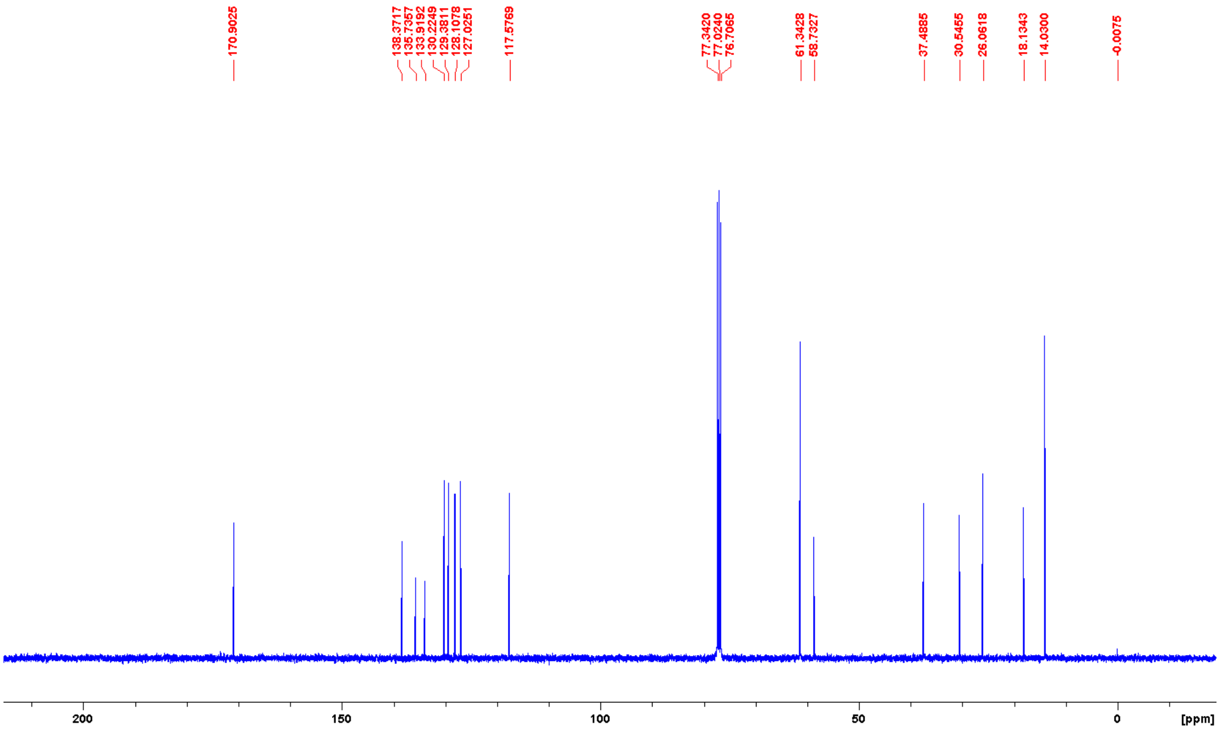

Diethyl (3-bromobenzyl)(3-methylbut-2-enyl)malonate (11h)

^{1}H NMR (400 MHz, $CDCl_3$)

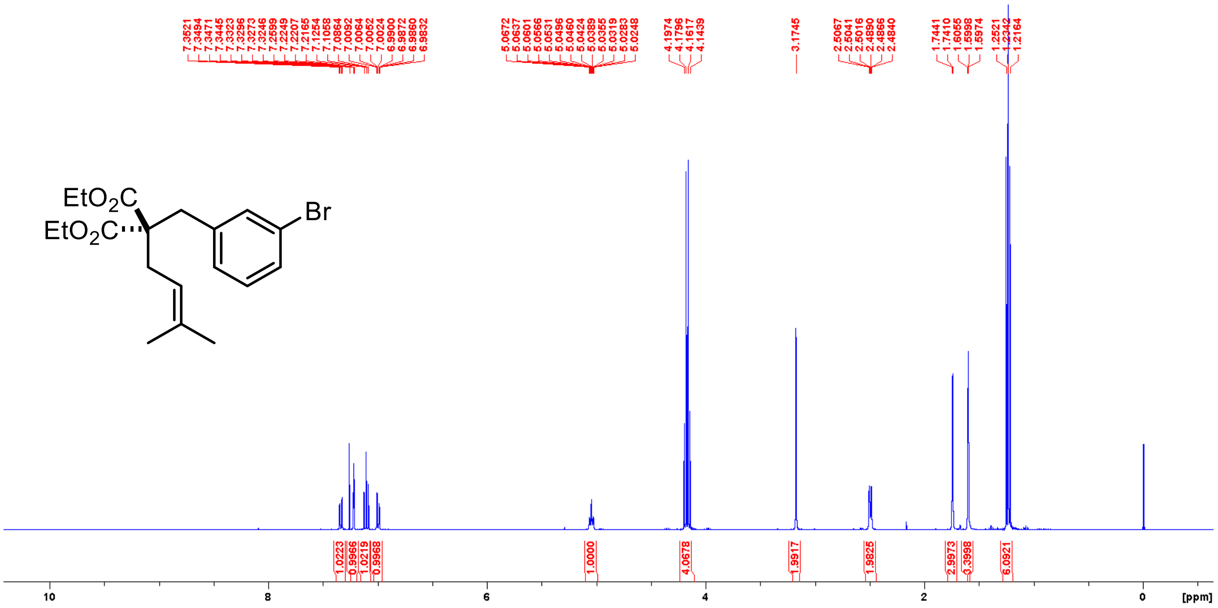

^{13}C{^{1}H} NMR (100 MHz, $CDCl_3$)

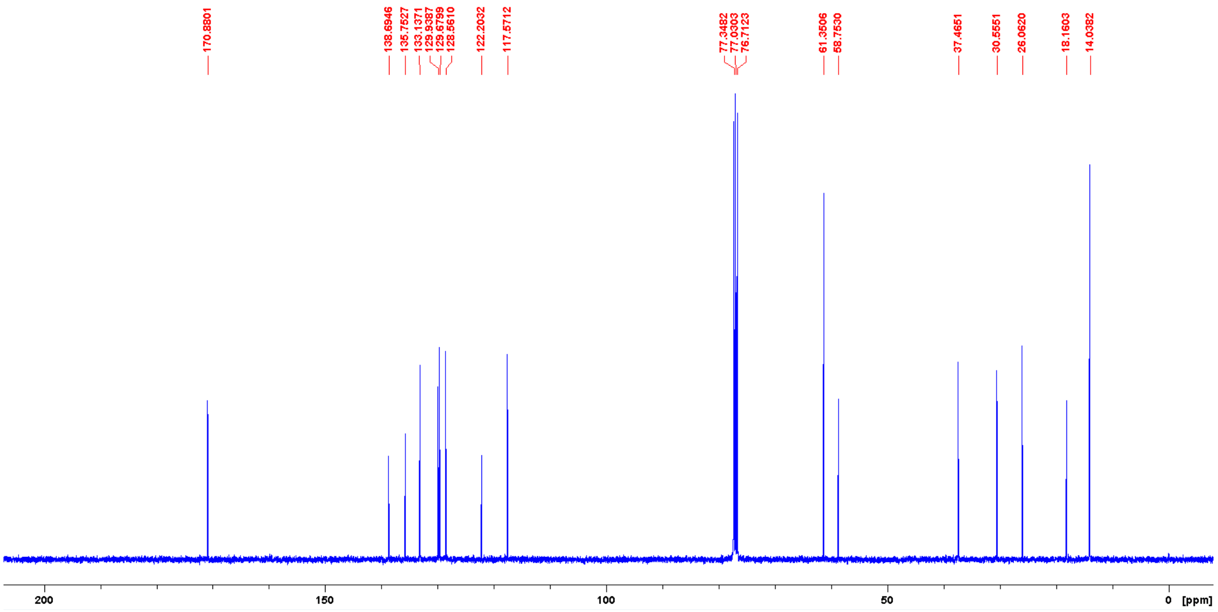

Diethyl (3-methoxybenzyl)(3-methylbut-2-enyl)malonate (11i)

^{1}H NMR (400 MHz, $CDCl_3$)

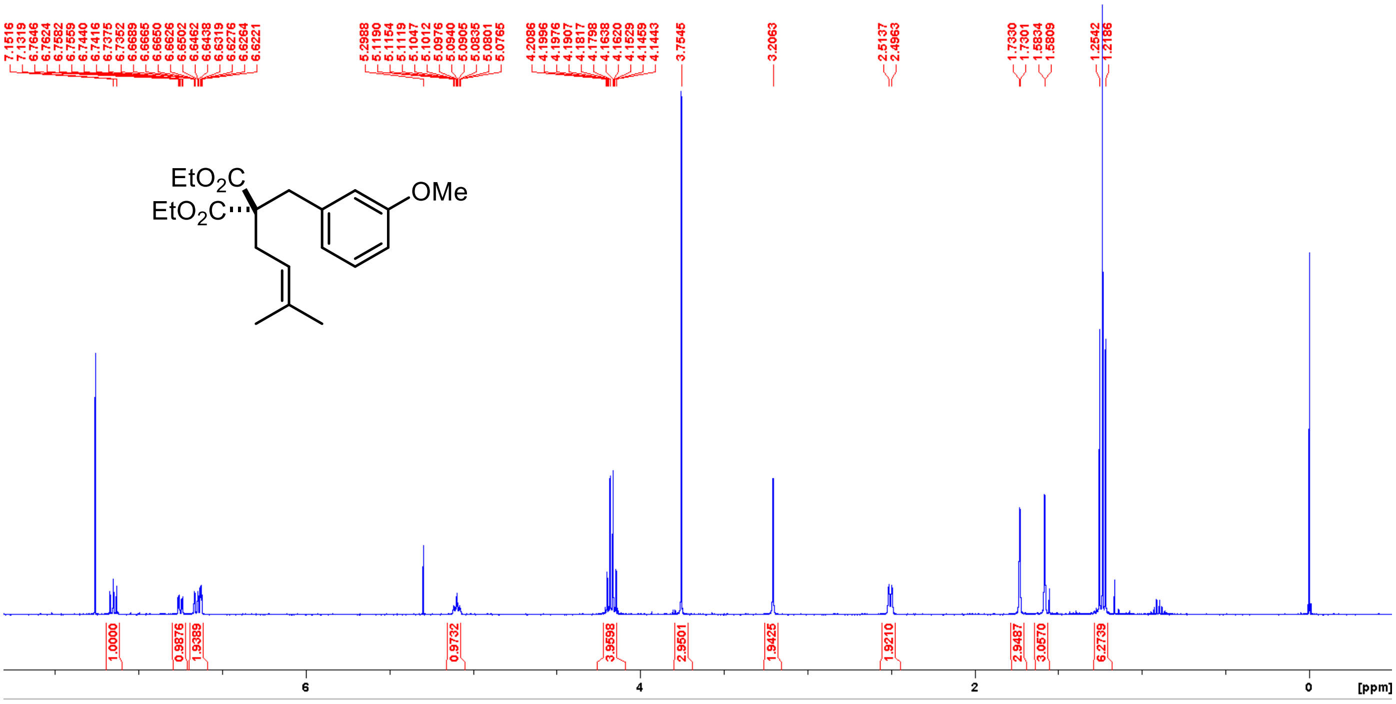

^{13}C{^{1}H} NMR (100 MHz, $CDCl_3$)

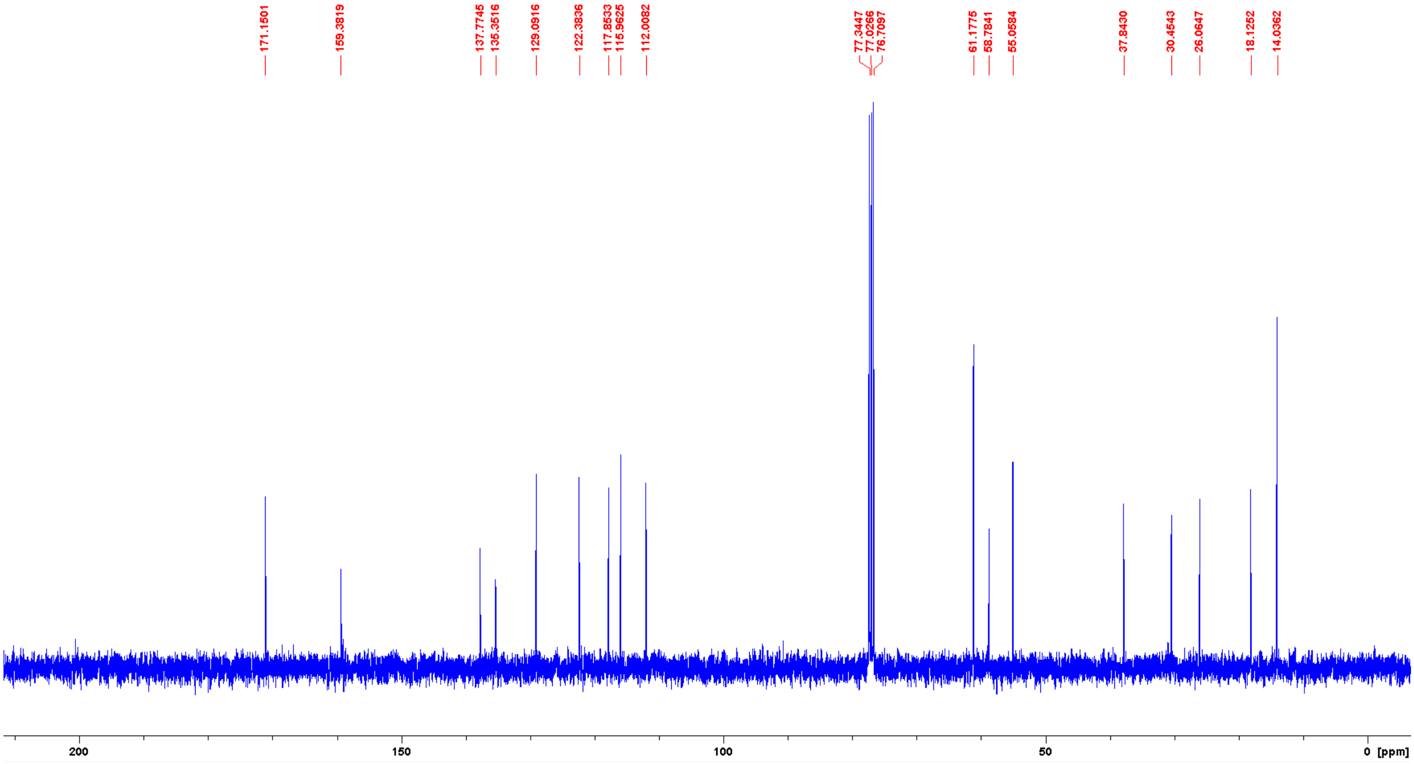

Diethyl (4-bromobenzyl)(3-methylbut-2-enyl)malonate (11j)

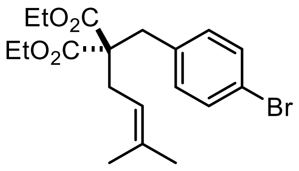

^{1}H NMR (400 MHz, CDCl$_3$)

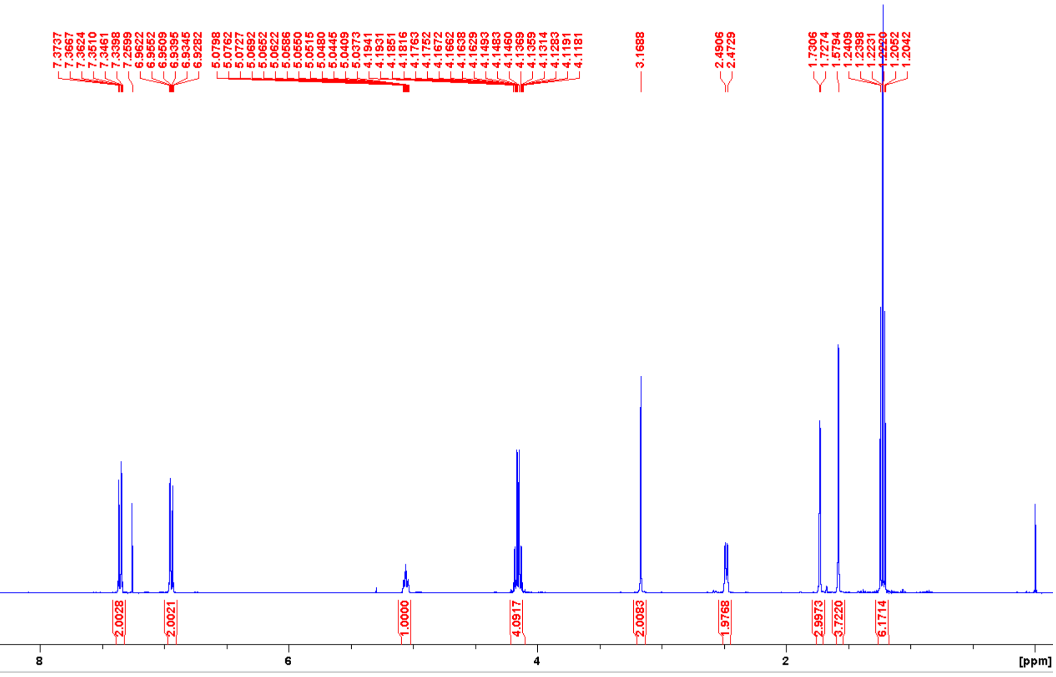

^{13}C{^{1}H} NMR (100 MHz, CDCl$_3$)

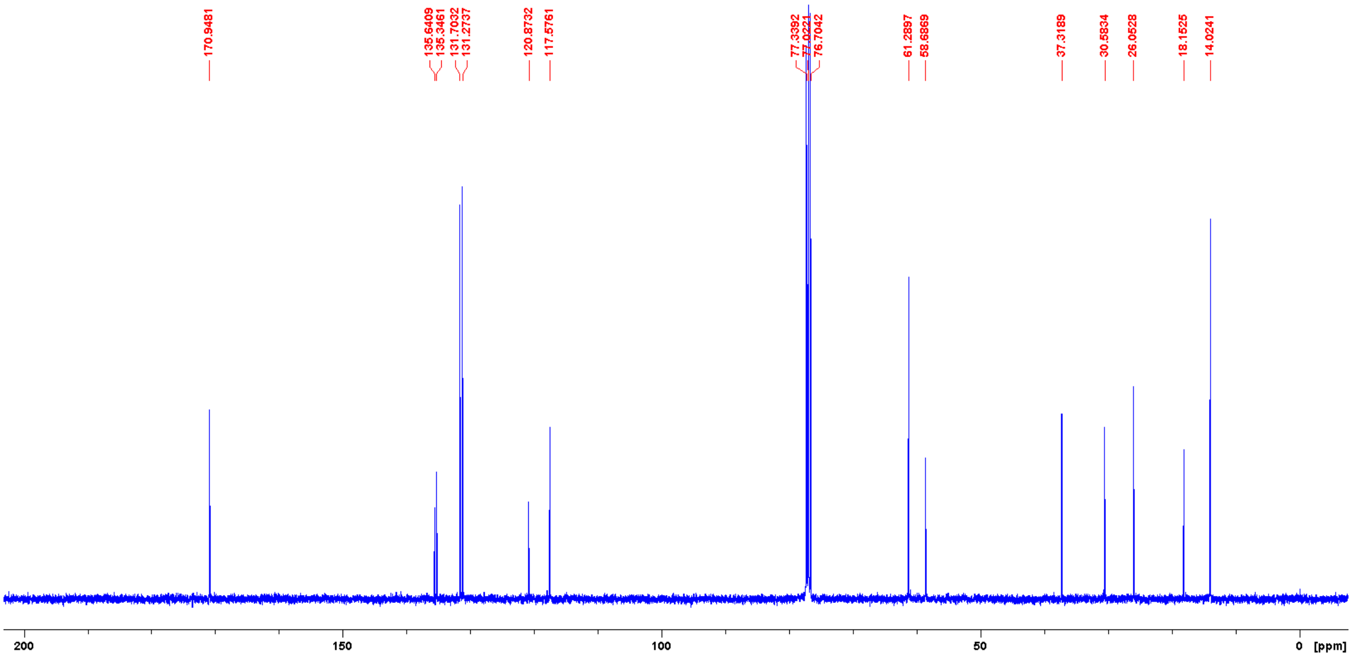

Diethyl (4-methoxycarbonylbenzyl)(3-methylbut-2-enyl)malonate (11k)

^{1}H NMR (400 MHz, $CDCl_3$)

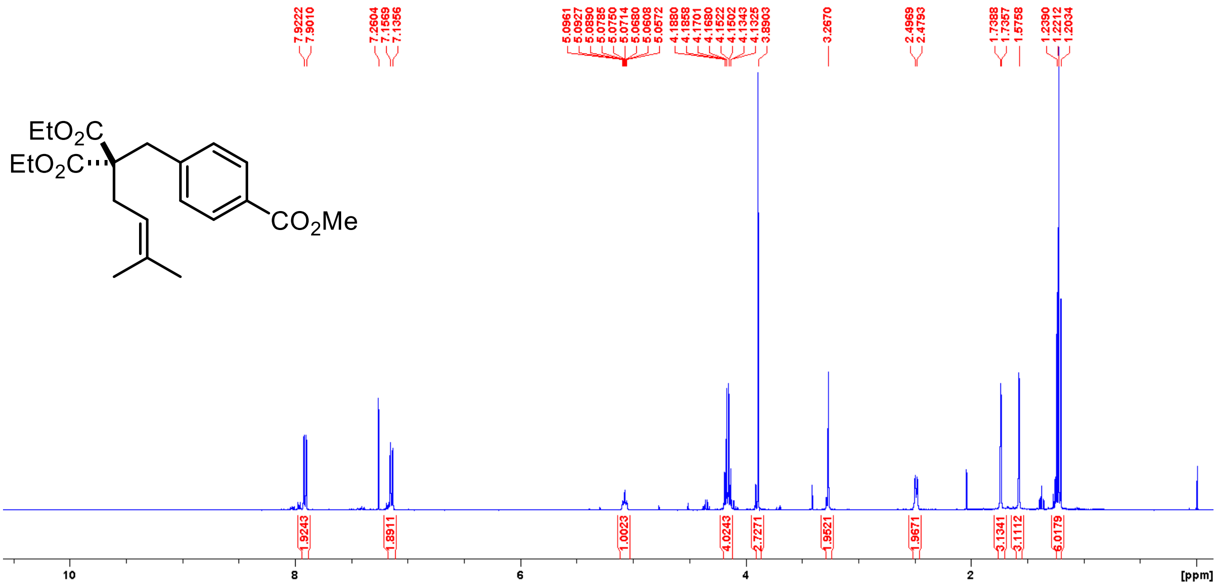

$^{13}C\{^{1}H\}$ NMR (100 MHz, $CDCl_3$)

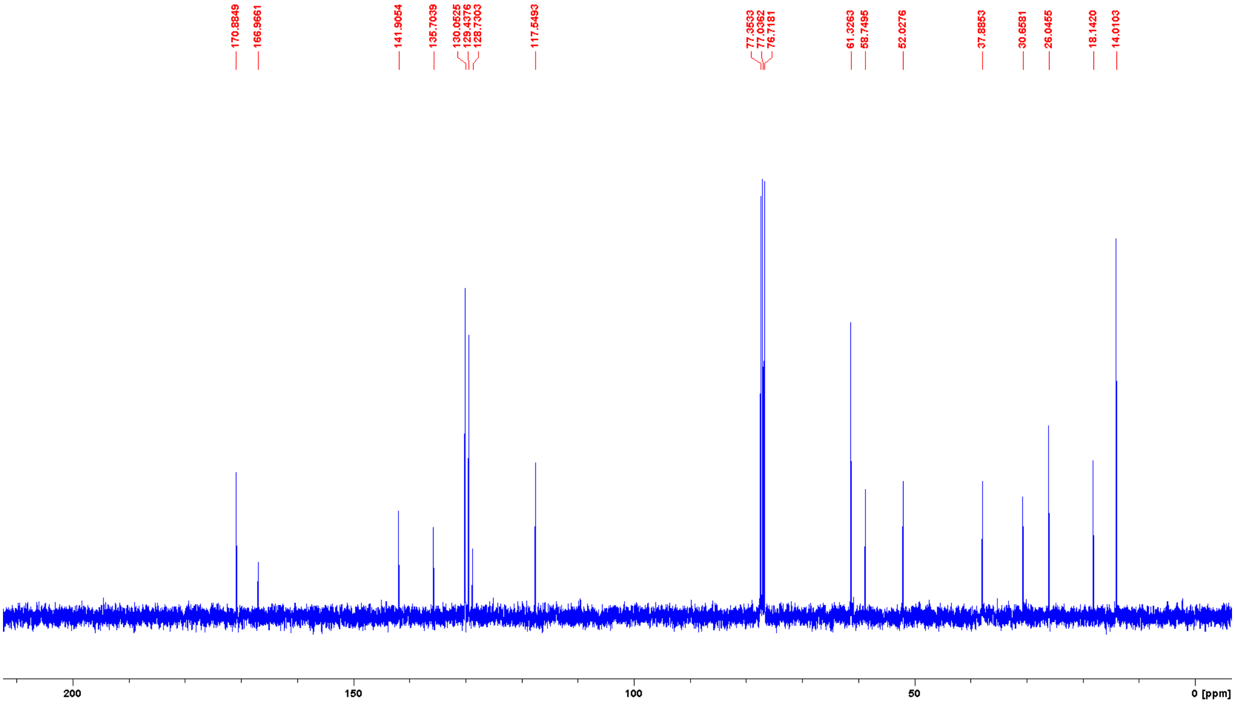

Diethyl (3-cyanobenzyl)(3-methylbut-2-enyl)malonate (11l)

^{1}H NMR (400 MHz, $CDCl_3$)

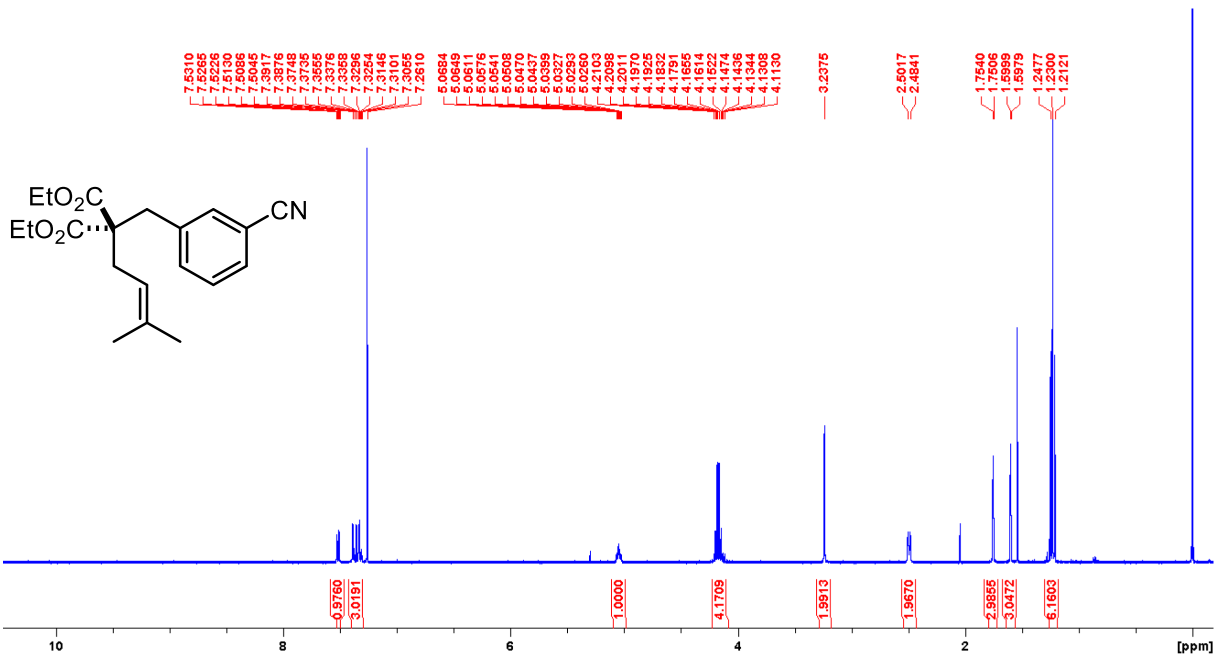

^{13}C{^{1}H} NMR (100 MHz, $CDCl_3$)

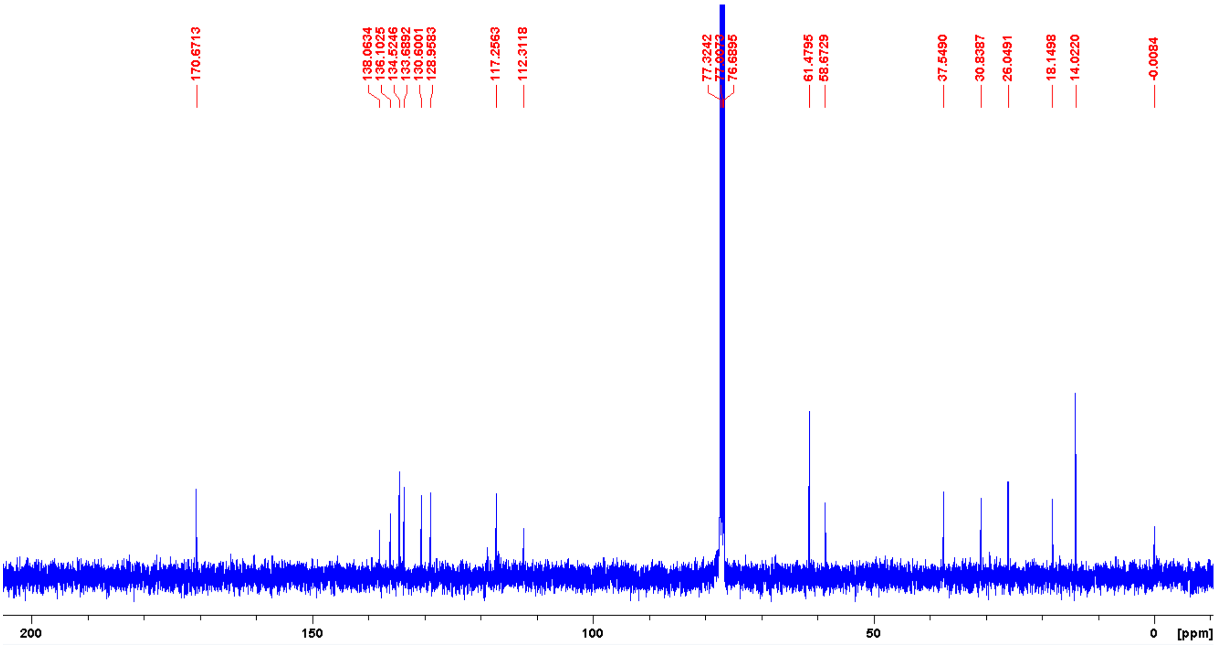

Diethyl (4-cyanobenzyl)(3-methylbut-2-enyl)malonate (11m)

^{1}H NMR (400 MHz, $CDCl_3$)

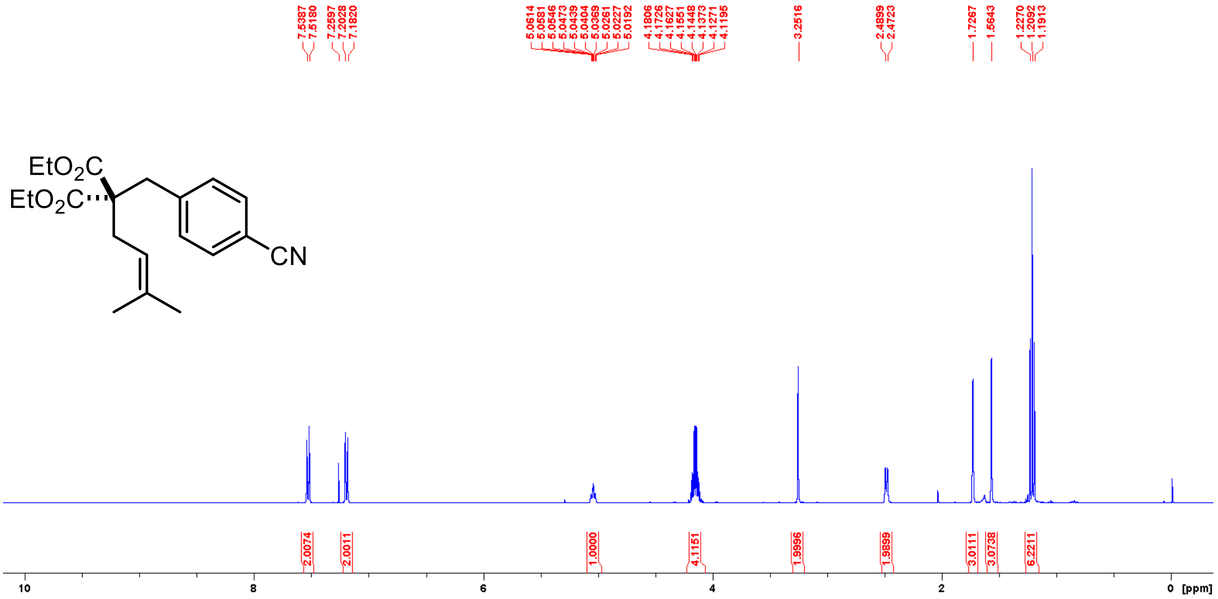

^{13}C{^{1}H} NMR (100 MHz, $CDCl_3$)

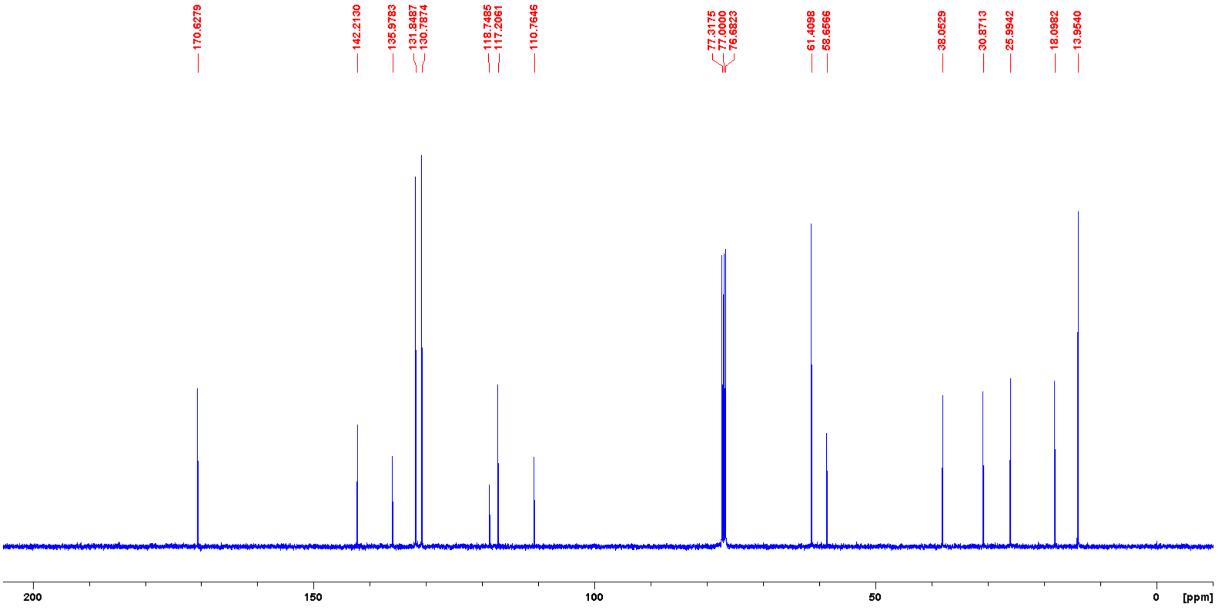

Diethyl (4-nitrobenzyl)(3-methylbut-2-enyl)malonate (11n)

^{1}H NMR (400 MHz, $CDCl_3$)

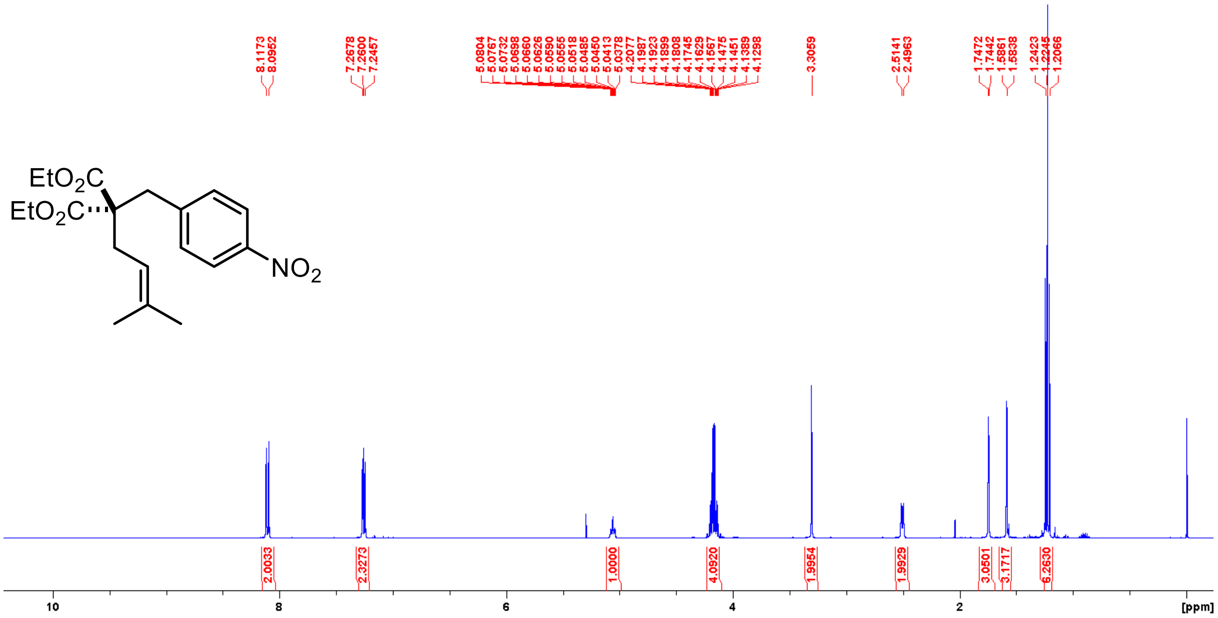

$^{13}C\{^{1}H\}$ NMR (100 MHz, $CDCl_3$)

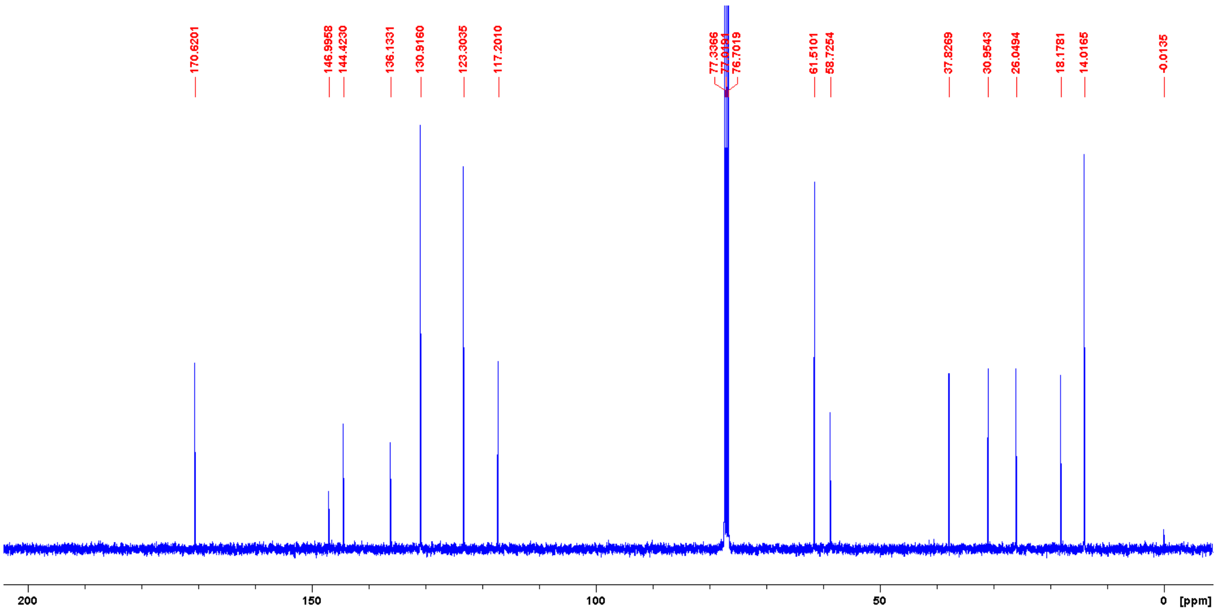

3,3-Bis(ethoxycarbonyl)-5-methyl-1-(2,4,6-trimethoxyphenyl)-1,2,3,4-tetrahydronaphthalene (12a)

^{1}H NMR (400 MHz, $CDCl_3$)

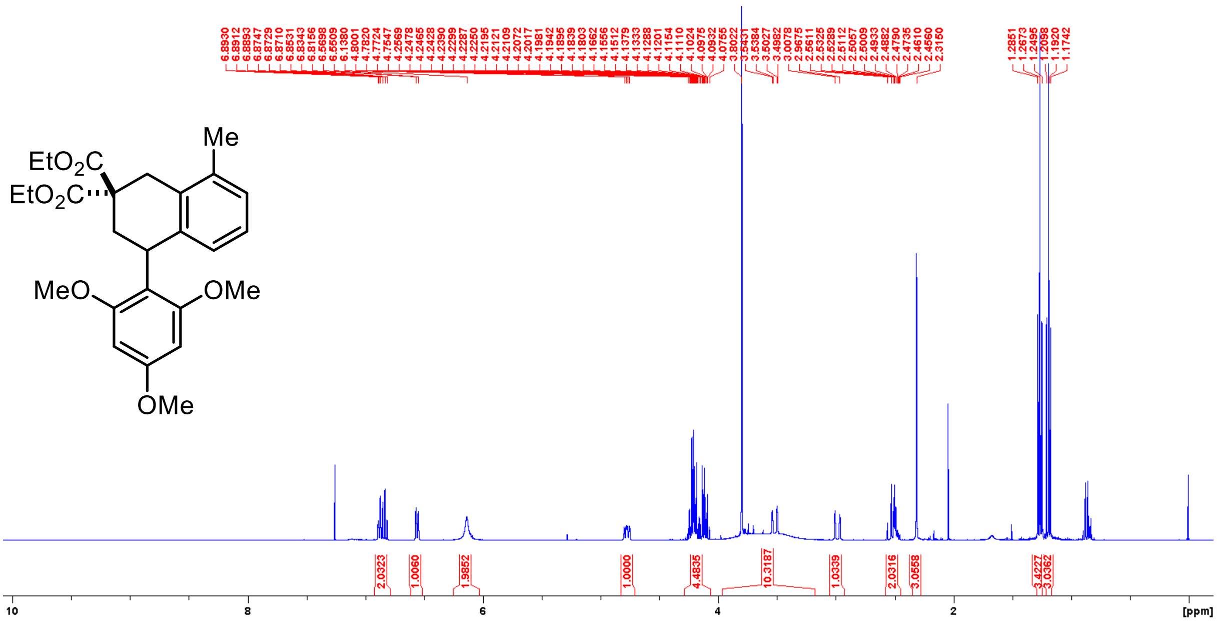

^{13}C{^{1}H} NMR (100 MHz, $CDCl_3$)

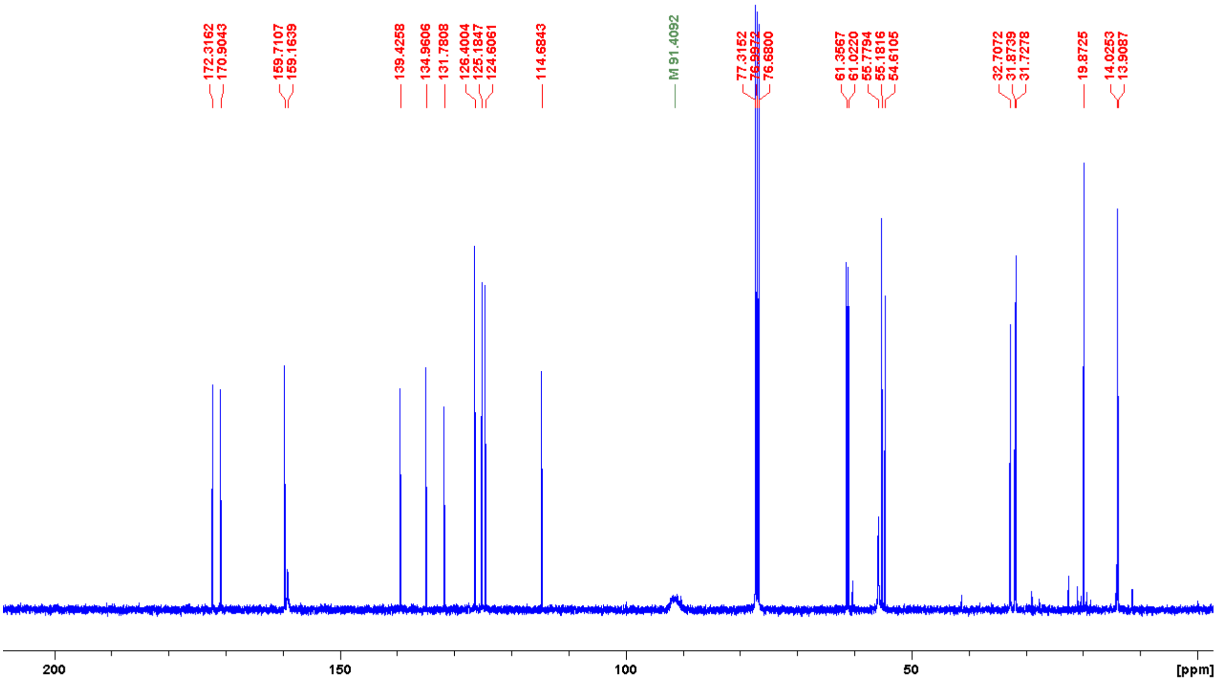

3,3-Bis(ethoxycarbonyl)-5-fluoro-1-(2,4,6-trimethoxyphenyl)-1,2,3,4-tetrahydronaphthalene (12b)

^{1}H NMR (400 MHz, $CDCl_3$)

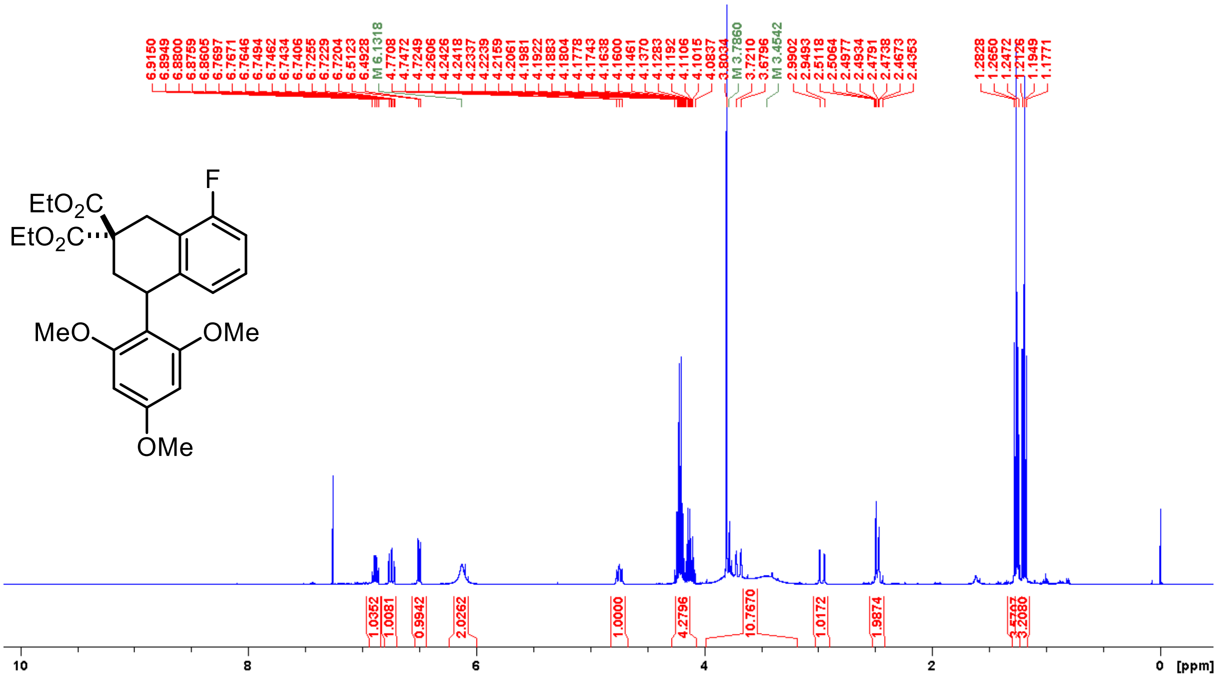

$^{13}C\{^{1}H\}$ NMR (100 MHz, $CDCl_3$)

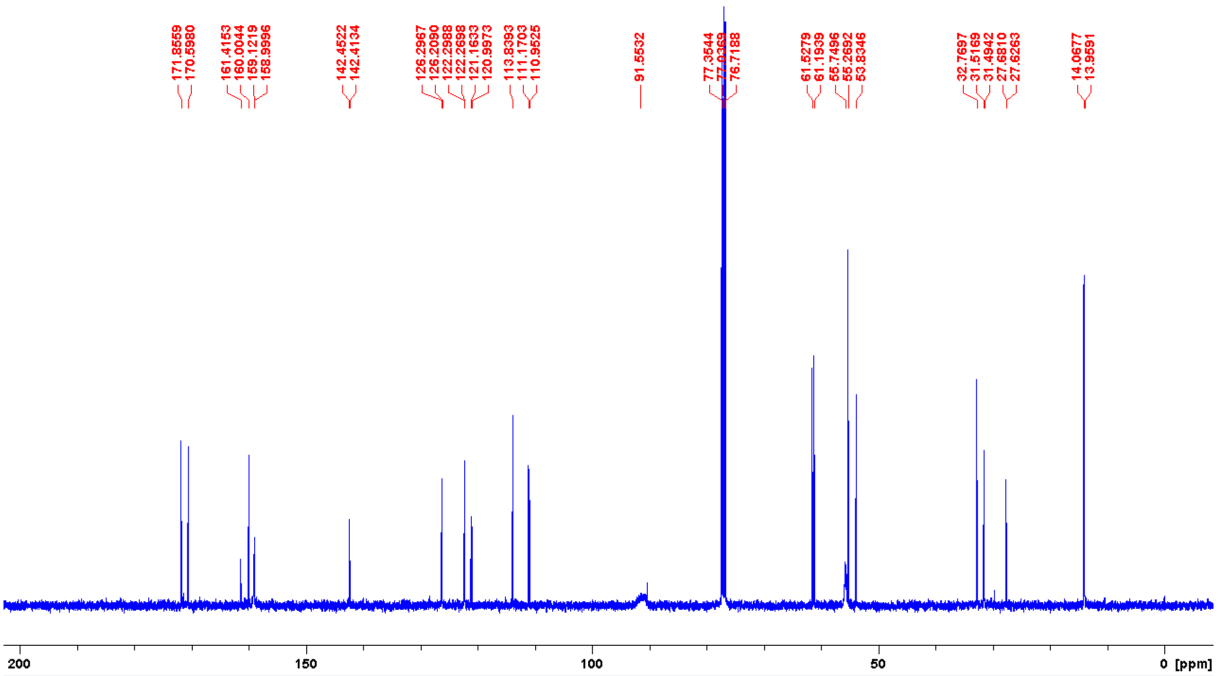

3,3-Bis(ethoxycarbonyl)-5-chloro-1-(2,4,6-trimethoxyphenyl)-1,2,3,4-tetrahydronaphthalene (12c)

^{1}H NMR (400 MHz, $CDCl_3$)

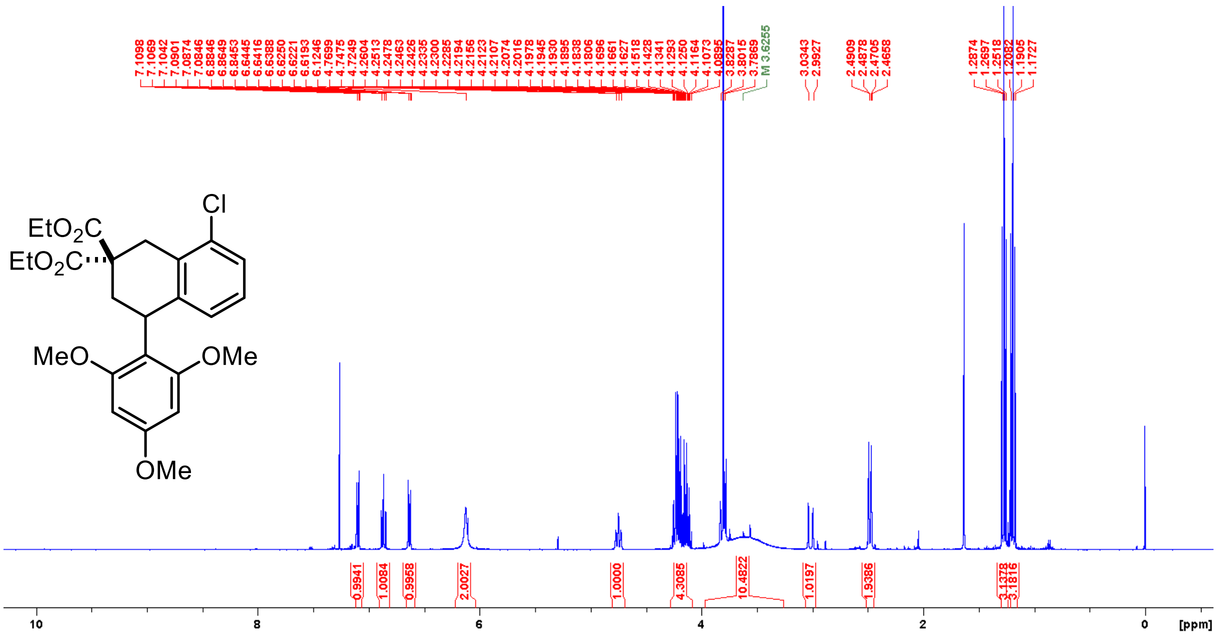

^{13}C{^{1}H} NMR (100 MHz, $CDCl_3$)

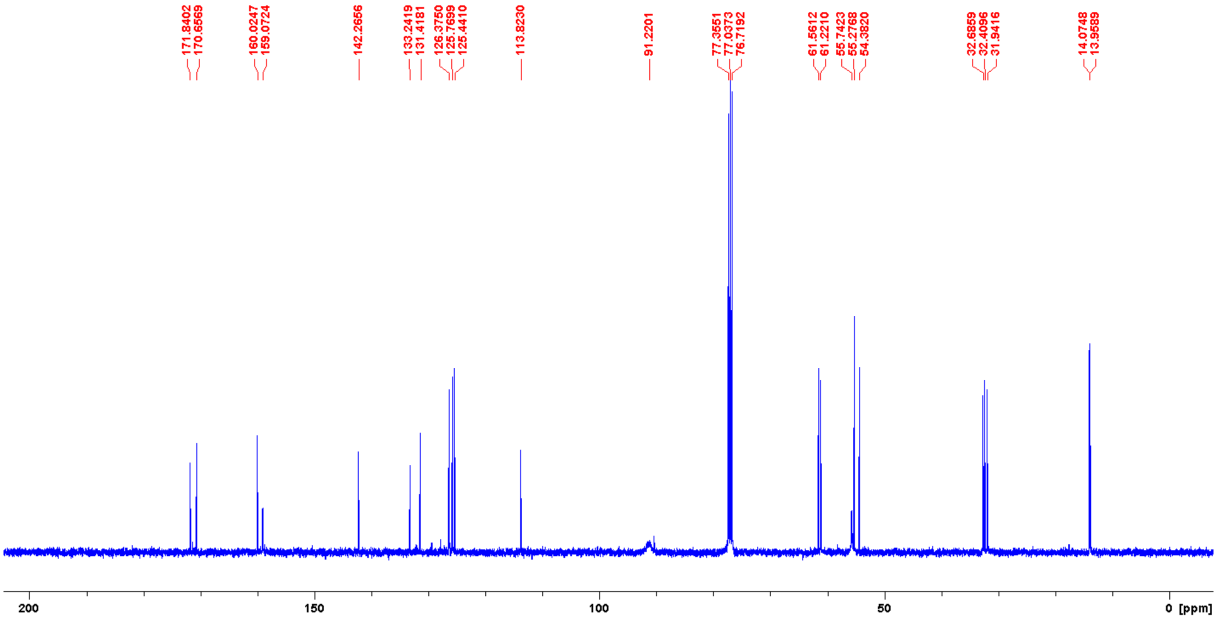

3,3-Bis(ethoxycarbonyl)-5-methoxy-1-(2,4,6-trimethoxyphenyl)-1,2,3,4-tetrahydronaphthalene (12d)

^{1}H NMR (400 MHz, $CDCl_3$)

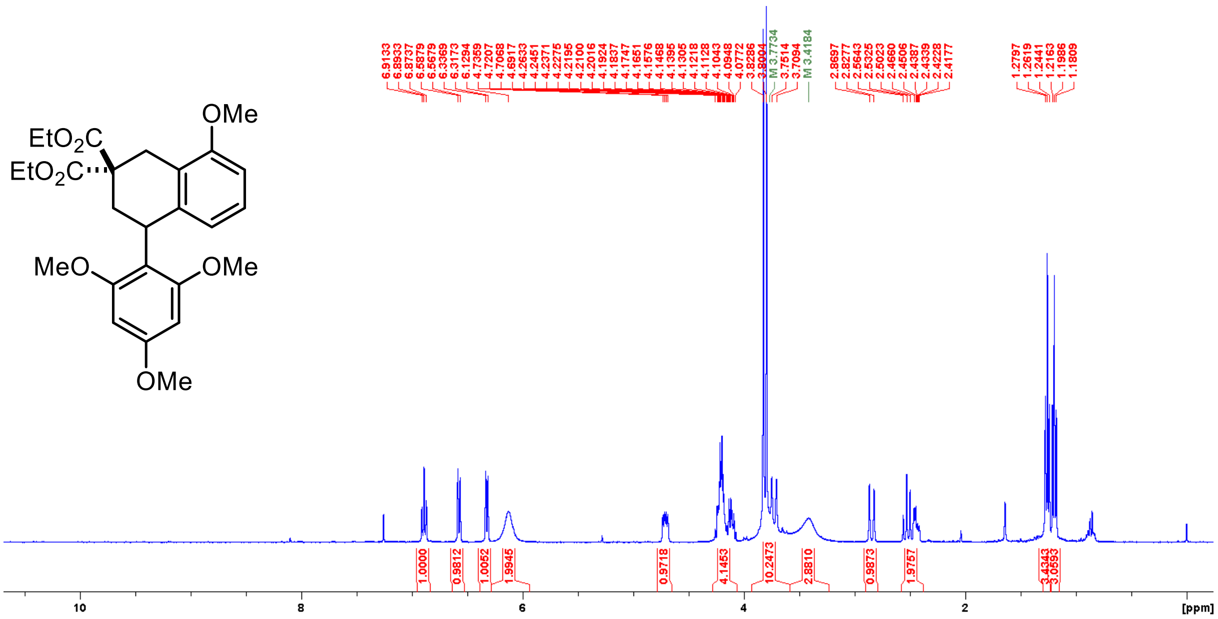

$^{13}C\{^{1}H\}$ NMR (100 MHz, $CDCl_3$)

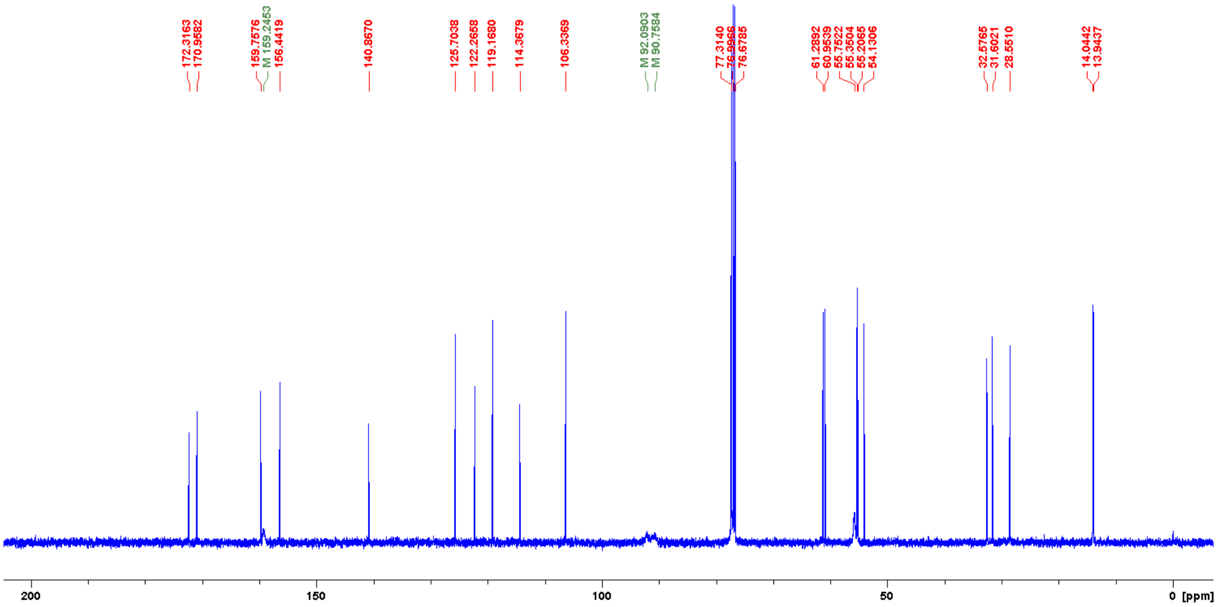

3,3-Bis(ethoxycarbonyl)-6-methyl-1-(2,4,6-trimethoxyphenyl)-1,2,3,4-tetrahydronaphthalene (12e)

^{1}H NMR (400 MHz, $CDCl_3$)

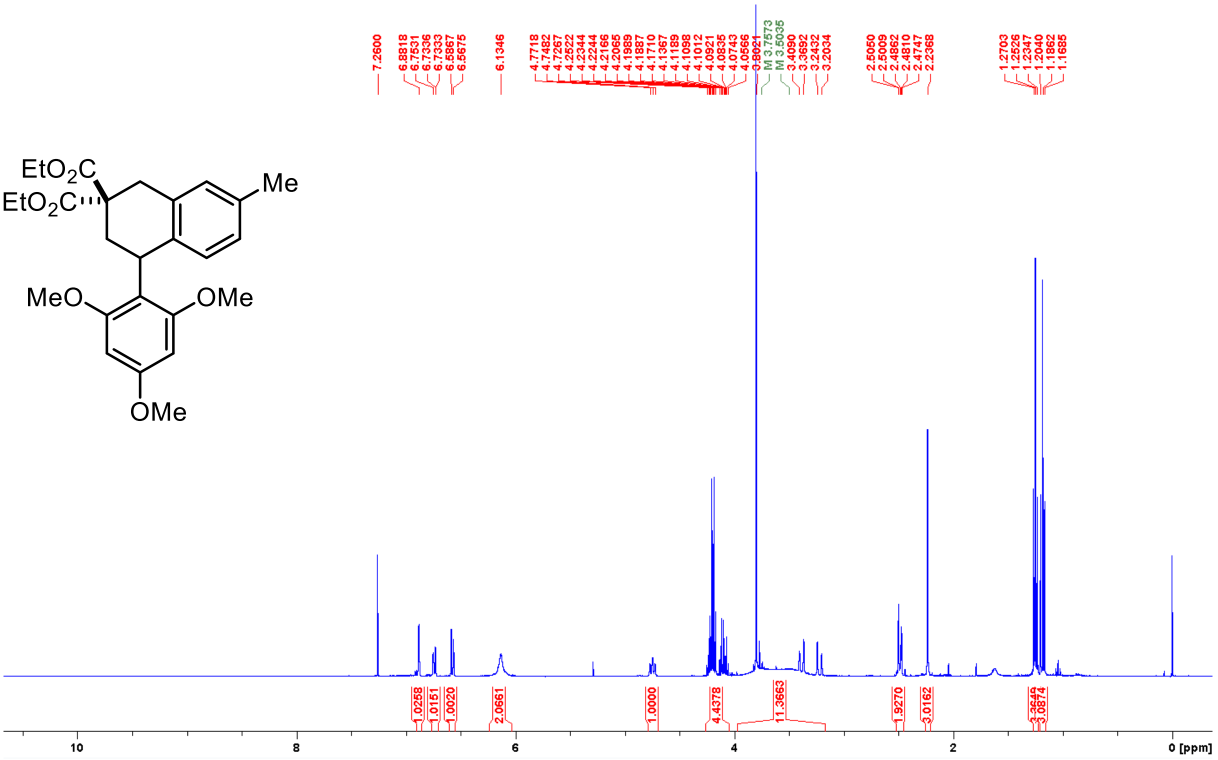

$^{13}C\{^{1}H\}$ NMR (100 MHz, $CDCl_3$)

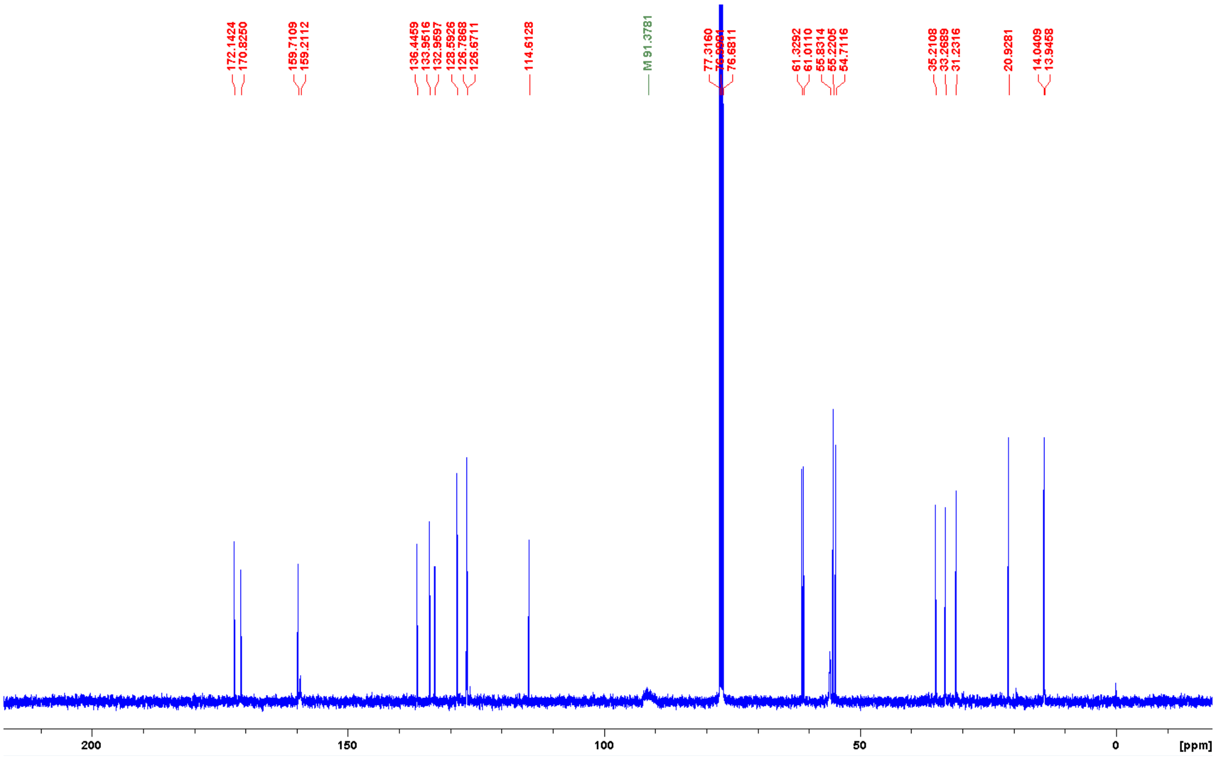

3,3-Bis(ethoxycarbonyl)-6-fluoro-1-(2,4,6-trimethoxyphenyl)-1,2,3,4-tetrahydronaphthalene (12f)

^{1}H NMR (400 MHz, $CDCl_3$)

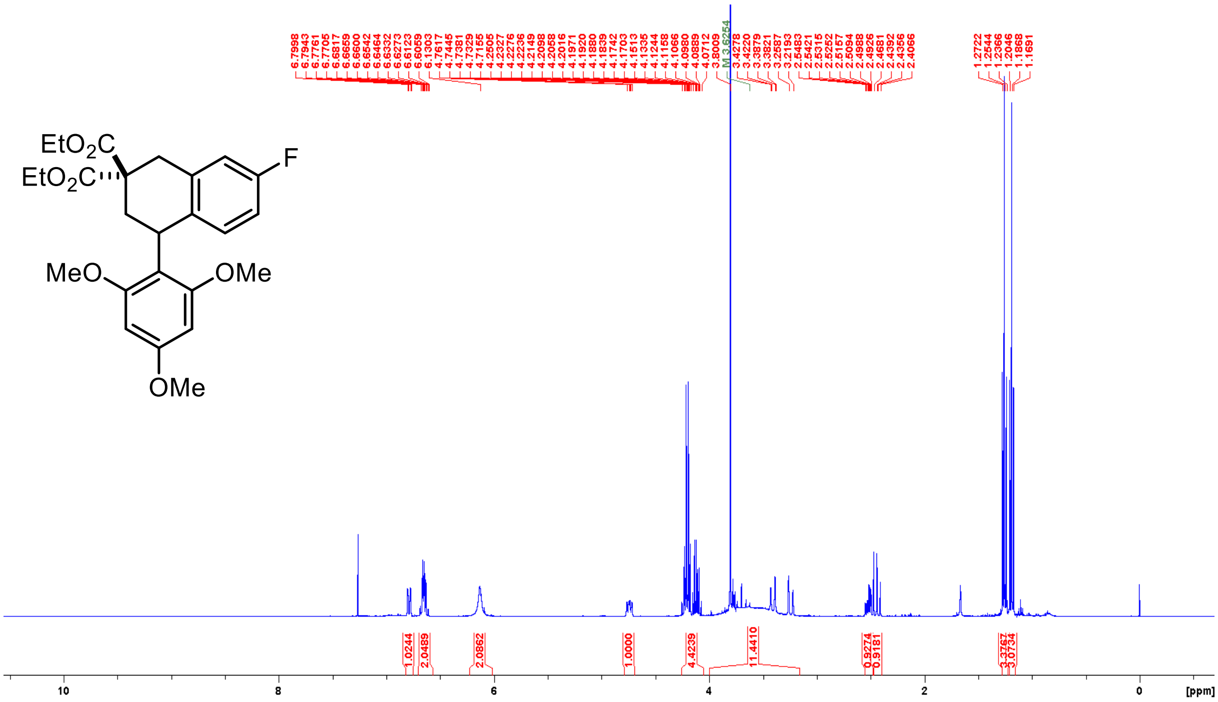

$^{13}C\{^{1}H\}$ NMR (100 MHz, $CDCl_3$)

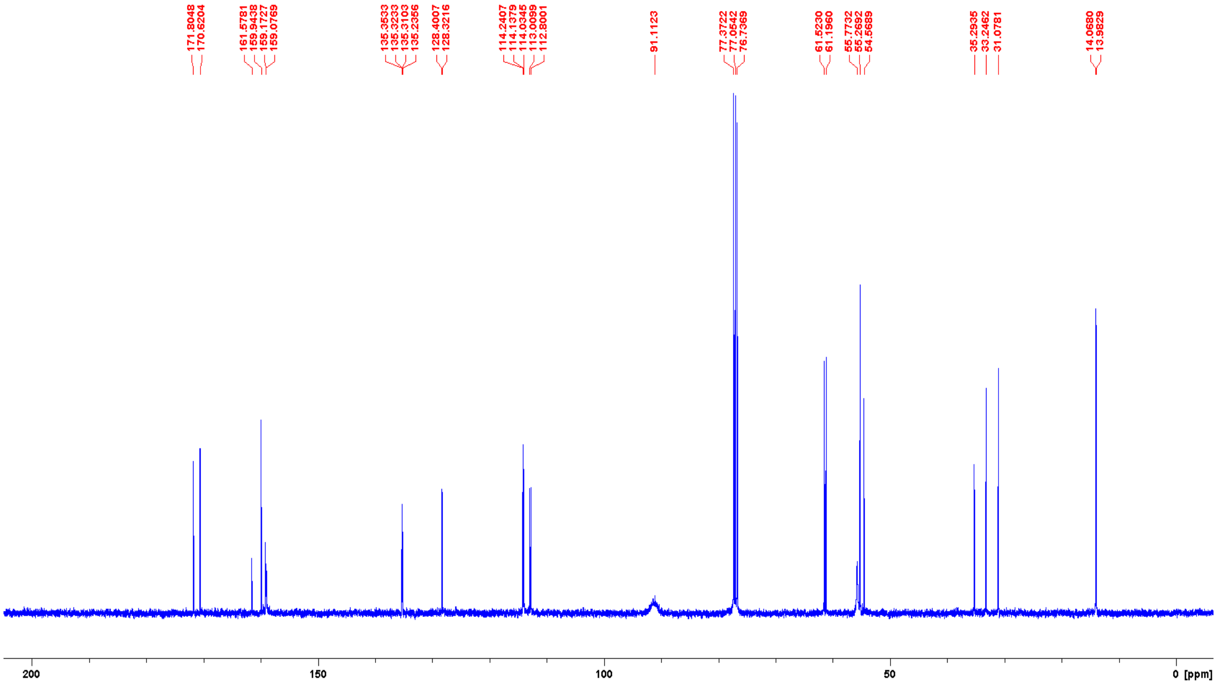

3,3-Bis(ethoxycarbonyl)-6-chloro-1-(2,4,6-trimethoxyphenyl)-1,2,3,4-tetrahydronaphthalene (12g)

^{1}H NMR (400 MHz, $CDCl_3$)

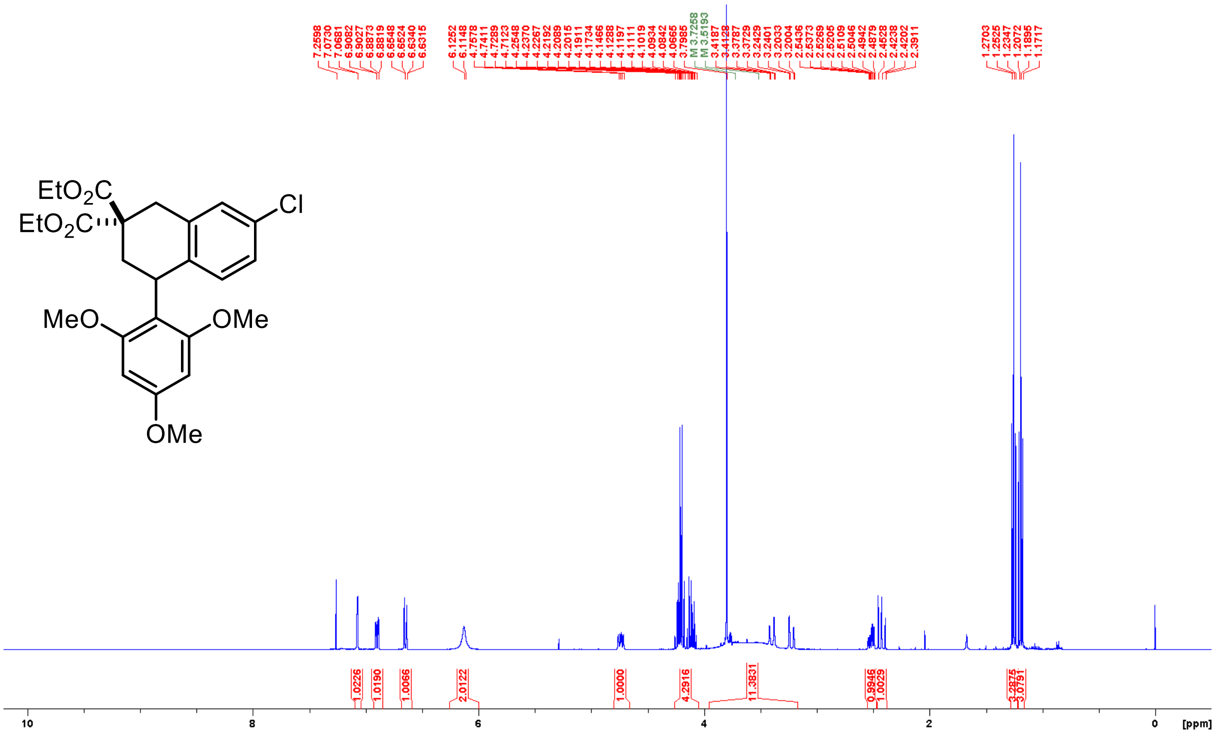

$^{13}C\{^1H\}$ NMR (100 MHz, $CDCl_3$)

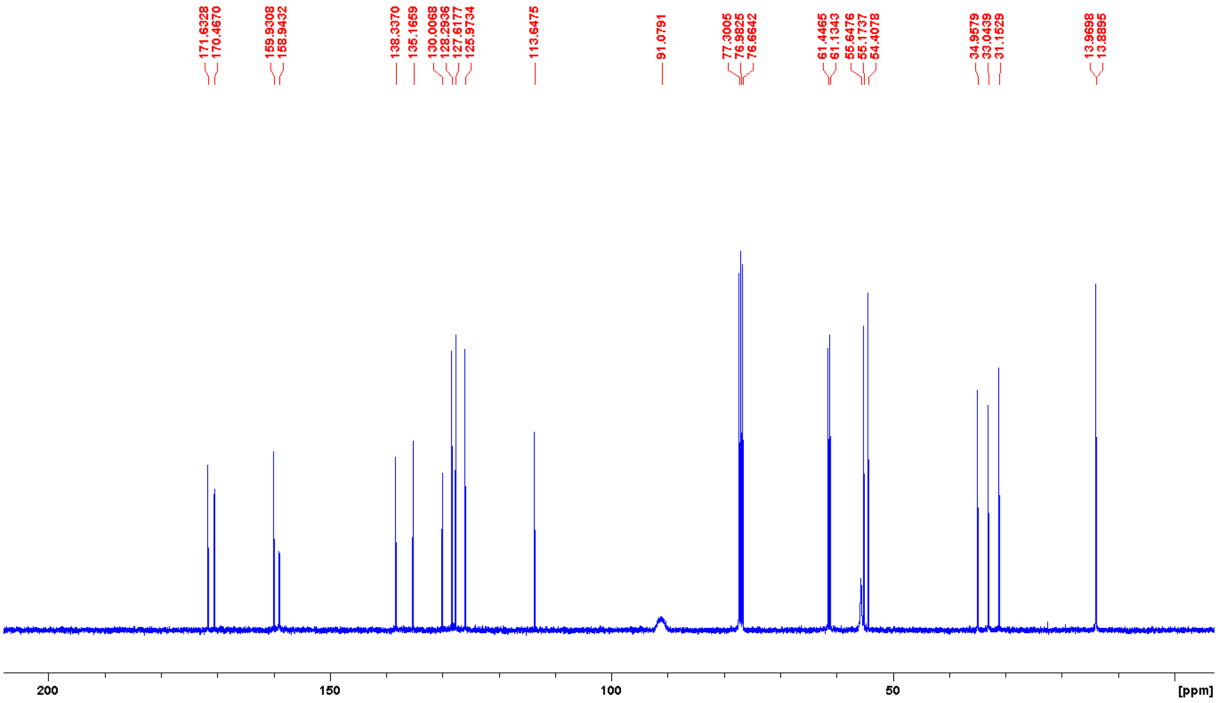

3,3-Bis(ethoxycarbonyl)-6-bromo-1-(2,4,6-trimethoxyphenyl)-1,2,3,4-tetrahydronaphthalene (12h)

^{1}H NMR (400 MHz, $CDCl_3$)

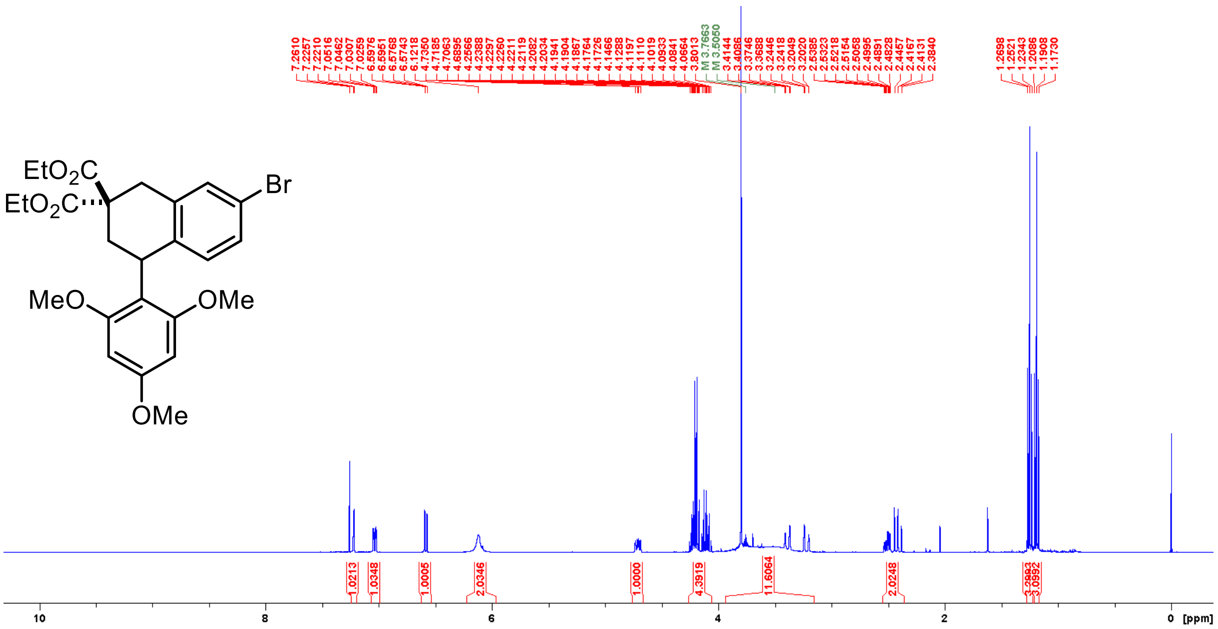

$^{13}C\{^{1}H\}$ NMR (100 MHz, $CDCl_3$)

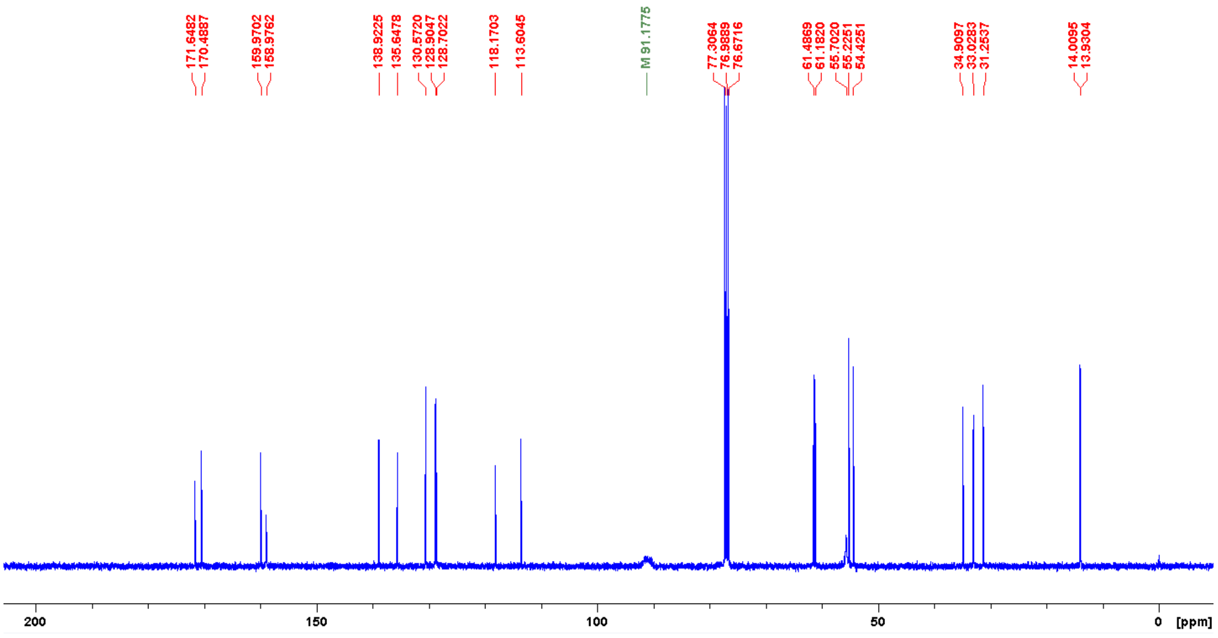

3,3-Bis(ethoxycarbonyl)-6-methoxy-1-(2,4,6-trimethoxyphenyl)-1,2,3,4-tetrahydronaphthalene (12i)

^{1}H NMR (400 MHz, $CDCl_3$)

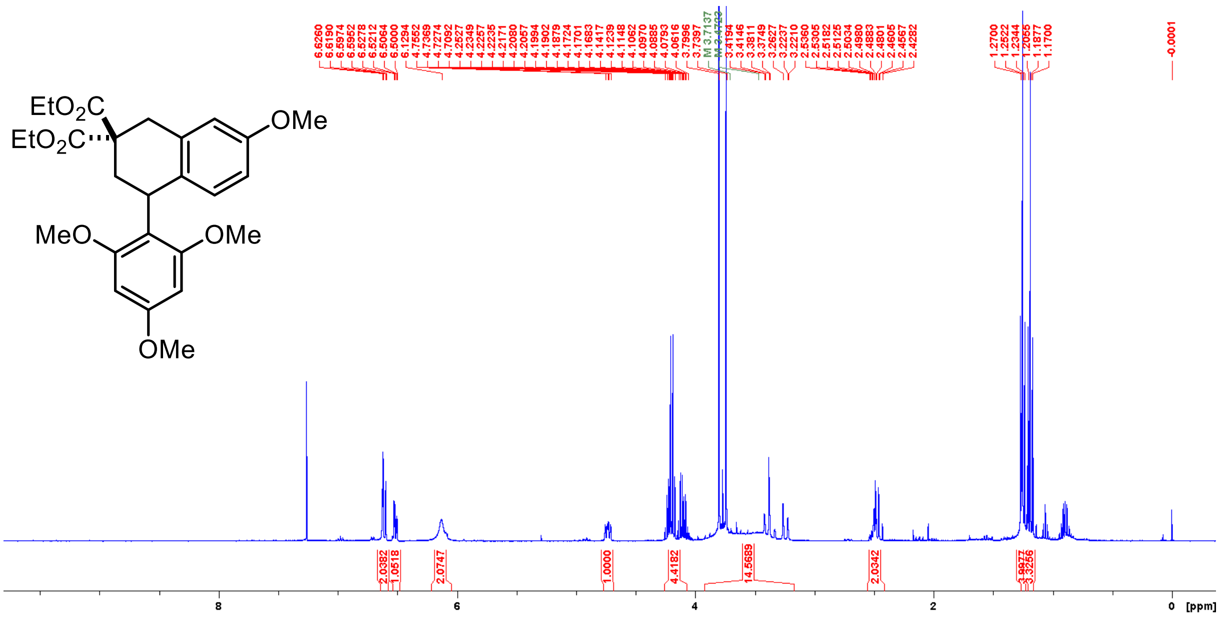

^{13}C{^{1}H} NMR (100 MHz, $CDCl_3$)

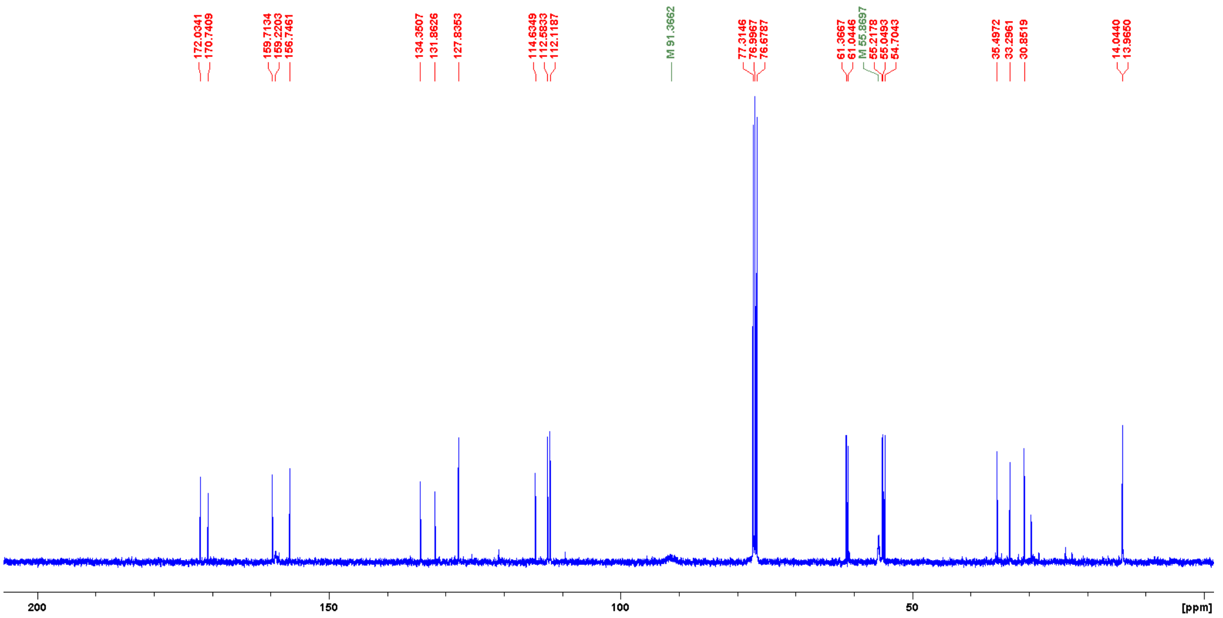

3,3-Bis(ethoxycarbonyl)-7-bromo-1-(2,4,6-trimethoxyphenyl)-1,2,3,4-tetrahydronaphthalene (12j)

^{1}H NMR (400 MHz, $CDCl_3$)

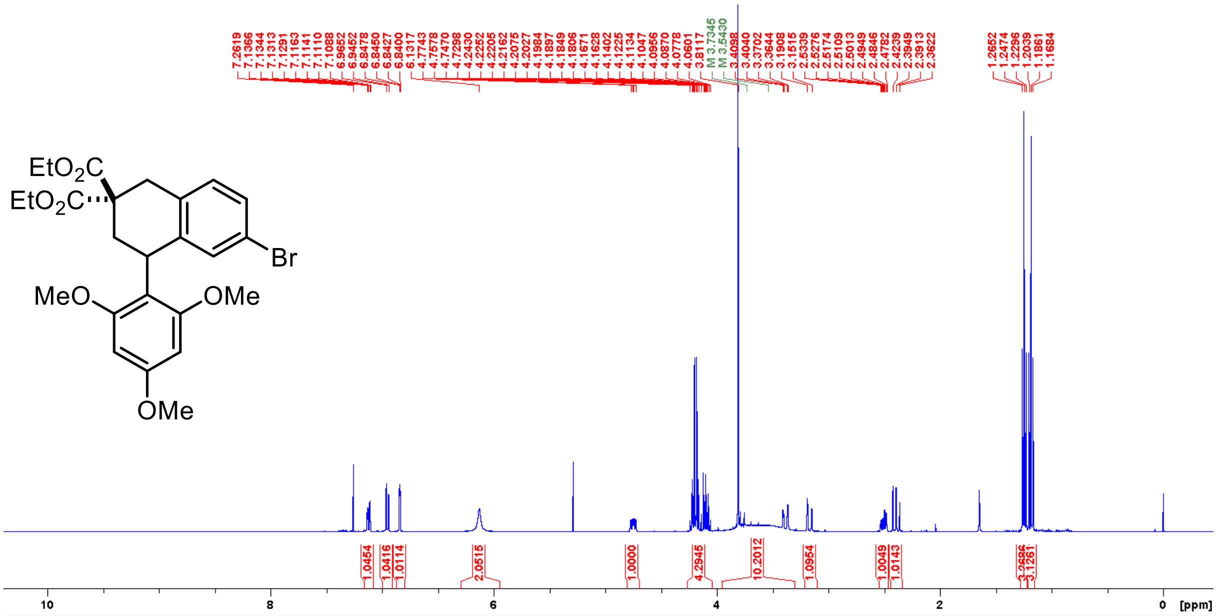

^{13}C{^{1}H} NMR (100 MHz, $CDCl_3$)

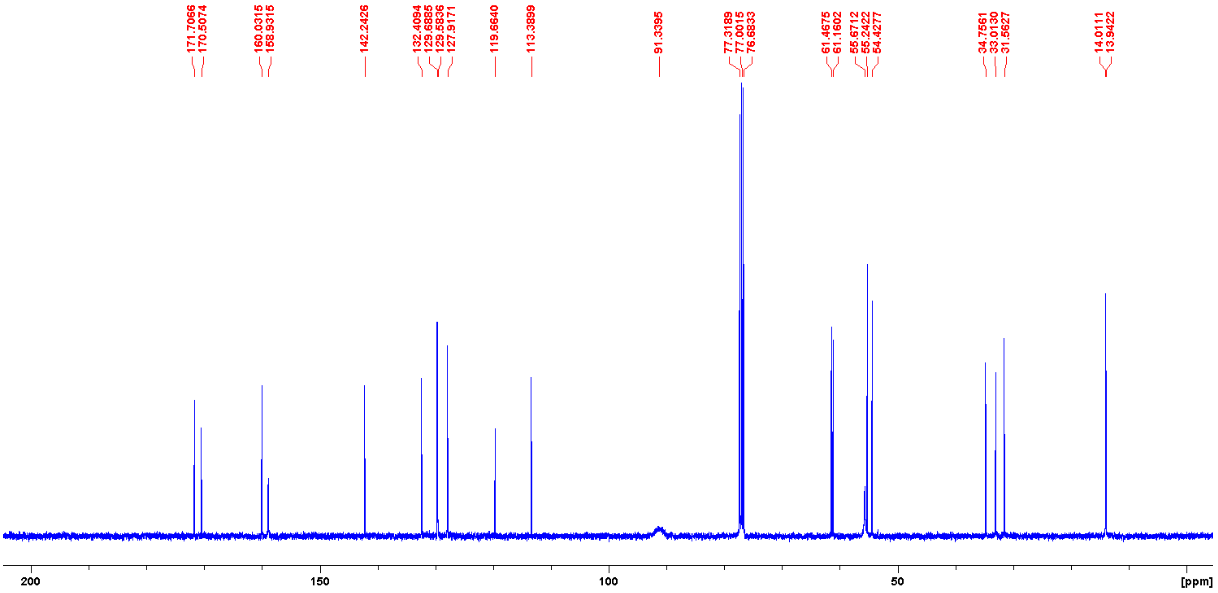

3,3-Bis(ethoxycarbonyl)-7-methoxycarbonyl-1-(2,4,6-trimethoxyphenyl)-1,2,3,4-tetrahydronaphthalene (12k)

^{1}H NMR (400 MHz, $CDCl_3$)

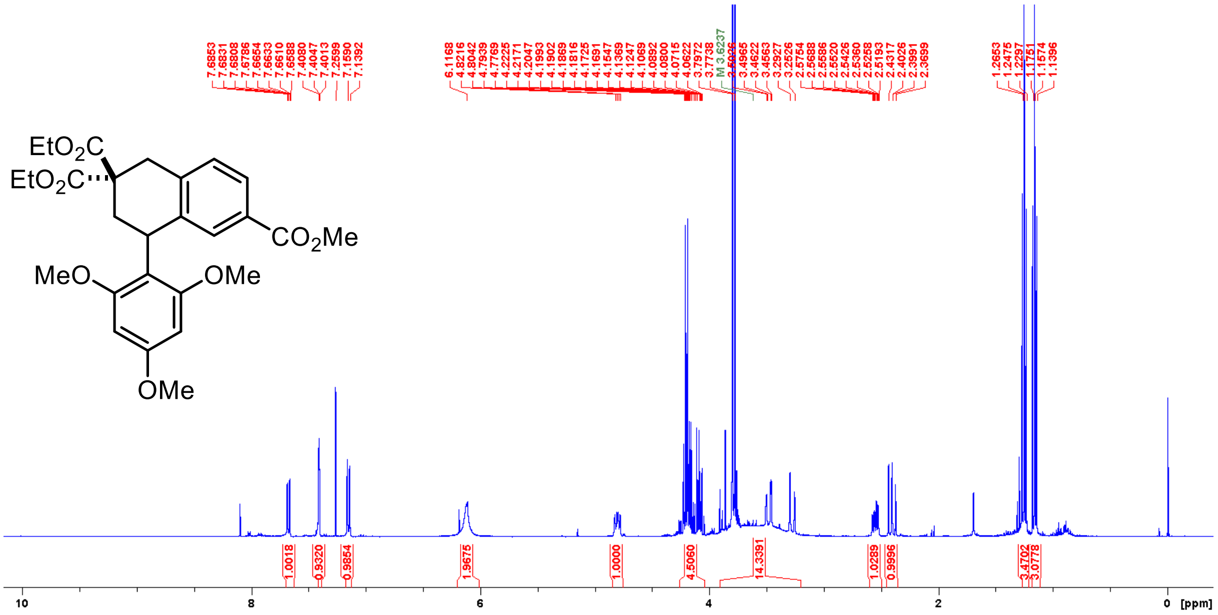

$^{13}C\{^{1}H\}$ NMR (100 MHz, $CDCl_3$)

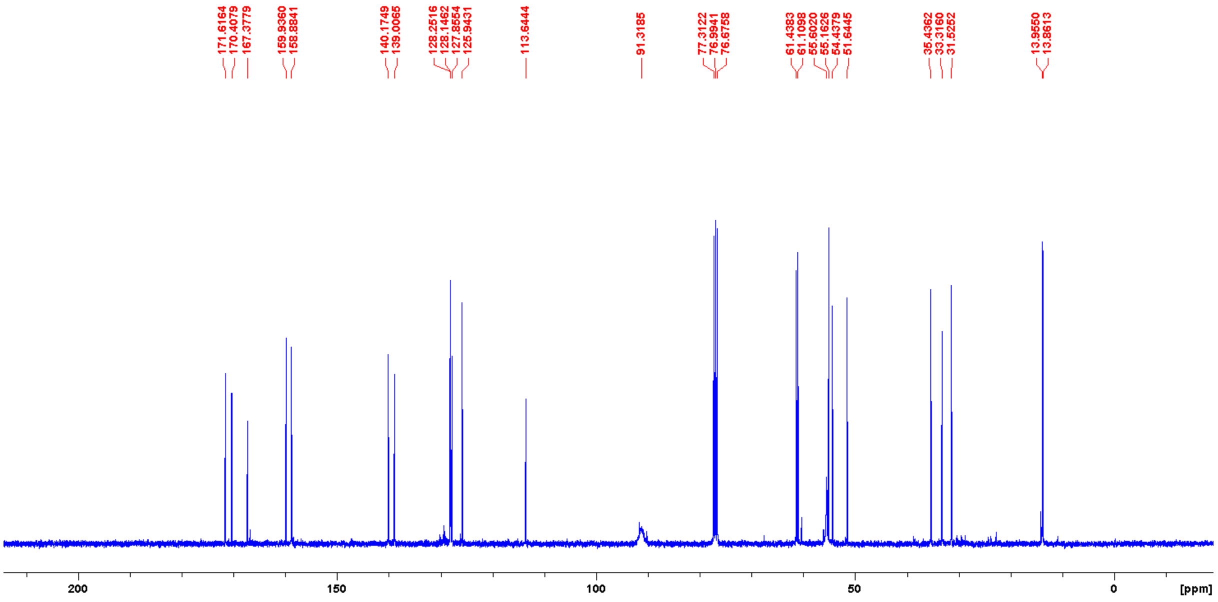

2-[3,3-Bis(ethoxycarbonyl)-4-(4-cyanophenyl)-1-buten-1-yl]-1,3,5-trimethoxybenzene (13m)

^{1}H NMR (400 MHz, $CDCl_3$)

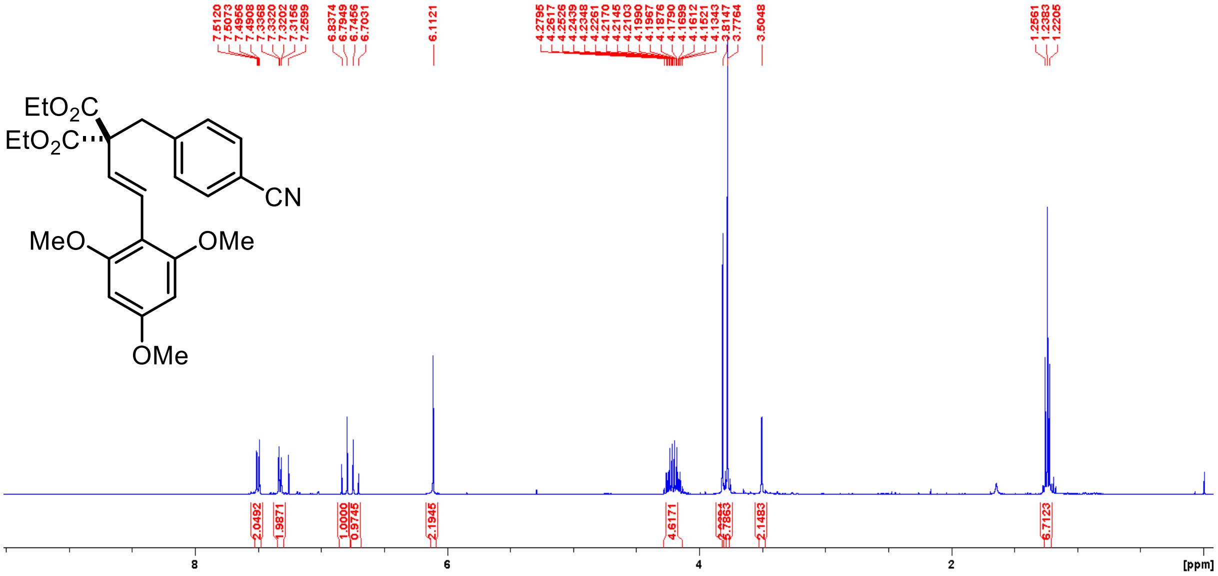

$^{13}C\{^{1}H\}$ NMR (100 MHz, $CDCl_3$)

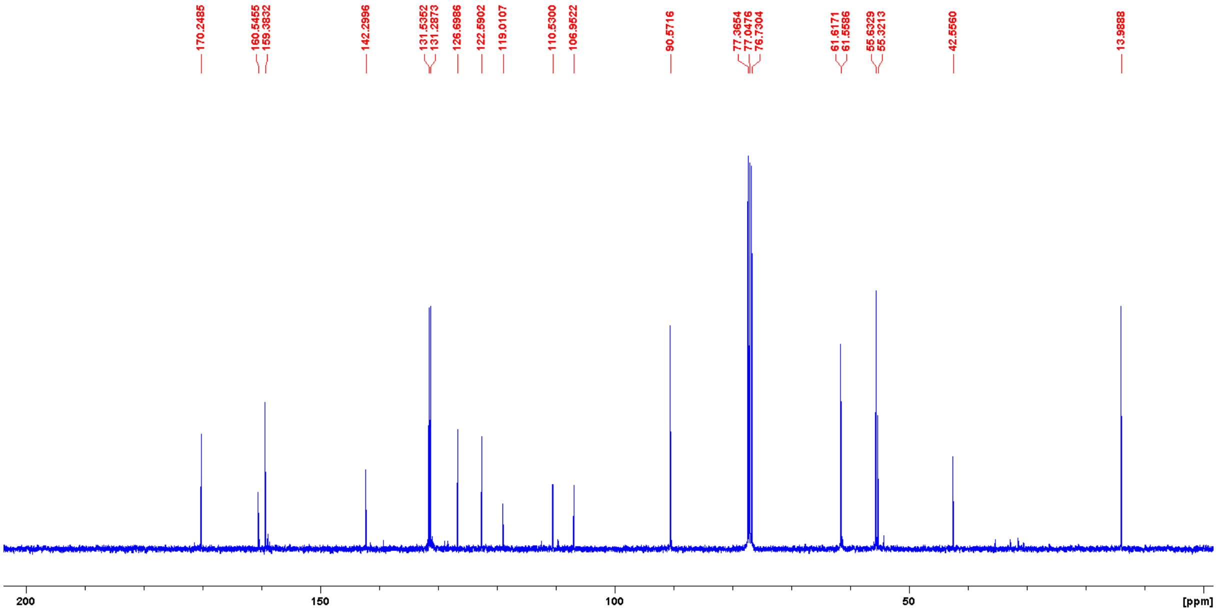

2-[3,3-Bis(ethoxycarbonyl)-4-(4-nitrophenyl)-1-buten-1-yl]-1,3,5-trimethoxybenzene (13n)

^{1}H NMR (400 MHz, $CDCl_3$)

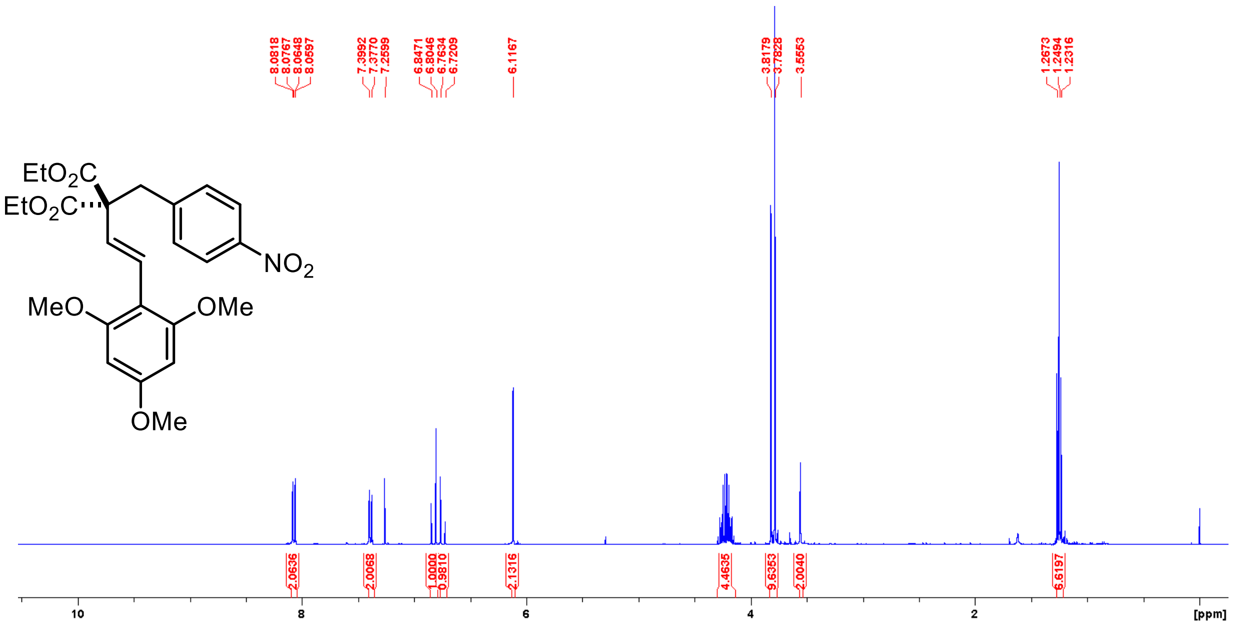

$^{13}C\{^{1}H\}$ NMR (100 MHz, $CDCl_3$)

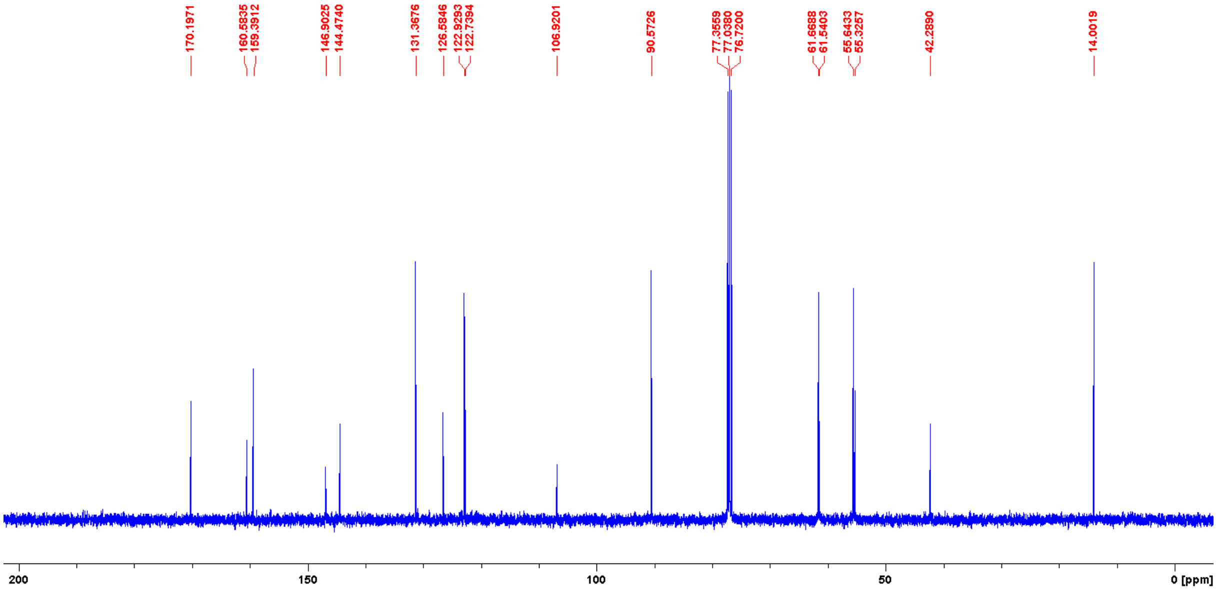

3,3-Bis(ethoxycarbonyl)-1-(2,4-dimethoxyphenyl)-1,2,3,4-tetrahydronaphthalene (14)

^{1}H NMR (400 MHz, $CDCl_3$)

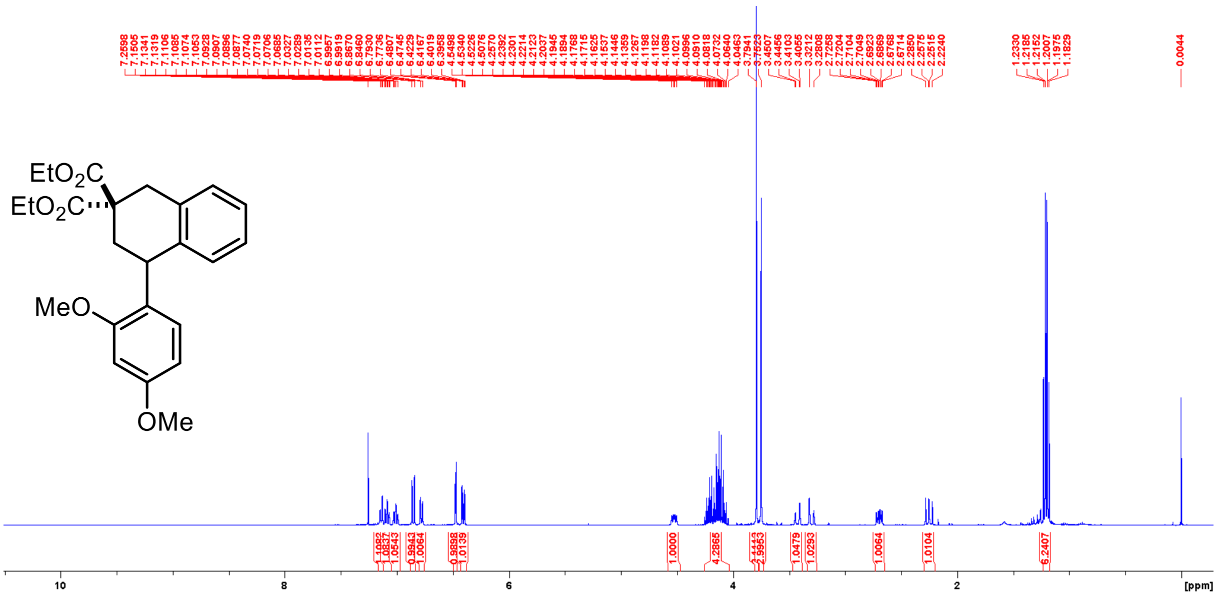

$^{13}C\{^{1}H\}$ NMR (100 MHz, $CDCl_3$)

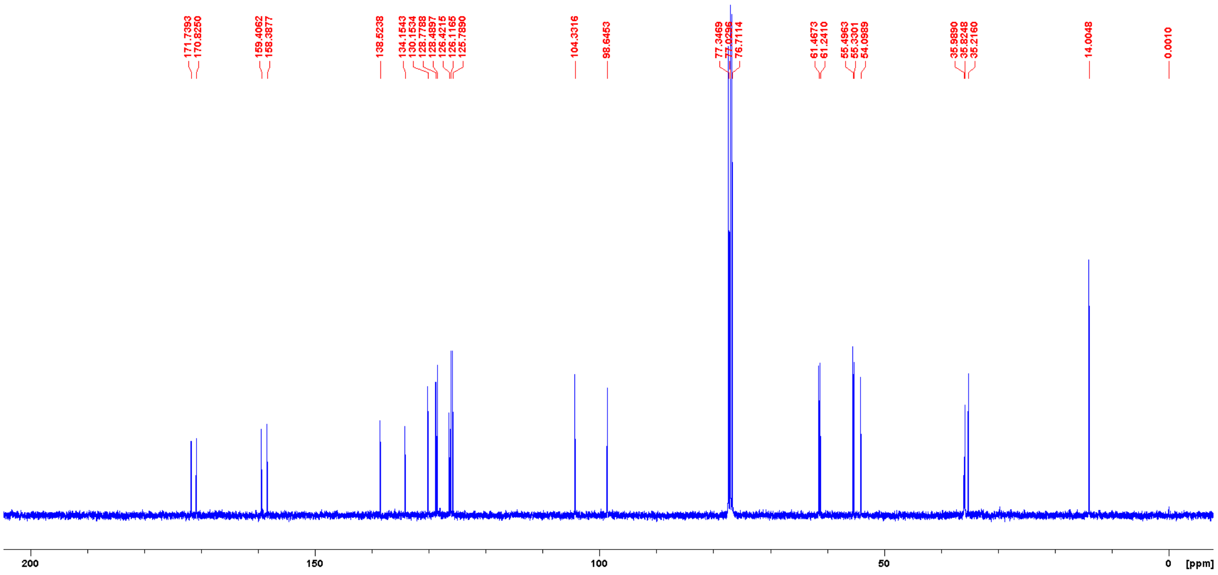

3,3-Bis(ethoxycarbonyl)-1-(3,4,5-trimethoxyphenyl)-1,2,3,4-tetrahydronaphthalene (15)

^{1}H NMR (400 MHz, $CDCl_3$)

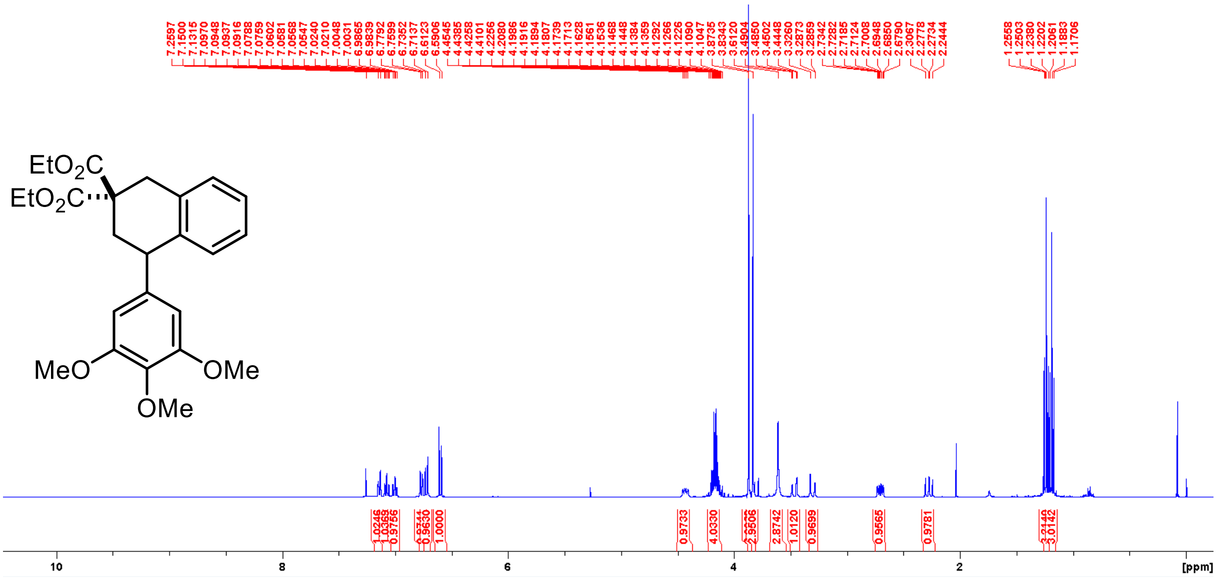

^{13}C{^{1}H} NMR (100 MHz, $CDCl_3$)

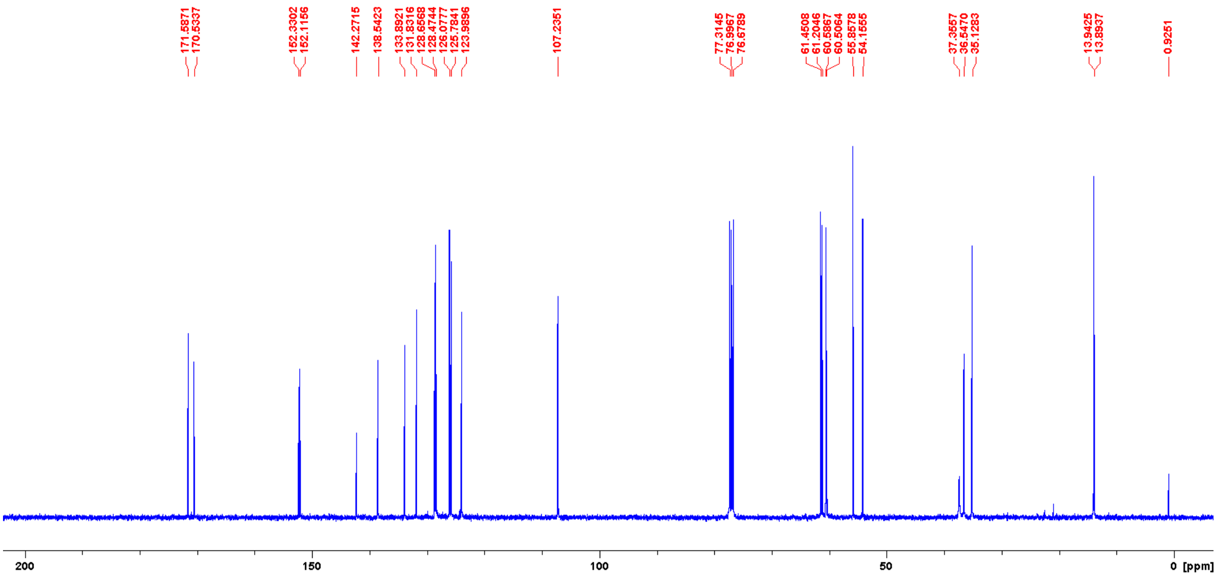

Diethyl (3-methylbut-2-enyl)(phenyl)malonate (16)

^{1}H NMR (400 MHz, $CDCl_3$)

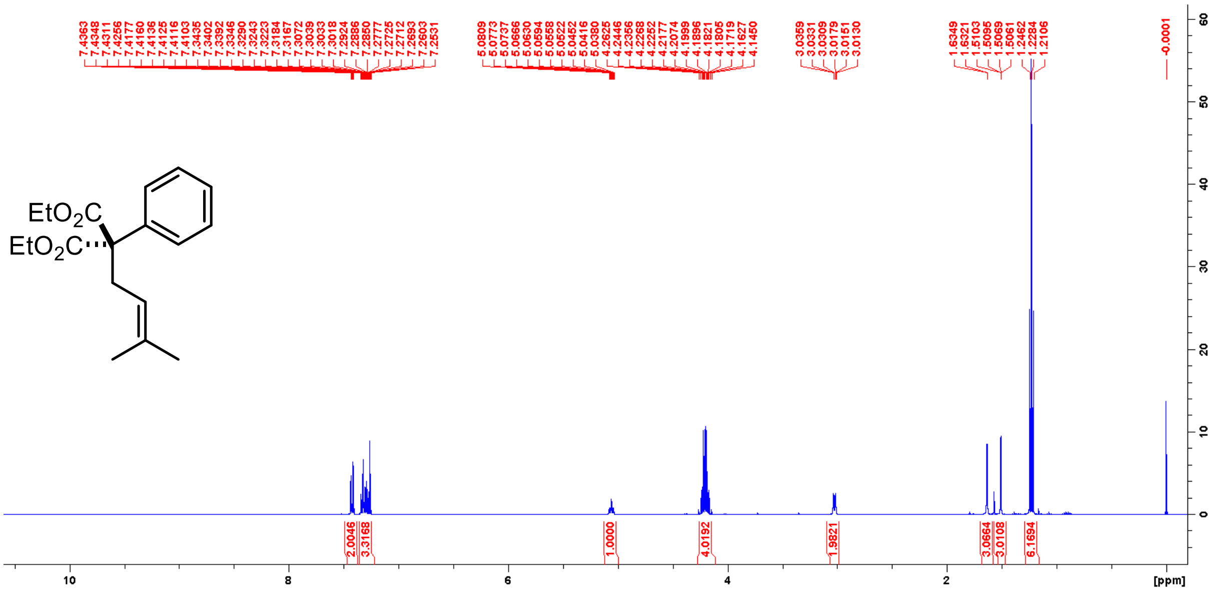

$^{13}C\{^{1}H\}$ NMR (100 MHz, $CDCl_3$)

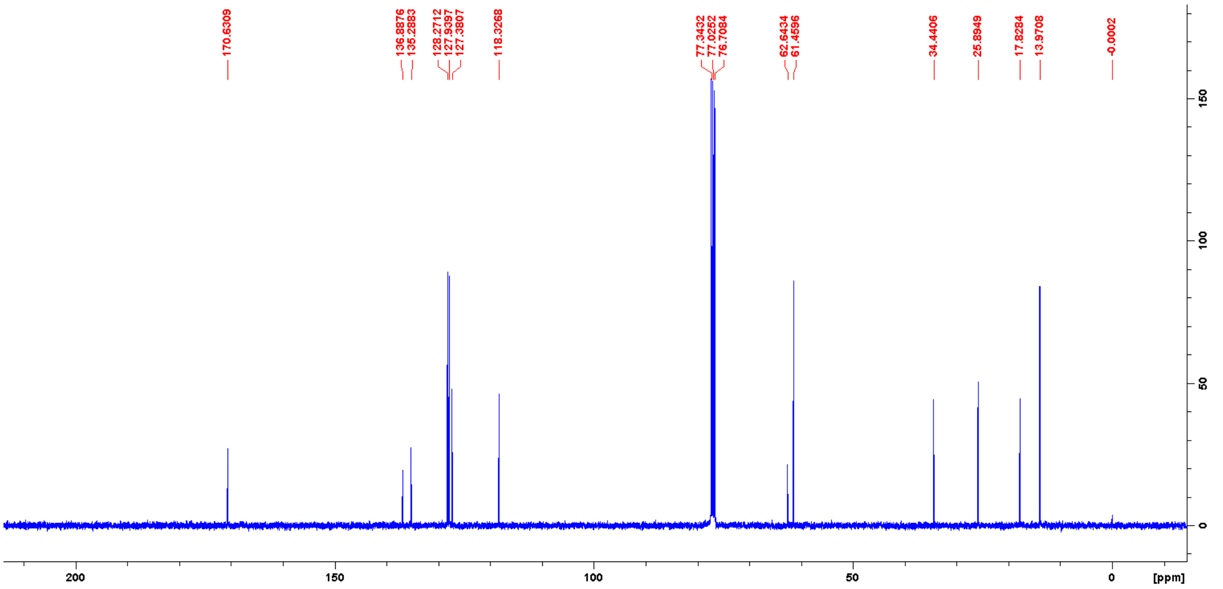

3,3-Bis(ethoxycarbonyl)-1-(2,4,6-trimethoxyphenyl)-1,2-dihydroindene (17)

^{1}H NMR (400 MHz, $CDCl_3$)

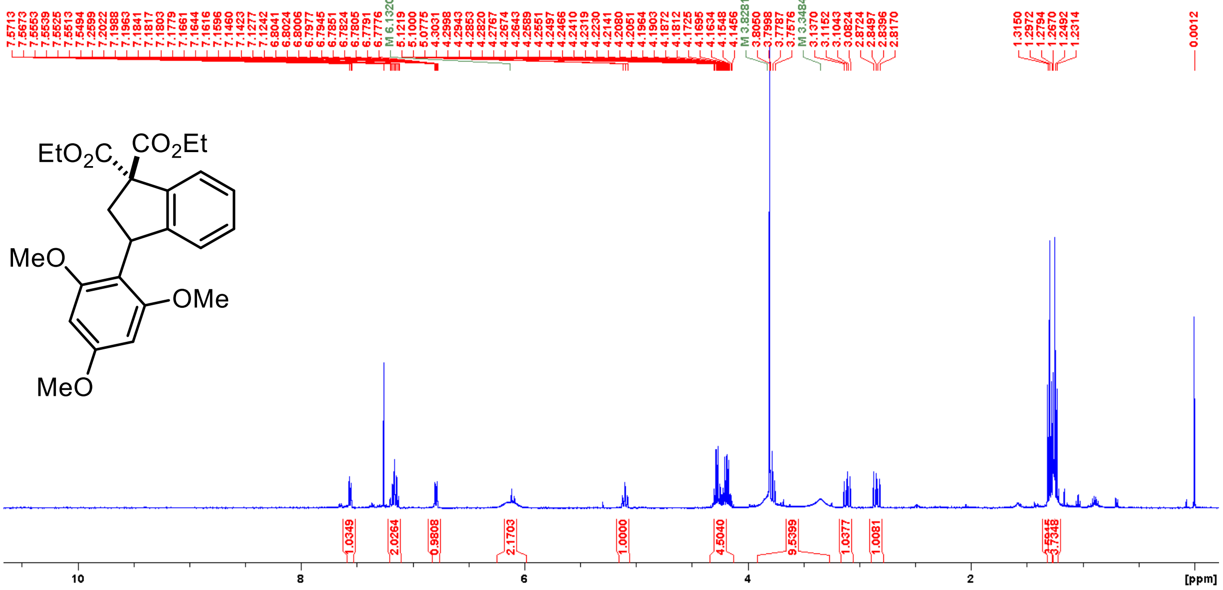

^{13}C{^{1}H} NMR (100 MHz, $CDCl_3$)

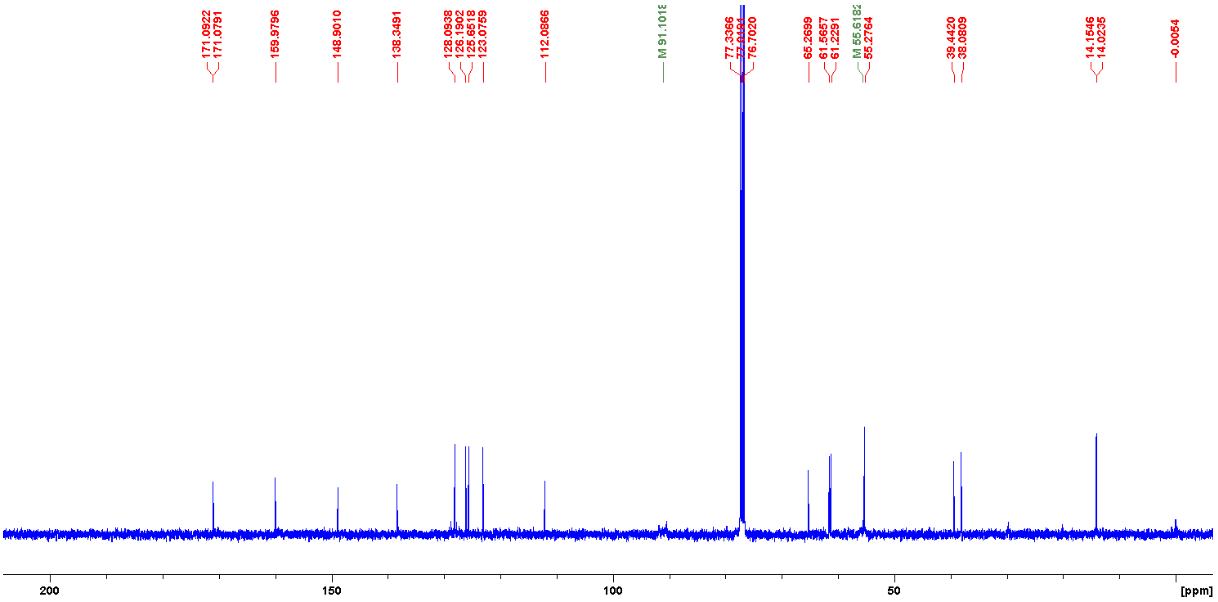

Supplement: File 1 — Experimental procedures, compound characterizations, crystallographic data, DFT calculation, and spectra. [file Beilstein_J_Org_Chem-20-162-s001.pdf]
